# Supplementary material for: The translocation assembly module (TAM) catalyzes the assembly of bacterial outer membrane proteins in vitro
Source: Nat Commun. 2024 Aug 23;15:7246. doi: 10.1038/s41467-024-51628-8 (PMC11341756; doi:10.1038/s41467-024-51628-8)
Supplement: Supplementary file 1 — Supplementary Information [file 41467_2024_51628_MOESM1_ESM.pdf]

Supplementary Information for

The translocation assembly module (TAM) catalyzes the assembly of bacterial outer membrane proteins *in vitro*

Xu Wang, Sarah B. Nyenhuis, and Harris D. Bernstein

SUPPLEMENTARY FIGURES

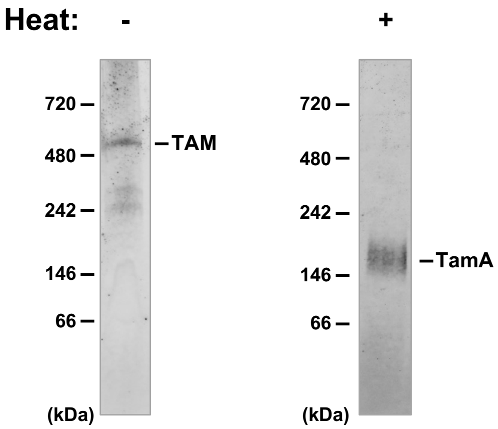

Supplementary Fig. 1. Native PAGE analysis of TAM/PLE proteoliposomes. Samples were prepared using the NativePAGE Sample Prep Kit (Thermo Fisher, catalog number BN2008). TAM/PLE proteoliposomes were either mixed with 1% DDM and kept on ice or mixed with 50 mM DTT and heated at 95° C for 10 min. Proteins were resolved on 4 to 16% NativePAGE Bis-Tris mini gels (Thermo Fisher, catalog number BN1002). (Proteins could not be resolved by blue native PAGE if samples were left unheated in the presence of DTT or heated without adding DTT presumably due to the presence of cysteines in TamB and the characteristics of the proteoliposomes). The molecular weight was determined using the NativeMark Unstained Protein Standard (Thermo Fisher, catalog number LC0725). Proteins resolved by blue native PAGE were first transferred to a PVDF membrane and stained by Coomassie Brilliant blue R-250 to visualize the molecular weight markers. The membrane was then destained and TamA was detected by Western blot using an antiserum raised against the His tag. It should be noted that after heating TamA migrated at ~150 kDa based on the NativeMark molecular weight markers, but migrated at ~65 kDa based on conventional SDS-PAGE markers (Chameleon Duo pre-stained protein markers; LICOR, catalog number 928-60000; shown in the Source Data file, p. 59). This analysis was perfomed three times with similar results.

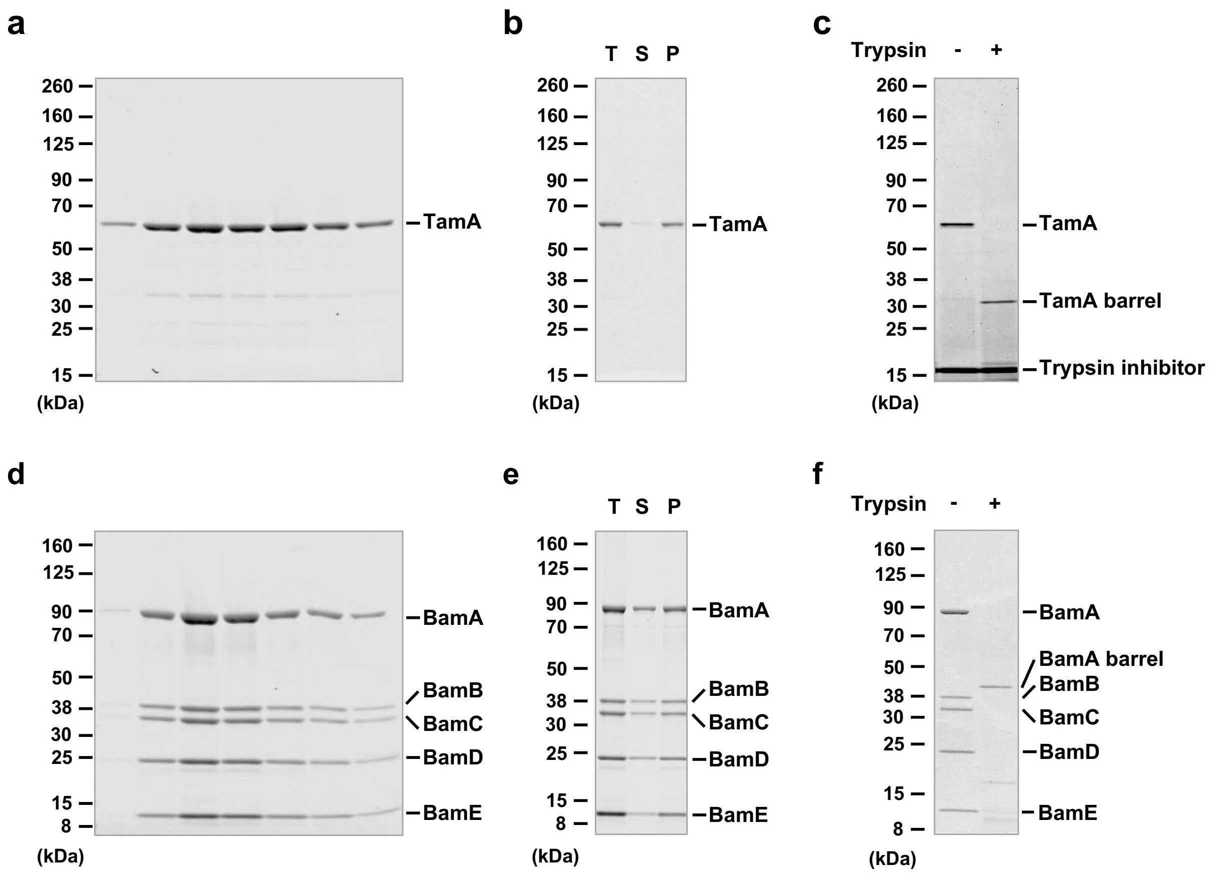

Supplementary Fig. 2. Purification and reconstitution of TamA and BAM. a and **d** SDS-PAGE analysis of fractions eluted from Ni-NTA agarose. In **a**, His_8-TamA was purified from cells transformed with pXW49, and in **d**, BamABCDE-His_8 was purified from cells transformed with pYG120. **b** and **e** Purified TamA or BAM was mixed with PLE liposomes. The total mixture (T) was ultracentrifuged to generate a supernatant (S), which contains free protein, and a pellet (P), which contains reconstituted protein. Samples were analyzed by SDS-PAGE and Coomassie blue staining. **c** and **f** TamA/PLE or BAM/PLE proteoliposomes were treated with trypsin to digest exposed proteins and protein segments or untreated. The reaction was stopped by adding a trypsin inhibitor **c** or by heating to 95° C for 10 min **f** and analyzed by SDS-PAGE and Coomassie blue staining. The low molecular weight (~10 kDa) bands seen in the presence of trypsin in **f** have been observed previously[1]. TamA and BAM were purified and reconstituted into proteoliposomes three and five times, respectively, with similar results.

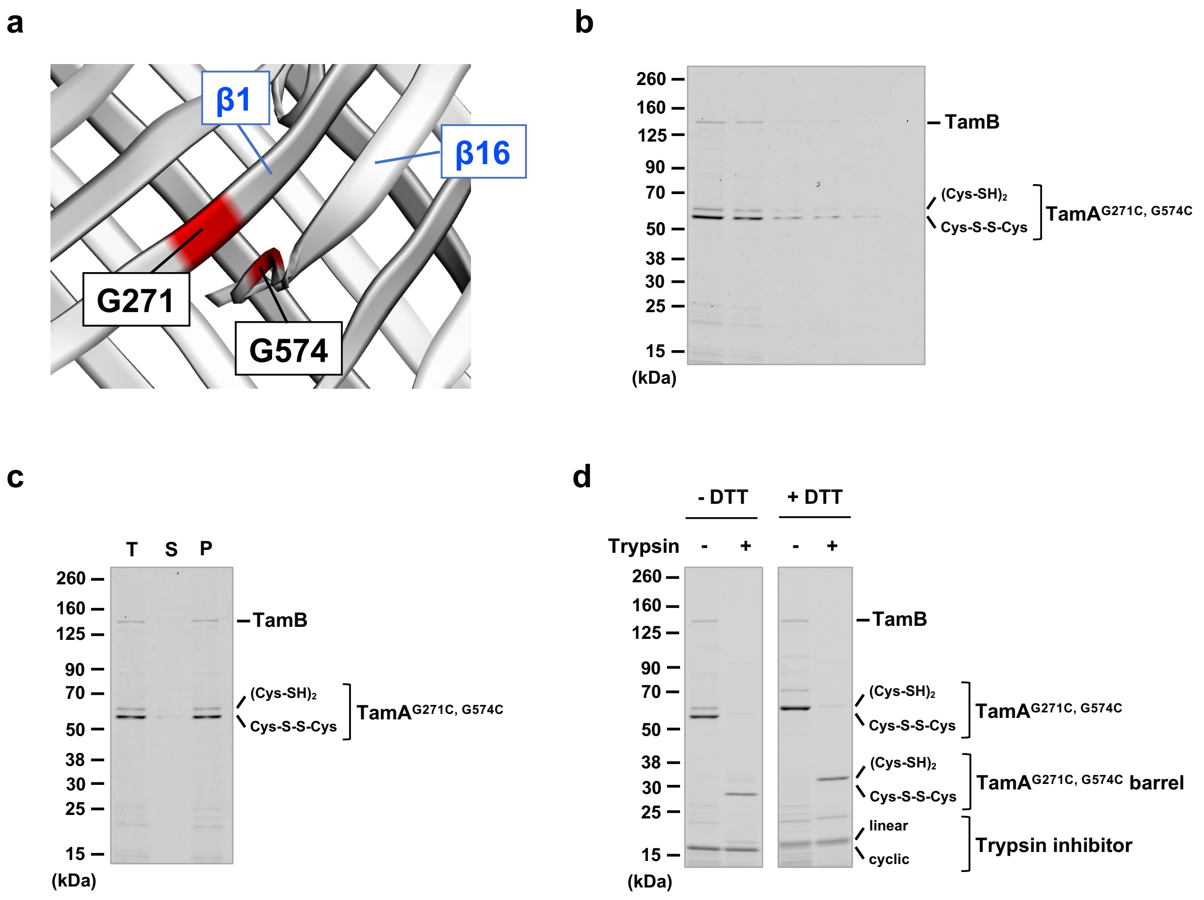

Supplementary Fig. 3. Purification and reconstitution of TamA$^{G271C, G574C}$-TamB. His$_8$-TamA$^{G271C, G574C}$-TamB was expressed in BL21-CodonPlus(DE3)-RIPL transformed with pXW50 and purified using the same protocol that was used to purify TAM. The two residues that were mutated to cysteine are highlighted in **a**. **b** SDS-PAGE analysis of fractions eluted from Ni-NTA agarose. The two bands that migrated between 50kDa and 70kDa correspond to two TamA$^{G271C, G574C}$ populations that contained reduced cysteines (Cys-SH SH-Cys) or oxidized cysteines (Cys-S-S-Cys). **c** Purified TAM$^{G271C, G574C}$ was mixed with PLE liposomes. The total mixture (T) was ultracentrifuged to generate a supernatant (S), which contains free protein, and a pellet (P), which contains reconstituted protein. Samples were analyzed by SDS-PAGE and Coomassie blue staining. The data show that both oxidized and reduced forms of TamA$^{G271C, G574C}$ can be reconstituted into PLE. **d** TAM$^{G271C, G574C}$-TamB PLE proteoliposomes were treated

with trypsin to digest exposed proteins and protein segments or untreated. The reaction was stopped by adding a trypsin inhibitor and mixing half of each sample with 50 mM DTT. The samples were heated at 95° C for 10 min and analyzed by SDS-PAGE and Coomassie blue staining. DTT reduced the cyclic trypsin inhibitor to a linear molecule. The data confirm that the faster migrating band in the 50-70 kDa range is the oxidized form of $TamA^{G271C, G574C}$ and that only the POTRA domains of $TamA^{G271C, G574C}$ are exposed to digestion. The faint bands migrating at 20kDa and 70kDa in lanes 3-4 were observed in TAM/PLE samples after the addition of DTT as well. $TamA^{G271C, G574C}$-TamB was purified and reconstituted into proteoliposomes twice with similar results.

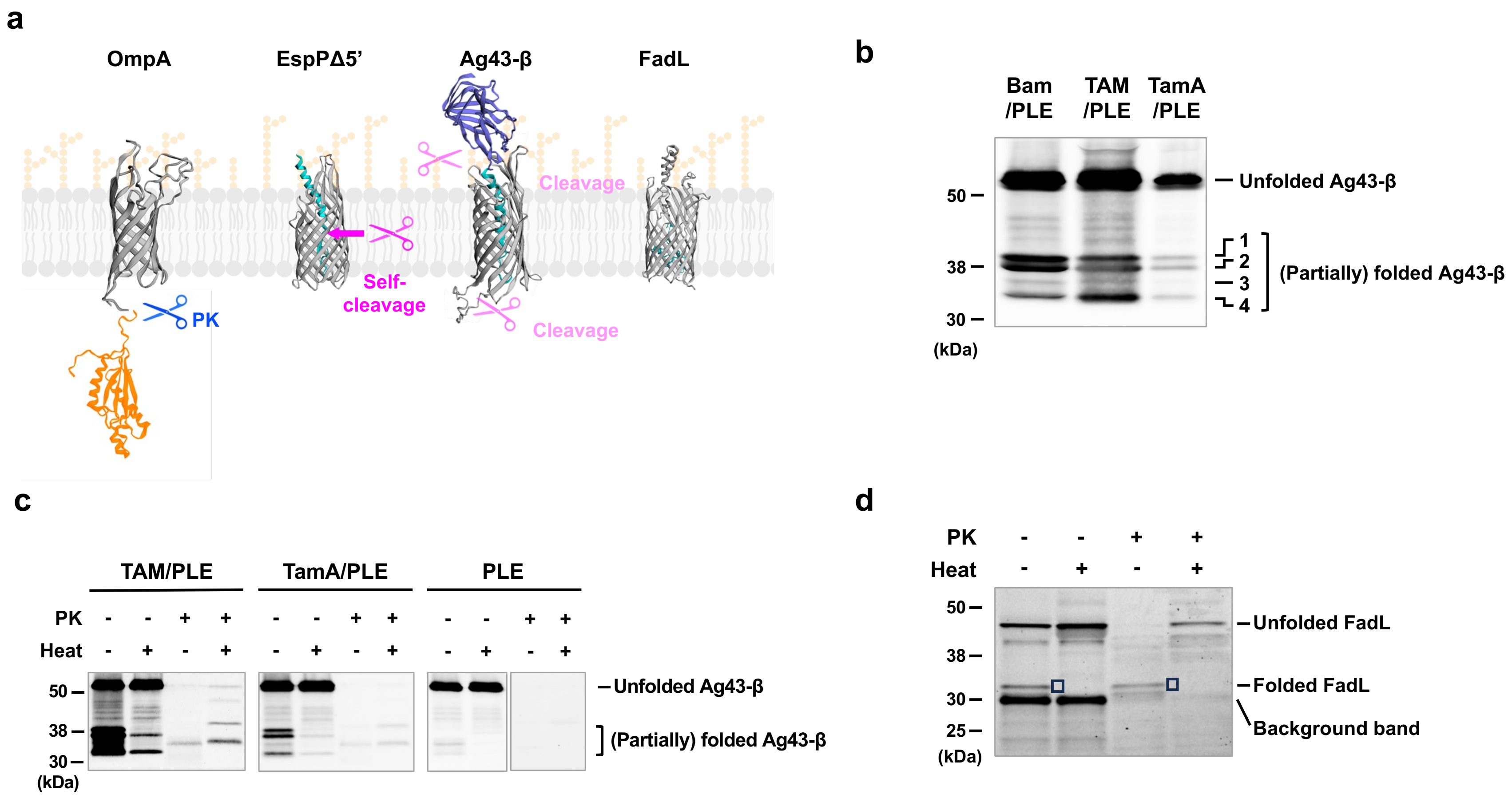

Supplementary Fig. 4. Experiments related to Fig. 3. a The structures of OmpA (PDB 1G90 for residues 22-197; PDB 2MQE for residues 201-346), EspPΔ5' (PDB 3SLO), Ag43-β (predicted by AlphaFold), and FadL (PDB 1T16) are shown[2–7]. The models are

plotted by the EzMol server[8]. The putative PK digestion site in OmpA, the known intra-barrel cleavage site in EspPΔ5'[9] and the putative self-cleavage sites in Ag43-β[10] are indicated. The illustration was created with BioRender.com, released under a Creative Commons Attribution-NonCommercial-NoDerivs 4.0 International license. **b** The experiment shown in Fig. 3c was repeated, but samples were collected only after a 60 min incubation at 30° C, PK was not added, and the samples were not heated. The gel was expanded to show the four folded (or partially folded) forms of Ag43-β more clearly. **c** Part of the experiment shown in Fig. 3c was repeated. Aliquots were collected from each reaction after 60 min, treated with PK, or left untreated. Samples were mixed with loading buffer and placed on ice or heated to 95° C and resolved by SDS-PAGE. The Western blot was overexposed to show the Ag43-β that was assembled by TamA more clearly. The data show that the PK-resistance patterns of Ag43-β assembled into TAM/PLE and TamA/PLE proteoliposomes were similar. **d** FadL was synthesized *de novo* in the PURExpress coupled transcription/translation system (New England BioLabs, catalog number E6800L) using the plasmid pET303::*fadL26-446*. The reaction was supplemented with BODIPY-FL-ε-Lys-tRNALys (Promega, catalog number L5001) to fluorescently label FadL, 2 μM SurA and 2 μM Bam/POPC proteoliposomes to analyze BAM-mediated folding of FadL as described previously[11]. The reaction was incubated at 37° C for 1 h. Samples were subjected to PK digestion or left untreated, and then heated to 95° C or left unheated. Proteins were resolved by SDS-PAGE and visualized using an Amersham Typhoon scanner at an excitation wavelength of 488 nm. Folded FadL is denoted with a square. The experiments shown in **b** and **c** were performed three times, and the experiment shown in **d** was performed twice with similar results.

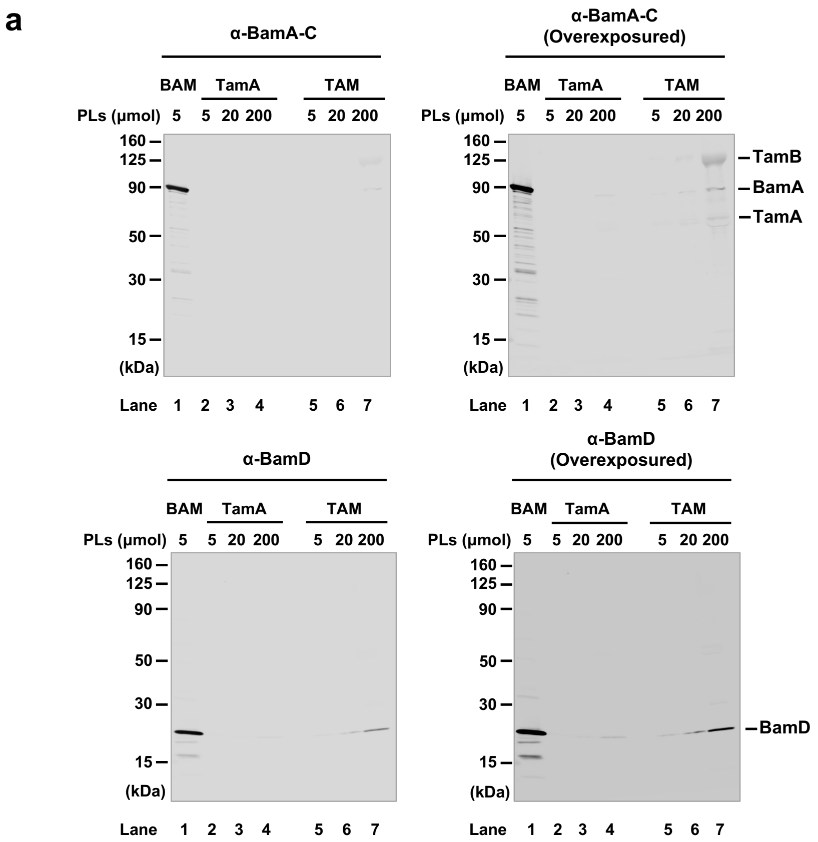

	TamA/PLE	TAM/PLE
BamA	<0.005%	<0.080%
BamD	<0.139%	<0.645%

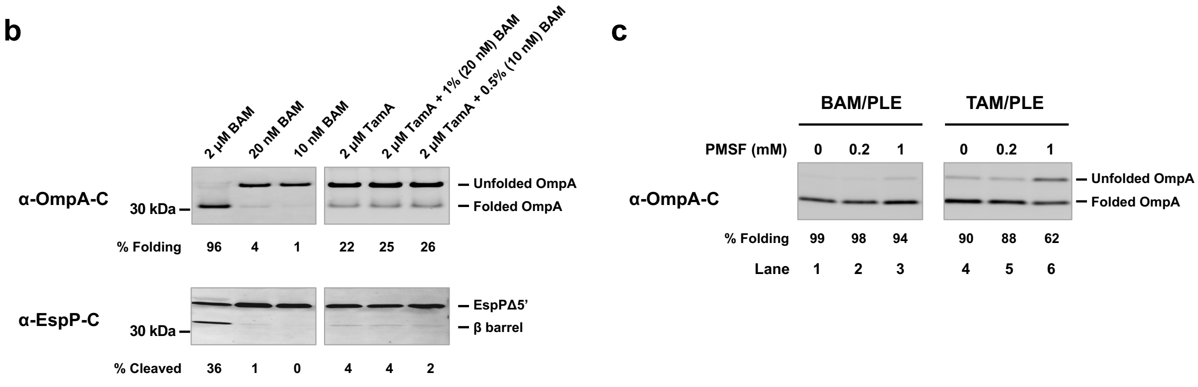

Supplementary Fig. 5. BAM co-purified with TAM does not significantly contribute to TAM-mediated OMP assembly. a As much as 200 μmol TamA (in TamA/PLE) and TAM (in

TAM/PLE) were heated to 95° C in SDS sample buffer, and proteins were resolved by SDS-PAGE. BamA and BamD that co-purified with TamA and TAM were visualized by Western blot using the indicated antiserum. The percent of BamA and BamD in the TamA and TAM proteoliposome preparations (shown in the table) was calculated by comparing the signal in the lanes that contained 200 μmol TamA or TAM (lanes 4 and 7) to the signal generated by a 5 μmol BAM standard (lane 1). **b** Urea-denatured OmpA or EspPΔ5’ (0.2 μM) were incubated with the indicated concentrations of BAM/PLE and/or TamA/PLE proteoliposomes at 30° C for 60 min. After adding SDS-PAGE loading buffer, OmpA samples were placed on ice, and the EspPΔ5’ samples were heated to 95° C for 10 min. Proteins were resolved by SDS-PAGE and visualized by Western blot using the indicated antisera. Percent folding (or percent cleaved, which is equivalent to percent folded for EspPΔ5’) was determined as in Fig. 4. The data show that the level of BAM that was present in TAM/PLE and TamA/PLE proteoliposomes (<0.7% of the total protein) was insufficient to account for the observed OmpA assembly. **c** Urea-denatured OmpA (0.2 μM) and BAM/PLE or TAM/PLE proteoliposomes (2 μM) were incubated with the indicated amount of PMSF at 30° C for 60 min. OmpA samples were mixed with SDS-PAGE loading buffer and placed on ice for 10 min. OmpA was visualized by Western blot using the anti-OmpA-C terminal peptide antiserum and quantitated as described in part **b**. The data confirm that the TAM that is present in TAM-PLE mediates OmpA assembly because only TAM activity, but not BAM activity, is inhibited by PMSF.

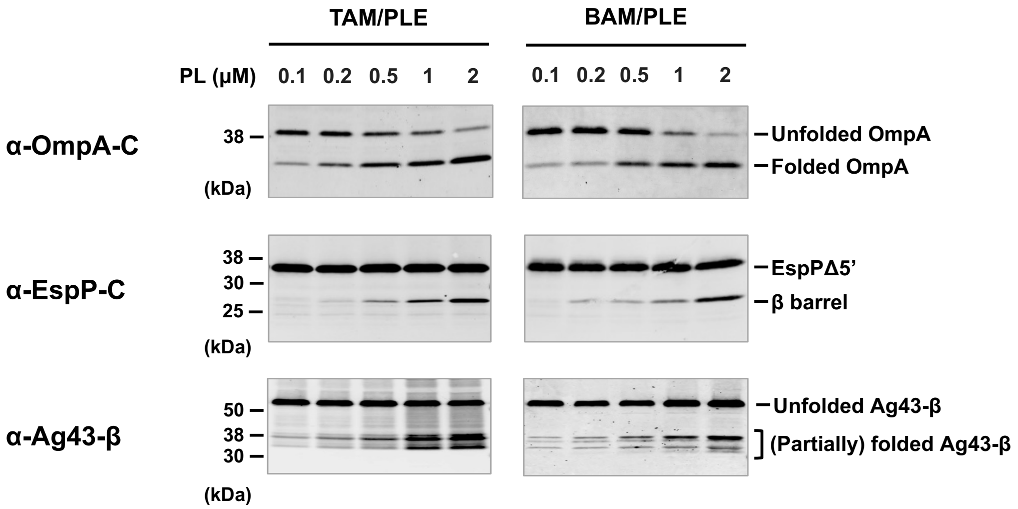

Supplementary Fig. 6. TAM can fold OMPs at low concentrations *in vitro*. Urea-denatured OMPs (0.2 μM) were incubated with the indicated concentrations of TAM/PLE or BAM/PLE proteoliposomes at 30° C for 60 min. After adding the loading buffer, OmpA and Ag43-β samples were placed on ice, whereas EspPΔ5’ samples were heated to 95° C for 10 min. Proteins were resolved by SDS-PAGE and OMP folding was assessed by Western blot using appropriate antisera. These experiments were performed twice with similar results.

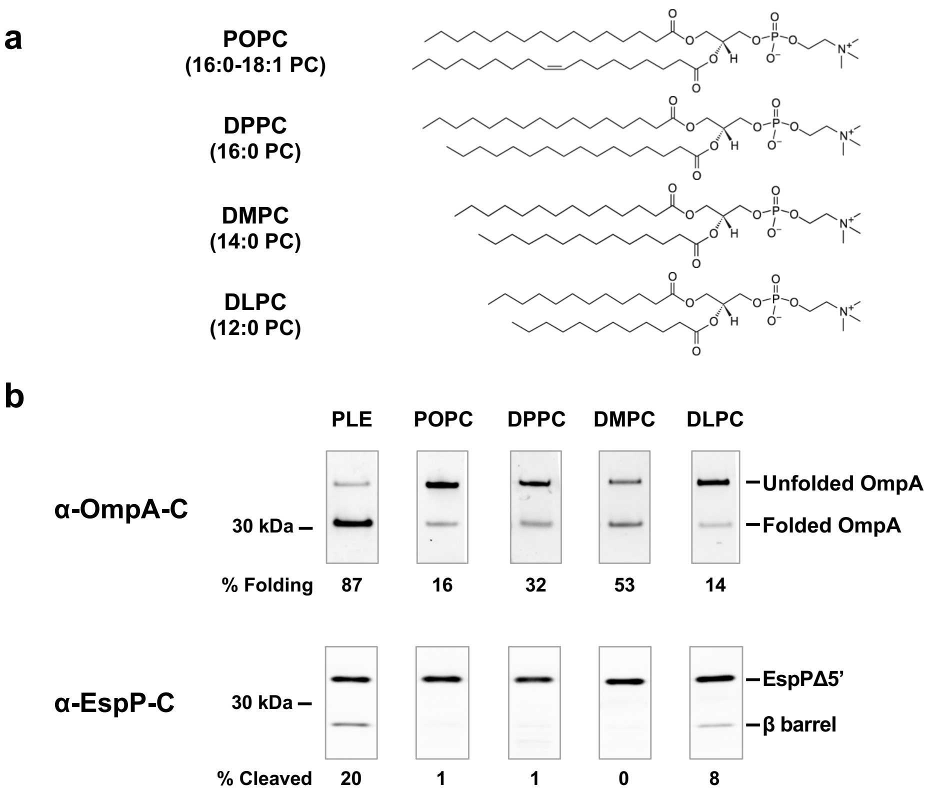

Supplementary Fig. 7. TAM reconstituted into PLE shows the highest folding activity *in vitro*. a Chemical structures of the synthetic phospholipids used in this assay. All of the phospholipids were obtained from Avanti Polar Lipids: 1-palmitoyl-2-oleoyl-glycero-3-phosphocholine (POPC, catalog number 850457), dipalmitoylphosphatidylcholine (DOPC, catalog number 850375), 1,2-dimyristoyl-sn-glycero-3-phosphocholine (DMPC, catalog number 850345), 1,2-dilauroyl-sn-glycero-3-phosphocholine (DLPC, catalog number 850335). **b** Urea denatured OmpA or EspPΔ5' was incubated with 2 μM TAM proteoliposomes containing the indicated lipids at 30° C for 60 min. Unheated OmpA or heated EspPΔ5' samples were subjected to SDS-PAGE and folding was assessed by Western blot using the antisera raised against either an OmpA or EspP C-terminal peptide. These experiments were performed three times with similar results.

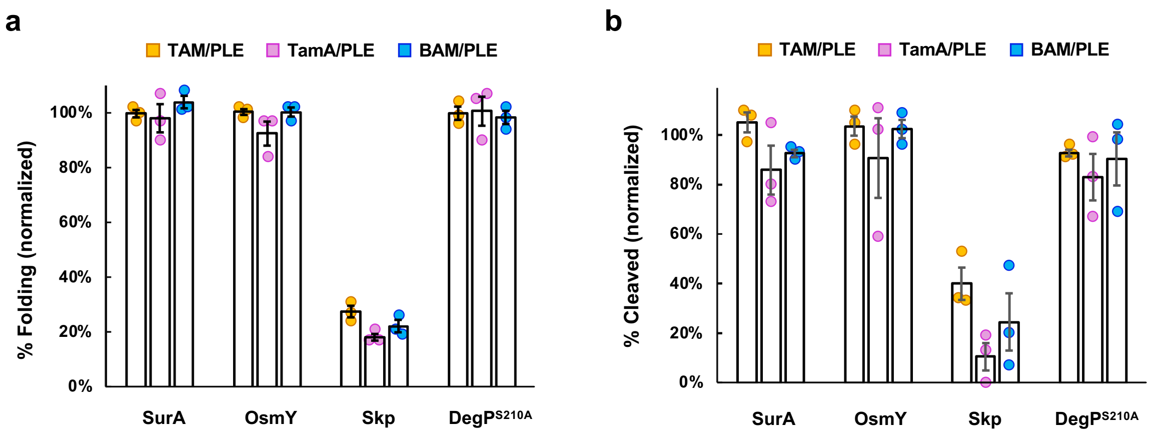

Supplementary Fig. 8. Quantitation of the effect of chaperones on the assembly of OMPs into TAM/PLE, TamA/PLE and BAM/PLE proteoliposomes. The effect of the indicated chaperones on the assembly of OmpA **a** and EspPΔ5' **b** into TAM/PLE, TamA/PLE and BAM/PLE proteoliposomes was analyzed in three independent experiments. Percent folding (for OmpA) and percent cleaved (for EspPΔ5') was determined as described in the legend to Fig. 4. Values were then normalized to the percent folding or percent cleaved observed in the control (i.e., in the absence of a chaperone) which was defined as 100%. Error bars represent the standard error of the mean.

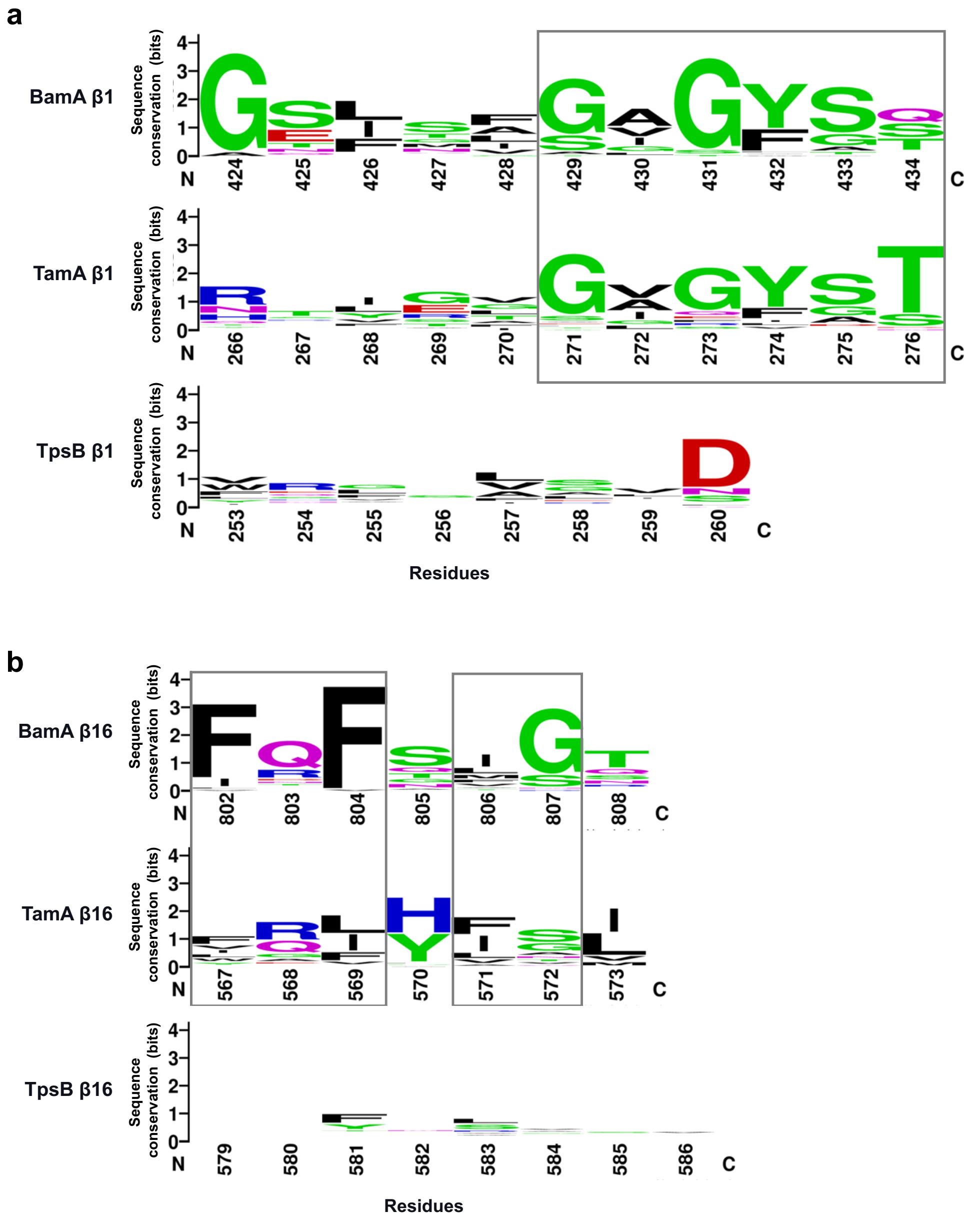
a
BamA β1
TamA β1
TpsB β1
Sequence conservation (bits)
N
424 425 426 427 428 429 430 431 432 433 434
C
266 267 268 269 270 271 272 273 274 275 276
253 254 255 256 257 258 259 260
Residues
b
BamA β16
TamA β16
TpsB β16
802 803 804 805 806 807 808
567 568 569 570 571 572 573
579 580 581 582 583 584 585 586
Residues

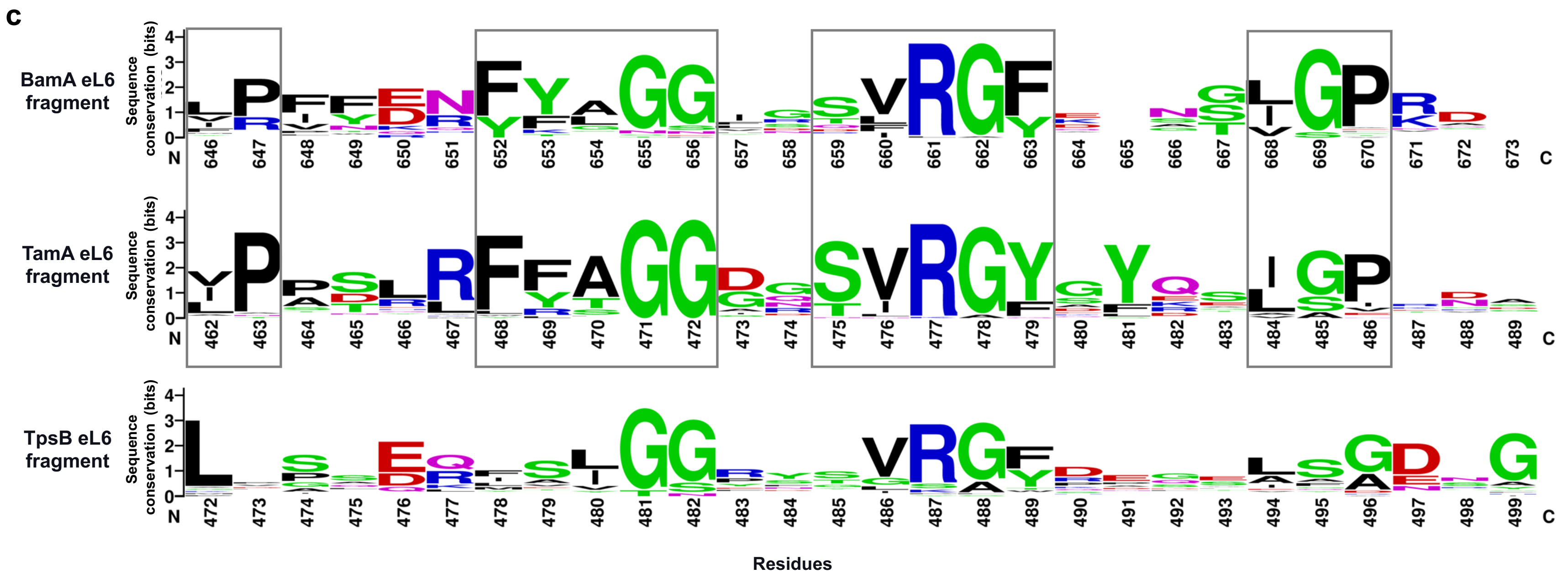

Supplementary Fig. 9. Sequence logo plots for BamA, TamA, and TpsB proteins from 39 Proteobacterial families. The logo plots show the sequence conservations of the residues in **a** the first β stand (β1), **b** the last β stand (β16), and **c** a fragment of the extracellular loop 6 (eL6) of the indicated protein β barrels. The organisms that were used for this analysis are listed in Supplementary Table 2. β1 and β16 were identified based on the structures of *E. coli* K-12 BamA (PDB: 8BVQ), *E. coli* K-12 TamA (PDB: 4C00), and *E. coli* O104:H21 ShlB (a TpsB protein whose structure was predicted by AlphaFold)[5,6,12,13]. β1, β16, and eL6 in other proteins were determined by combining AlphaFold predictions[5,6] and multiple protein alignments (COBALT)[14] and checked manually for any misalignment. Sequence logos were plotted using Weblogo 3[15]. The x-axis shows the residue numbers of the *E. coli* proteins. The segments conserved in both BamA and TamA are boxed.

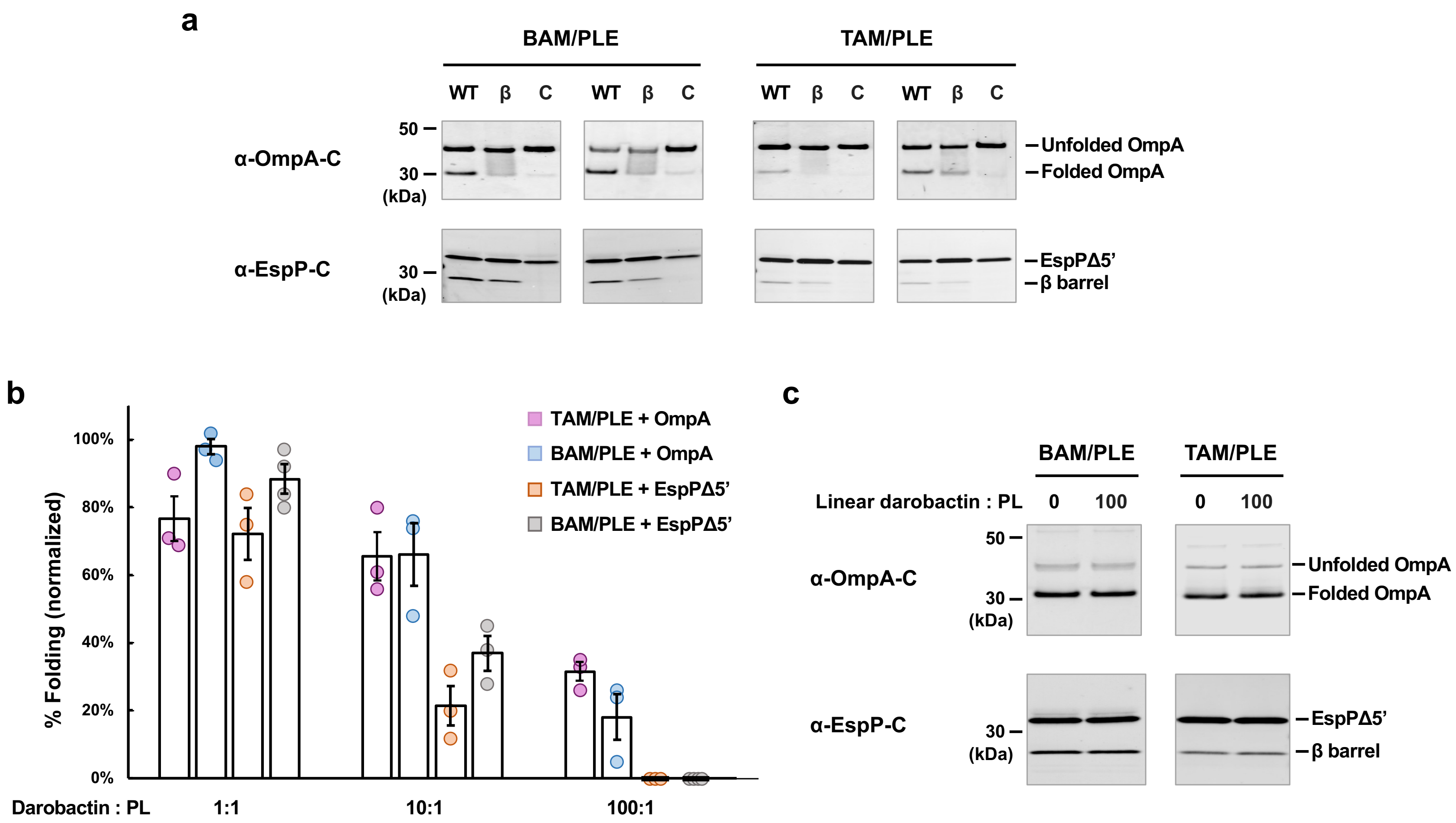
a
BAM/PLE
TAM/PLE
WT β C WT β C
WT β C WT β C
α-OmpA-C
50
30
(kDa)
Unfolded OmpA
Folded OmpA
α-EspP-C
30
(kDa)
EspPΔ5'
β barrel
b
% Folding (normalized)
100%
80%
60%
40%
20%
0%
TAM/PLE + OmpA
BAM/PLE + OmpA
TAM/PLE + EspPΔ5'
BAM/PLE + EspPΔ5'
Darobactin : PL
1:1
10:1
100:1
c
BAM/PLE
TAM/PLE
Linear darobactin : PL
0 100
0 100
α-OmpA-C
50
30
(kDa)
Unfolded OmpA
Folded OmpA
α-EspP-C
30
(kDa)
EspPΔ5'
β barrel

Supplementary Fig. 10. Experiments related to Fig. 6. a The Western blots from two repetitions of the *in vitro* folding assays depicted in Fig. 6c are shown. **b** The effect of darobactin on the assembly of OmpA and EspPΔ5' into TAM/PLE and BAM/PLE proteoliposomes (PL) was analyzed in three independent experiments (the effect of darobactin on EspPΔ5' assembly into BAM/PLE PL was also analyzed in a fourth independent experiment at ratios of 1:1 and 100:1). The percent of each protein that was folded at the indicated darobactin:PL ratio was determined as described in the legend to Fig. 4. Values were then normalized to the percent folding observed in the control (i.e., in the absence of darobactin) which was defined as 100%. Error bars represent the standard error of the mean. **c** As a control to the darobactin assay shown in Fig. 6e, 2 μM BAM/PLE or TAM/PLE were incubated with no peptide or a linear peptide that has the same sequence as darobactin (WNWSKSF) at a 1:100 ratio at 30° C for 5 min[16]. Urea-denatured OmpA or EspPΔ5' was added to the reaction and incubated at 30° C for another 15 min. OMP folding was assessed by Western blot using appropriate antisera. The experiment shown in **c** was performed twice with similar results.

Supplementary Table 1. The Root Mean Square Deviation (RMSD) values of the structural superimposition of TamA (PDB: 4C00[12]) and BamA (PDG: 8BVQ[13]) β-barrels. The amino acid residues that were considered in each analysis are listed below.

TamA to BamA	TamA residues	BamA residues	RMSD (including sidechains)	RMSD (only backbone)
β barrel	266-577	424-807	4.566 Å	4.076 Å
β barrel with no extracellular loops	266-274,280-303,309-338,342-375,380-415,427-454,499-525,536-558,568-577	424-432,438-461,467-494,505-536,564-599,608-639,711-744,767-788,803-807	3.730 Å	3.049 Å
Only the β-strands	268-274,280-288,298-303,309-317,327-338,342-355,362-375,380-397,406-415,427-438,444-454,499-510,515-525,536-545,549-558,568-571	426-432,438-445,456-461,467-474,485-494,505-519,524-536,564-578,591-599,608-619,628-639,711-719,734-744,767-777,782-788,803-805	2.722 Å	2.078 Å

Supplementary Table 2. Proteins analyzed in Fig. S9. The BamA and TamA orthologs **a**, and the TpsB proteins **b** analyzed in Supplementary Fig. 8 and their taxonomic classifications are listed below. The sequence identities are based on the alignment of the indicated protein to the corresponding protein in *E. coli*. Protein sequences were obtained from the UniProtKB database[17,18].

a Proteobacterial BamA and TamA proteins

#	Species	Taxon ID	Proteobacteria Class	Order	Family	BamA	Identity	TamA	Identity
1	*Escherichia coli* K-12	83333	Gamma-	Enterobacterales	Enterobacteriaceae	P0A940	--	P0ADE4	--
2	*Caulobacter vibrioides*	155892	Alpha-	Caulobacterales	Caulobacteraceae	A0A290MX45	29%	A0A258D8B0	25%
3	*Paremcibacter congregatus*	2043170	Alpha-	Emcibacterales	Emcibacteraceae	A0A2G4YV76	27%	A0A2G4YQ22	25%
4	*Futiania mangrovii*	2959716	Alpha-	Futianiales	Futianiaceae	A0A9J6PIA7	27%	A0A9J6P9M1	28%
5	*Bartonella apis*	1686310	Alpha-	Hyphomicrobiales	Bartonellaceae	A0A1U9MAW3	30%	A0A1R0FBU5	26%
6	*Brucella daejeonensis*	659015	Alpha-	Hyphomicrobiales	Brucellaceae	A0A7W9AWB9	29%	A0A7W9ATT9	25%
7	*Methylocystis heyeri*	391905	Alpha-	Hyphomicrobiales	Methylocystaceae	A0A6B8KFH3	26%	A0A6B8KM79	25%
8	*Rhizobium radiobacter*	358	Alpha-	Hyphomicrobiales	Rhizobiaceae	A0A083ZRT7	27%	A0A2L2LES7	28%
9	*Rhizobium tibeticum*	501024	Alpha-	Hyphomicrobiales	Rhizobiaceae	A0A1H8MCJ0	29%	A0A1K0J5A2	27%
10	*Hyphomonadaceae bacterium*	2026748	Alpha-	Hyphomonadales	Hyphomonadaceae	A0A3D2W4F5	28%	A0A7Y1SMQ3	35%
11	*Parvularcula mediterranea*	2732508	Alpha-	Parvularculales	Parvularculaceae	A0A7Y3RM99	32%	A0A7Y3W469	25%
12	*Paracoccaceae bacterium*	1904441	Alpha-	Rhodobacterales	Paracoccaceae	A0A848XHL4	26%	A0A5P3A0E1	31%
13	*Azospirillum sp.* OGB3	2587012	Alpha-	Rhodospirillales	Azospirillaceae	A0A839W013	28%	A0A839VW41	36%
14	*Sphingomonadales bacterium*	1978525	Alpha-	Sphingomonadales	unclassified	A0A4Q3D7X6	25%	A0A838MRH5	25%
15	*Burkholderia cenocepacia*	95486	Beta-	Burkholderiales	Burkholderiaceae	A0A144T9J8	37%	A0A7G6V1N6	27%
16	*Leptothrix mobilis*	47994	Beta-	Burkholderiales	Sphaerotilaceae	A0A4Q7LVB8	33%	A0A4Q7LCZ4	29%
17	*Neisseria meningitidis*	487	Beta-	Neisseriales	Neisseriaceae	E6MUY7	33%	E6MZX1	24%
18	*Methylophilaceae bacterium*	2030816	Beta-	Nitrosomonadales	Methylophilaceae	A0A4P5TUP1	38%	A0A849TAH7	27%
19	*Rhodocyclaceae bacterium*	1898103	Beta-	Rhodocyclales	Rhodocyclaceae	A0A7V8C181	37%	A0A838LUG7	25%
20	*Aeromonas veronii*	654	Gamma-	Aeromonadales	Aeromonadaceae	A0A494U3C2	52%	A0A6I6HTE2	40%

21	*Hydrocarboniclastica marina*	2259620	Gamma-	Alteromonadales	Alteromonadaceae	A0A4P7XIS3	38%	A0A4V1D8C0	32%
22	*Ignatzschineria indica*	472583	Gamma-	Cardiobacteriales	Ignatzschineriaceae	A0A2U2ANM4	32%	A0A2U2AN98	27%
23	*Microbulbifer sp.* YPW1	2745199	Gamma-	Cellvibrionales	Microbulbiferaceae	A0A7H8Q2C5	38%	A0A7H8PZ97	25%
24	*Halothiobacillaceae bacterium*	2268194	Gamma-	Chromatiales	Halothiobacillaceae	A0A545SJF3	38%	A0A545SGY5	30%
25	*Legionella parisiensis*	45071	Gamma-	Legionellales	Legionellaceae	A0A1E5JLE2	33%	A0A1E5JL08	25%
26	*Methylococcaceae bacterium*	1933926	Gamma-	Methylococcales	Methylococcaceae	A0A7Z9PN15	37%	A0A849S5M6	31%
27	*Acinetobacter seifertii*	1530123	Gamma-	Moraxellales	Moraxellaceae	A0A7H2PVL2	35%	A0A7H2ZTW7	35%
28	*Steroidobacteraceae bacterium*	2689616	Gamma-	Nevskiales	Steroidobacteraceae	A0A925Q7G3	32%	A0A925T0A7	30%
29	*Halomonas endophytica*	2033802	Gamma-	Oceanospirillales	Halomonadaceae	A0A2N7U768	38%	A0A2N7U5D0	35%
30	*Haemophilus influenzae*	71421	Gamma-	Pasteurellales	Pasteurellaceae	P44935	45%	P44038	43%
31	*Pseudomonas fluorescens*	294	Gamma-	Pseudomonadales	Pseudomonadaceae	A0A5E6VCR2	38%	A0A5E6MV42	34%
32	*Salinisphaera shabanensis* E1L3A	1033802	Gamma-	Salinisphaerales	Salinisphaeraceae	U2EQ60	34%	U2FWP7	30%
33	*Methylophaga aminisulfidivorans*	230105	Gamma-	Thiotrichales	Piscirickettsiaceae	A0A7C1VQI6, A0A7C1ZSZ7[a]	36%	A0A7C1ZUK5	31%
34	*Vibrio casei*	673372	Gamma-	Vibrionales	Vibrionaceae	A0A368LLR9	56%	A0A368LQ78	46%
35	*Luteibacter rhizovicinus*	242606	Gamma-	Xanthomonadales	Rhodanobacteraceae	A0A4R3YIF2	35%	A0A4R3YPA5	30%
36	*Candidatus Sedimenticola endophacoides*	2548426	Gamma-	Gammaproteobacteria incertae sedis	Sedimenticola	A0A657PPR4	38%	A0A6N4DT79	30%
37	*Solidesulfovibrio magneticus*	573370	Delta-	Desulfovibrionaceae	Desulfovibrionales	C4XN42	26%	C4XLA3	27%
38	*Geobacteraceae bacterium* GWC2_58_44	1798318	Delta-	Desulfuromonadia	Geobacterales	A0A1G0MJ53	26%	A0A1G0M6T3	29%
39	*Campylobacterales bacterium*	2268180	Epsilon-	Campylobacterales	unclassified Campylobacterales	A0A7V4QAC1	25%	A0A7C5NR37	21%

b Proteobacterial TpsB proteins

#	Species	Taxon ID	Proteobacteria Class	Order	Family	TpsB	Identity
1	*Escherichia coli* O104:H21[b]	1335302	Gamma-	Enterobacterales	Enterobacteriaceae	A0A0F6YRV6	--
2	*Caulobacter vibrioides* ATCC 19089[b]	190650	Alpha-	Caulobacterales	Caulobacteraceae	Q9AAJ4	23%
3	*Paremcibacter congregatus*	2043170	Alpha-	Emcibacterales	Emcibacteraceae	A0A2G4YPM2	0%
4	[c]	--	Alpha-	Futianiales	--	--	
5	*Bartonella apis*	1686310	Alpha-	Hyphomicrobiales	Bartonellaceae	A0A1U9MJ53	29%
6	*Brucella rhizosphaerae*[b]	571254	Alpha-	Hyphomicrobiales	Brucellaceae	A0A256FQ02	29%
7	*Methylocystis bryophila*[b]	655015	Alpha-	Hyphomicrobiales	Methylocystaceae	A0A1W6MQG9	26%
8	*Rhizobium radiobacter*	358	Alpha-	Hyphomicrobiales	Rhizobiaceae	A0A176XHK7	25%
9	*Rhizobium sp.* CCGE532[b]	2364272	Alpha-	Hyphomicrobiales	Rhizobiaceae	A0A387H4B0	30%
10	*Hyphomonas sp.* 32-62-5[b]	1970391	Alpha-	Hyphomonadales	Hyphomonadaceae	A0A258C1M9	0%
11	*Parvularcula mediterranea*	2732508	Alpha-	Parvularculales	Parvularculaceae	A0A7Y3RJ97	0%
12	*Paracoccaceae bacterium*	1904441	Alpha-	Rhodobacterales	Paracoccaceae	A0A973WGL4	24%
13	*Azospirillum sp.* OGB3	2587012	Alpha-	Rhodospirillales	Azospirillaceae	A0A839W3Y0	21%
14	*Sphingomonadales bacterium*	1978525	Alpha-	Sphingomonadales	unclassified	A0A838MLD1	20%
15	*Burkholderia cenocepacia*	95486	Beta-	Burkholderiales	Burkholderiaceae	A0A088V661	28%
16	*Roseateles sp.* SL47[b]	2995138	Beta-	Burkholderiales	Sphaerotilaceae	A0A9E8UHA9	24%
17	*Neisseria meningitidis*	487	Beta-	Neisseriales	Neisseriaceae	E6MYC8	29%
18	*Methylophilaceae bacterium*	2030816	Beta-	Nitrosomonadales	Methylophilaceae	A0A849SK58	26%
19	*Rhodocyclaceae bacterium*	1898103	Beta-	Rhodocyclales	Rhodocyclaceae	A0A838LYK9	28%
20	*Aeromonas veronii*	654	Gamma-	Aeromonadales	Aeromonadaceae	A0A833NCR1	41%
21	*Hydrocarboniclastica marina*	2259620	Gamma-	Alteromonadales	Alteromonadaceae	A0A4P7XFU7	24%
22	*Cardiobacterium sp.*[b]	2382124	Gamma-	Cardiobacteriales	Cardiobacteriaceae	A0A660MQD1	26%
23	*Microbulbifer sp.* YPW1	2745199	Gamma-	Cellvibrionales	Microbulbiferaceae	A0A7H8PVN6	22%
24	*Halothiobacillaceae bacterium*	2268194	Gamma-	Chromatiales	Halothiobacillaceae	A0A831ULR8	28%

25	*Coxiella sp.* (in: g-proteobacteria)[b]	59288	Gamma-	Legionellales	Coxiellaceae	A0A2G2A272	23%
26	*Methylococcaceae bacterium*	1933926	Gamma-	Methylococcales	Methylococcaceae	A0A849UB01	22%
27	*Acinetobacter seifertii*	1530123	Gamma-	Moraxellales	Moraxellaceae	A0A7H2WKL7	26%
28	*Steroidobacteraceae bacterium*	2689616	Gamma-	Nevskiales	Steroidobacteraceae	A0A925T2M0	23%
29	*Halomonas endophytica*	2033802	Gamma-	Oceanospirillales	Halomonadaceae	A0A2N7U1P8	28%
30	*Haemophilus influenzae*	71421	Gamma-	Pasteurellales	Pasteurellaceae	P44601	18%
31	*Pseudomonas fluorescens*	294	Gamma-	Pseudomonadales	Pseudomonadaceae	A0A5E7GVN4	32%
32	[c]	--	Gamma-	Salinisphaerales	--	--	
33	*Methylophaga aminisulfidivorans*	230105	Gamma-	Thiotrichales	Piscirickettsiaceae	A0A7C1VSZ8	29%
34	*Vibrio casei*	673372	Gamma-	Vibrionales	Vibrionaceae	A0A368LFX4	22%
35	*Luteibacter rhizovicinus*	242606	Gamma-	Xanthomonadales	Rhodanobacteraceae	A0A4R3YR19	32%
36	*Candidatus Sedimenticola endophacoides*	2548426	Gamma-	Gammaproteobacteria incertae sedis	Sedimenticola	A0A6N4E546	23%
37	*Desulfovibrio ferrophilus*[b]	241368	Delta-	Desulfovibrionaceae	Desulfovibrionaceae	A0A2Z6AZ57	32%
38	*Geobacteraceae bacterium* GWC2_58_44	1798318	Delta-	Desulfuromonadia	Geobacterales	A0A1G0MME5	22%
39	*Campylobacteraceae bacterium*[b]	2268179	Epsilon-	Campylobacterales	Campylobacteraceae	A0A850K0B8	23%

[a] *Methylophaga aminisulfidivorans* BamA is split into two segments in the UniprotKB database.

[b] Because there are no species in this Proteobacterial family that has BamA, TamA and TpsB proteins annotated in the UniprotKB database, a TpsB protein identified in a different species from the same family was used.

[c] There are no TpsB proteins annotated in this Proteobacterial family in the UniprotKB database.

Supplementary Table 3. Plasmids used in this study.

Plasmid	Description	Reference
pYG120	pTrc99a::*bamABCDE*$_{8His}$-*bamB*	[19]
pXW03	pET28b::*ompA*$_{22\text{-}346}$	[20]
pXW14	pET28b::$ompA_{22-346}^{Y189A,F191A}$	[20]
pXW15	pET28b::$ompA_{22-346}^{V98R,L100R}$	[20]
pET28b::*espPΔ5'*		[21]
pXW21	pET28b::*espPΔ5'*^{Y1298A,F1300A}	[20]
pSH01	pET28b::*espPΔ5'*^{I1119R}	[20]
pET21::*ag43*$_{552\text{-}1039}$		[22]
pET303::*fadL*$_{26\text{-}446}$		Provided by Joanna Slusky (University of Kansas)
pXW47	pTrc99a:*(His$_8$)tamAB*	This study
pXW48	pTrc99a:*(His$_8$)tamAB-tamB*	This study
pXW49	pTrc99a:*(His$_8$)tamA*	This study
pXW50	pTrc99a:*(His$_8$)tamA*$^{G271C,\ G574C}$*tamB-tamB*	This study

Supplementary Table 4. Oligonucleotides and gBlocks used in this study.

DNA	Sequence	Notes
XW03	TGTTTTTTGCGCCGACATCA	F, sequencing primer for pTrc99A[20]
XW04	TGGGACCACCGCGCTACT	R, sequencing primer for pTrc99A[20]
XW63	CCAGGGGCAGGAAAAAAGGATATTC	F, primer to clone *tamAB* with 35 upstream base pairs (*tamAB*$_{-35-5510}$)
XW64	CTAAAACTCGAACTGATAGAGCAAA	R, primer to clone *tamAB* with 35 upstream base pairs (*tamAB*$_{-35-5510}$)
XW65	AGCGGATAACAATTTCACACAGGAAACAGACCATGG CCAGGGGCAGGAAAAAAGGA	F, primer to clone *tamAB*$_{-35-66}$ and assemble it with pTrc
XW66	GCCGCCGCTGCCGCCGCTGCCGCCATGGTGGTGGTGATGGTGGTGGTGCGCGGCG ACGGCAGATCCGCT	R, primer to clone *tamAB*$_{-35-66}$ and assemble it with an octa-histidine tag (-HHHHHHHHGGSGGSGG-) at its 3' end
XW67	CACCATGGCGGCAGCGGCGGCAGCGGCGGCAACGTCCGTCTACAGGTCGAGGGGT	F, primer to clone *tamAB*$_{67-5510}$ or *tamA*$_{67-1734}$ and assemble it with an octa-histidine tag (-HHHHHHHHGGSGGSGG-) at its 5' end
XW68	AGCTTGCATGCCTGCAGGTCGACTCTAGAGGATCCCTAAAACTCGAACTGATAGAGC AAA	R, primer to clone *tamAB*$_{67-5510}$ and assemble it with pTrc
XW69	AGCTTGCATGCCTGCAGGTCGACTCTAGAGGATCCTCATAATTCTGGCCCCAGACCG ATG	R, primer to clone *tamA*$_{67-1734}$ and assemble it with pTrc
XW70	ATGAGTTTATGGAAAAAAATCAGCCTCG	F, primer to clone *tamB*
XW71	CTAAAACTCGAACTGATAGAGCAAATCCAG	R, primer to clone *tamB*
XW72	TAAGCTATATCTGGAAGCCGTGTCTGGTGTAGACCAGGCACTGGATTTGCTCTATCAG TTCGAGTTTTAGGGATCCTCTAGATGATAACGAGGCGCAAAAAATGAGTTTATGGAAAA AAATCAGCCTCGGCGTGGTTATCGTTATCTTACTGTTGCTGGGATCGGTGGCGT	gBlock to assemble an ribosome binding site and the second copy of *tamB* to pXW47
XW73	TAAGCTATATCTGGAAGCCGTGTCTGGTGTAGACCAGGCACTGGATTTGCTCTATCAG TTCGAGTTTTAGCTAGAGTCGACCTGCAGGCATGCAAGCTTGGCTGTTTTGGCGGATG AGAGAAGATTTTCAGCCTGATACAG	gBlock to assemble the second copy of *tamB* to pXW47
XW74	GGGCGTGGTTTCGCCGCGAACA	F, sequencing primer for *tamA*
XW75	ACCGCAGTATTCGTGGCTA	F, sequencing primer for *tamA*
XW76	TAGCCACGAATACTGCGGT	R, sequencing primer for *tamA*
XW77	GCTGAAAGTGAGCAGCATTGA	F, sequencing primer for *tamB*
XW78	ACCGGTAACGTTAAACAGAAC	F, sequencing primer for *tamB*
XW79	TCAGGCCAGCGGTATCTTCACG	F, sequencing primer for *tamB*
XW80	TCCGCCACTTTCGGCAGAGC	R, sequencing primer for *tamAB*
XW81	CACCATCGAAACCTGTGTCGGTTACTCT	F, mutagenesis primer for *tamA* G271C
XW82	AGAGTAACCGACACAGGTTTCGATGGTG	R, mutagenesis primer for *tamA* G271C
XW83	TACATCGGTCTGTGCCCAGAATTATGA	F, mutagenesis primer for *tamA* G574C
XW84	TCATAATTCTGGGCACAGACCGATGTA	R, mutagenesis primer for *tamA* G574C

SUPPLEMENTARY REFERENCES

1. Hussain, S. & Bernstein, H. D. The Bam complex catalyzes efficient insertion of bacterial outer membrane proteins into membrane vesicles of variable lipid composition. *J. Biol. Chem.* **293**, 2959–2973 (2018).
2. Arora, A., Abildgaard, F., Bushweller, J. H. & Tamm, L. K. Structure of outer membrane protein A transmembrane domain by NMR spectroscopy. *Nat. Struct. Biol.* **8**, 334–338 (2001).
3. Ishida, H., Garcia-Herrero, A. & Vogel, H. J. The periplasmic domain of *Escherichia coli* outer membrane protein A can undergo a localized temperature dependent structural transition. *Biochim. Biophys. Acta (BBA) - Biomembranes* **1838**, 3014–3024 (2014).
4. Barnard, T. J. et al. Molecular basis for the activation of a catalytic asparagine residue in a self-cleaving bacterial autotransporter. *J. Mol. Biol.* **415**, 128–142 (2012).
5. Jumper, J. et al. Highly accurate protein structure prediction with AlphaFold. *Nature* 596, 583–589 (2021).
6. Varadi, M. et al. AlphaFold Protein Structure Database: massively expanding the structural coverage of protein-sequence space with high-accuracy models. *Nucleic Acids Res.* **50**, D439–D444 (2022).
7. van den Berg, B., Black, P. N., Clemons, W. M. & Rapoport, T. A. Crystal structure of the long-chain fatty acid transporter FadL. *Science* **304**, 1506–1509 (2004).
8. Reynolds, C. R., Islam, S. A. & Sternberg, M. J. E. EzMol: A web server wizard for the rapid visualization and image production of protein and nucleic acid structures. *J. Mol. Biol.* **430**, 2244–2248 (2018).
9. Dautin, N., Barnard, T. J., Anderson, D. E. & Bernstein, H. D. Cleavage of a bacterial autotransporter by an evolutionarily convergent autocatalytic mechanism. *EMBO J.* **26**, 1942–1952 (2007).
10. Charbonneau, M.-È., Janvore, J. & Mourez, M. Autoprocessing of the *Escherichia coli* AIDA-I Autotransporter. *J. Biol. Chem.* **284**, 17340–17351 (2009).
11. Hussain, S., Peterson, J. H. & Bernstein, H. D. Bam complex-mediated assembly of bacterial outer membrane proteins synthesized in an in vitro translation system. *Sci. Rep.* 10, 4557 (2020).
12. Gruss, F. et al. The structural basis of autotransporter translocation by TamA. *Nat. Struct. Mol. Biol.* **20**, 1318–1320 (2013).
13. Haysom, S. F. et al. Darobactin B stabilises a lateral-closed conformation of the BAM complex in *E. coli* Cells. *Angew. Chem. International Edition* **62**, e202218783 (2023).
14. Papadopoulos, J. S. & Agarwala, R. COBALT: constraint-based alignment tool for multiple protein sequences. *Bioinformatics* **23**, 1073–1079 (2007).
15. Crooks, G. E., Hon, G., Chandonia, J.-M. & Brenner, S. E. WebLogo: a sequence logo generator. *Genome Res.* **14**, 1188–1190 (2004).
16. Kaur, H. et al. The antibiotic darobactin mimics a β-strand to inhibit outer membrane insertase. *Nature* **593**, 125–129 (2021).
17. The UniProt Consortium. UniProt: the Universal Protein Knowledgebase in 2023. *Nucleic Acids Res.* **51**, D523–D531 (2023).
18. O'Leary, N. A. et al. Reference sequence (RefSeq) database at NCBI: current status, taxonomic expansion, and functional annotation. *Nucleic Acids Res.* **44**, D733–D745 (2016).
19. Gu, Y. et al. Structural basis of outer membrane protein insertion by the BAM complex. *Nature* **531**, 64–69 (2016).
20. Wang, X., Peterson, J. H. & Bernstein, H. D. Bacterial outer membrane proteins Are targeted to the Bam complex by two parallel mechanisms. *mBio* **12**, e00597-21 (2021).
21. Roman-Hernandez, G., Peterson, J. H. & Bernstein, H. D. Reconstitution of bacterial autotransporter assembly using purified components. *eLife* **3**, e04234 (2014).

22. Yan, Z., Hussain, S., Wang, X., Bernstein, H. D. & Bardwell, J. C. A. Chaperone OsmY facilitates the biogenesis of a major family of autotransporters. *Mol. Microbiol.* **112**, 1373–1387 (2019).
