# Supplementary material for: The translocation assembly module (TAM) catalyzes the assembly of bacterial outer membrane proteins in vitro
Source: Nat Commun. 2024 Aug 23;15:7246. doi: 10.1038/s41467-024-51628-8 (PMC11341756; doi:10.1038/s41467-024-51628-8)
Supplement: Supplementary file 4 — Source Data [file 41467_2024_51628_MOESM4_ESM.zip › Source data2.pdf]

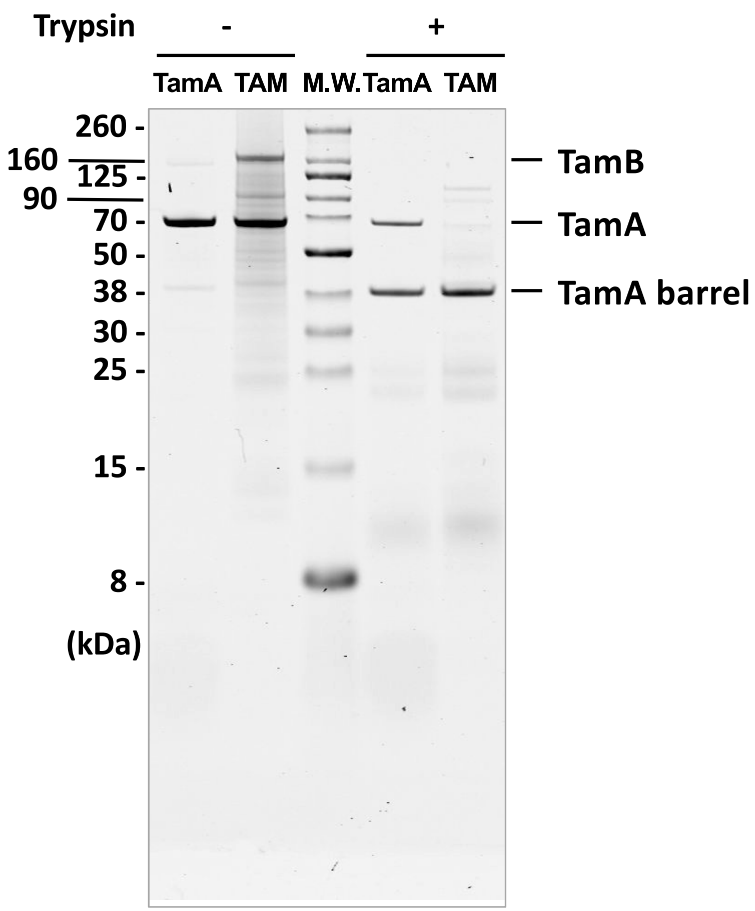


**Related to Fig. 1b. Trypsin digest of TamA/PLE and TAM/PLE. Products were resolved on a 12% NuPAGE Tris-Bis gel (Thermo Fisher) run with MES buffer and visualized by Coomassie blue staining. 20 times as much protein was loaded onto the gel as in Fig. 2c.**

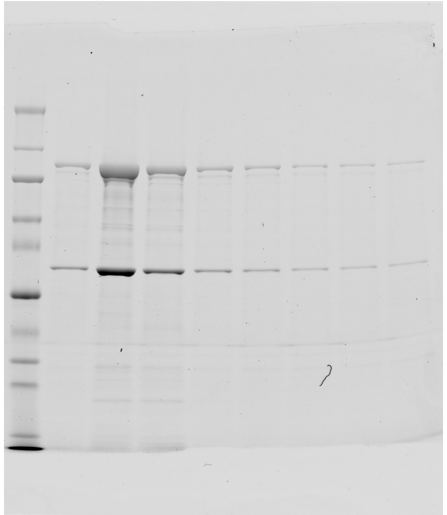

**Fig. 2a, Coomassie blue staining**

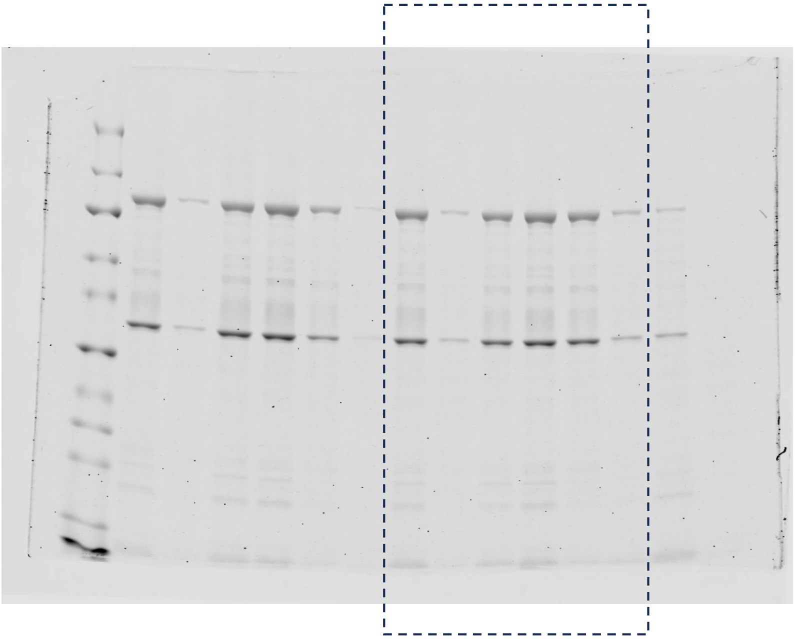

**Fig. 2b, Coomassie blue staining**

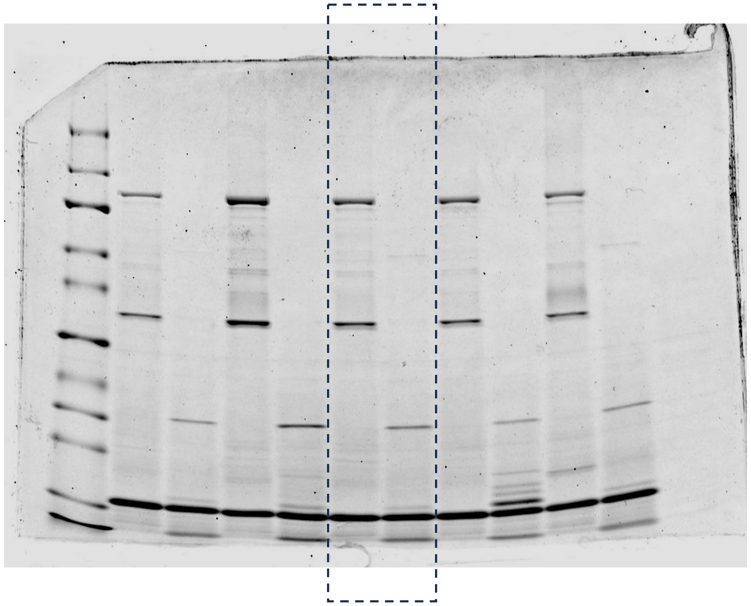

**Fig. 2c, Coomassie blue staining**

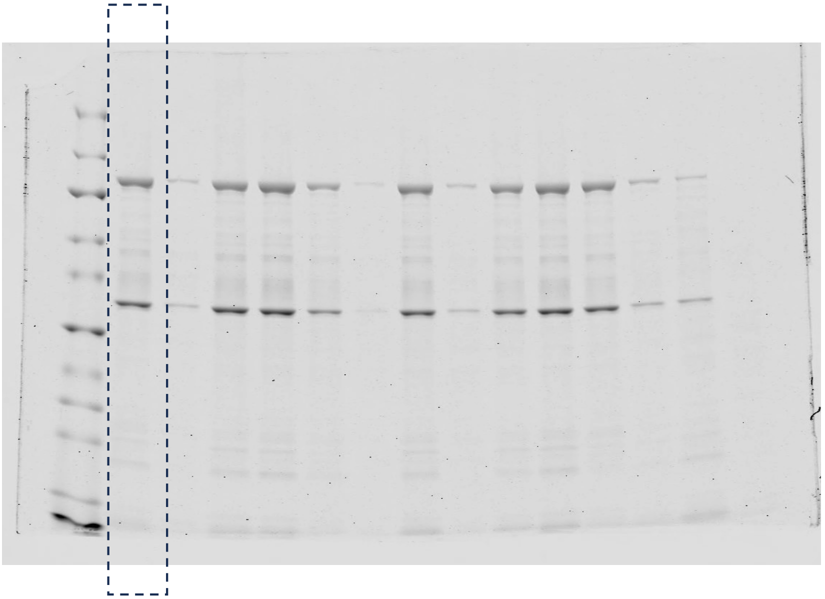

**Fig. 2d, lane 1, Coomassie blue staining**

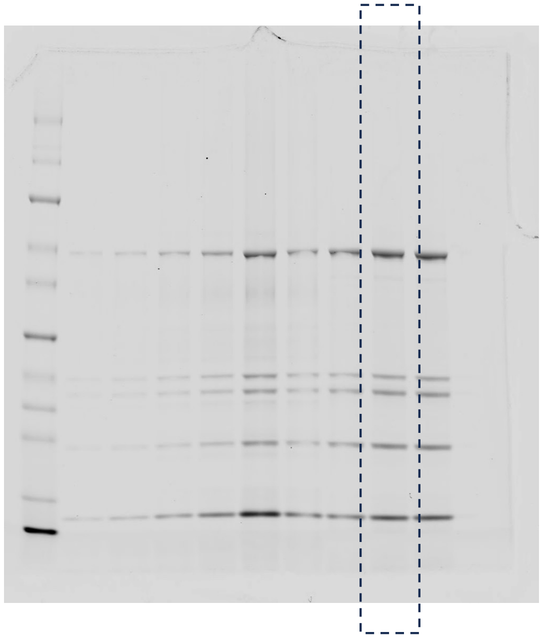

**Fig. 2d, lane 2, Coomassie blue staining**

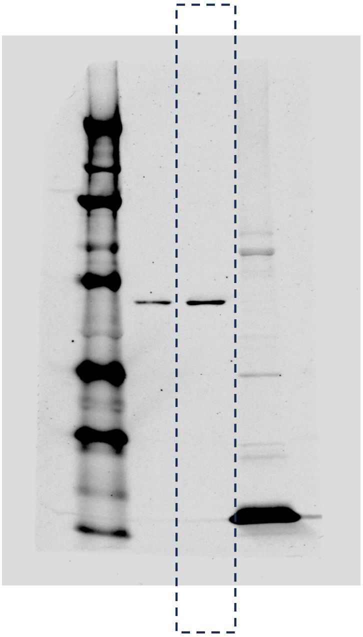

**Fig. 2d, lane 3, α-6xHis**

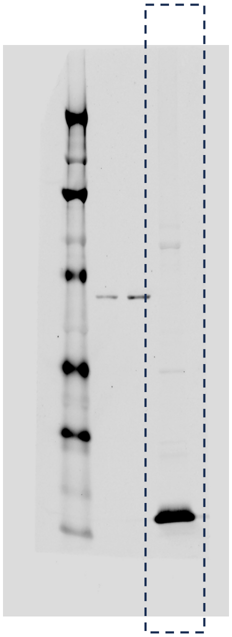

**Fig. 2d, lane 4, α-6xHis**

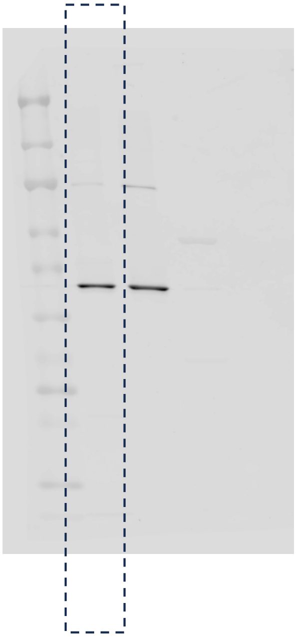

**Fig. 2d, lane 5, α-TamA-N**

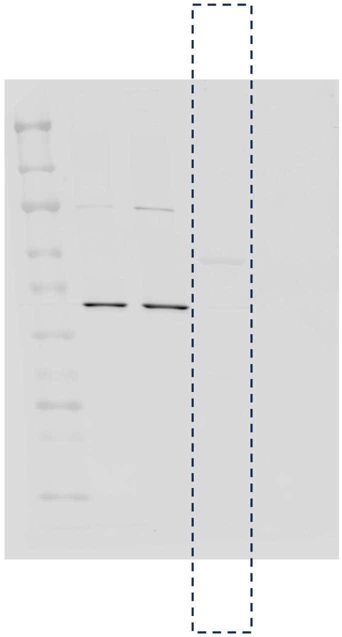

**Fig. 2d, lane 6, α-TamA-N**

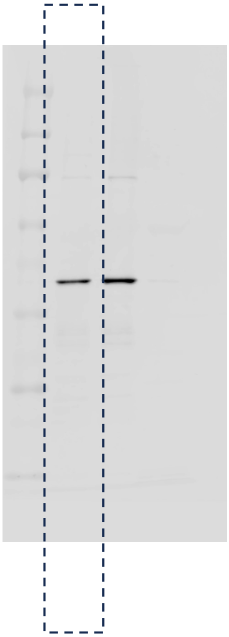

**Fig. 2d, lane 7, α-TamA-C**

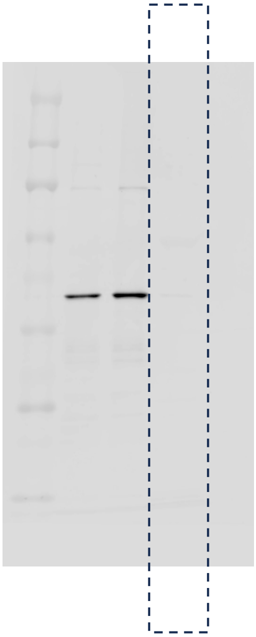

**Fig. 2d, lane 8, α-TamA-C**

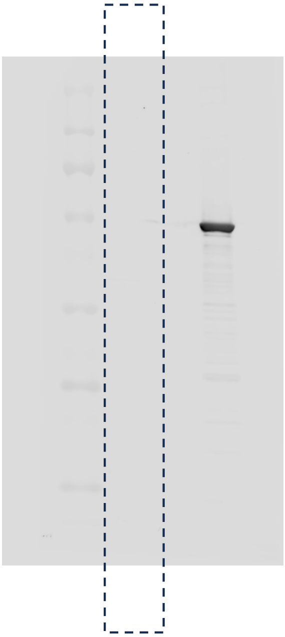

**Fig. 2d, lane 9, α-BamA-C**

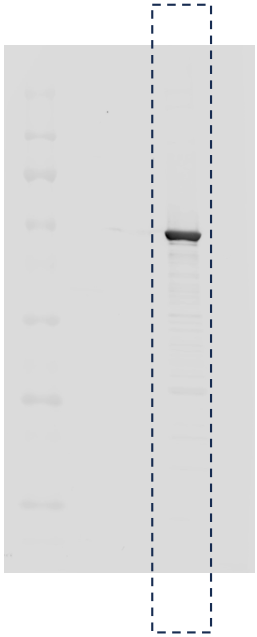

**Fig. 2d, lane 10, α-BamA-C**

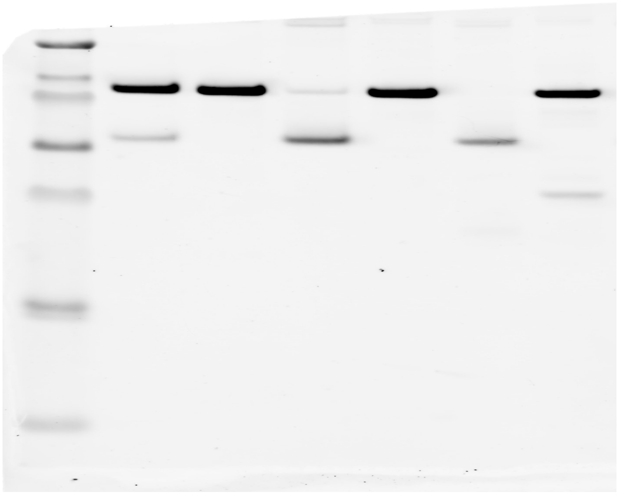

**Fig. 3a, blot-1, α-OamA-L4**

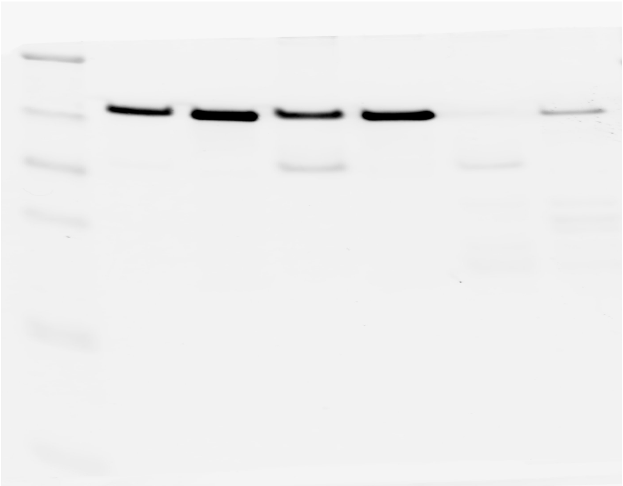

**Fig. 3a, blot-2, α-OamA-L4**

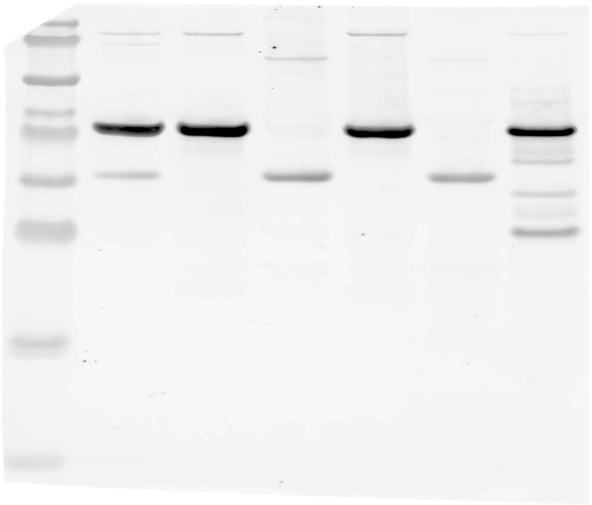

**Fig. 3a, blot-3, α-OamA-L4**

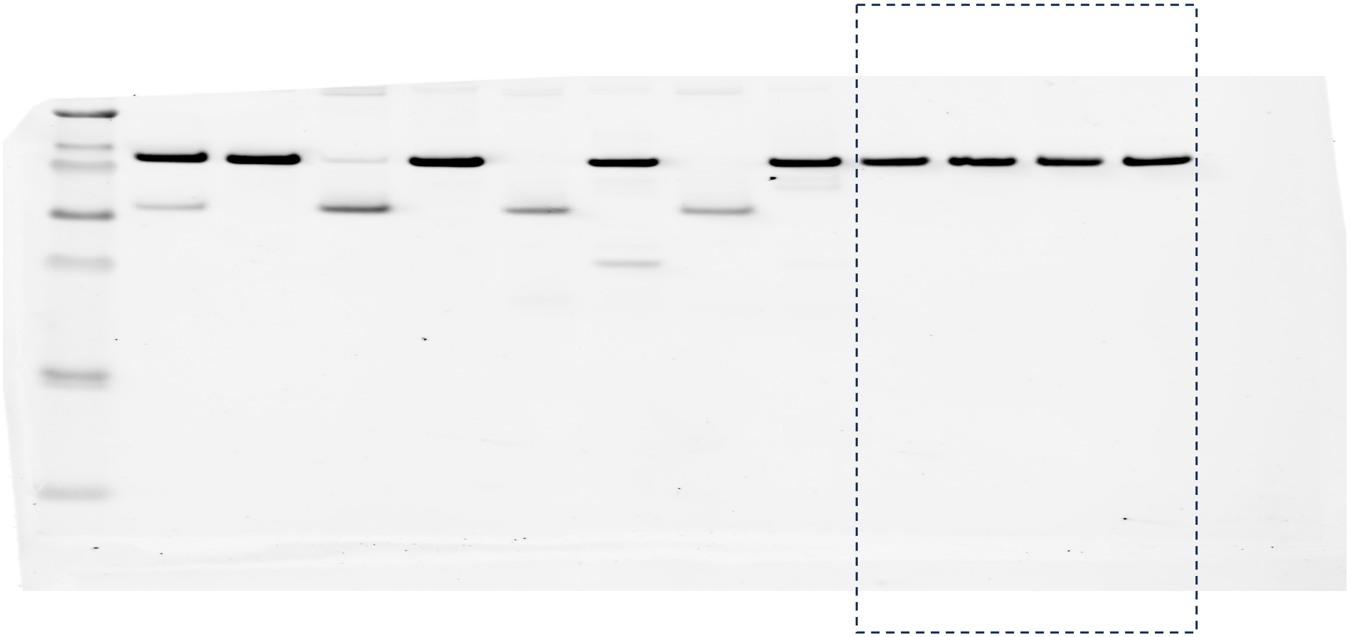

**Fig. 3a, blot-4 left, α-OamA-L4**

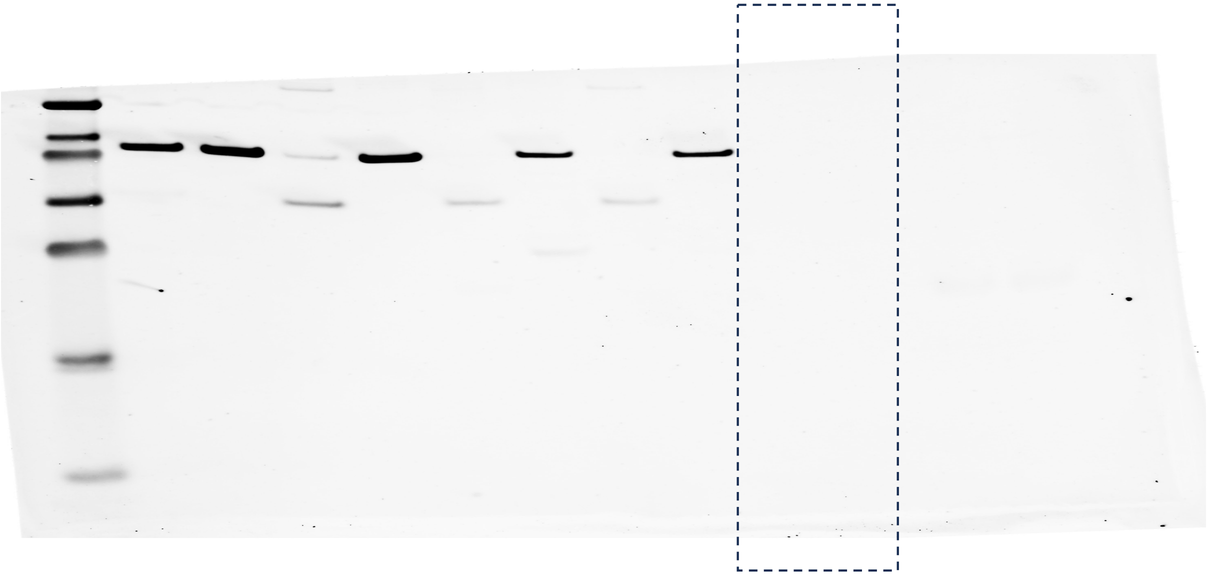

**Fig. 3a, blot-4 right, α-OamA-L4**

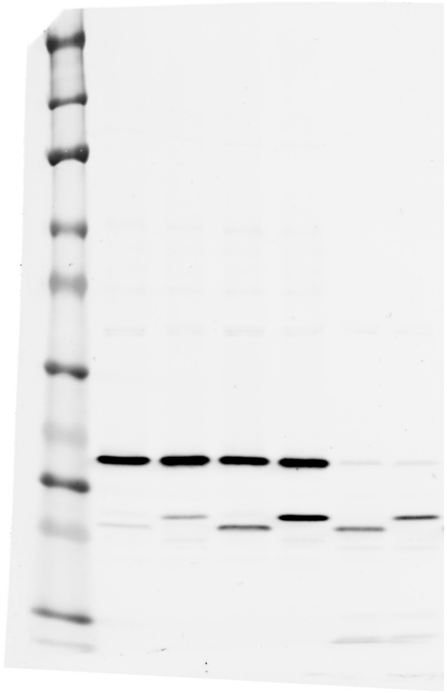

**Fig. 3b, blot-1, α-EspP-C**

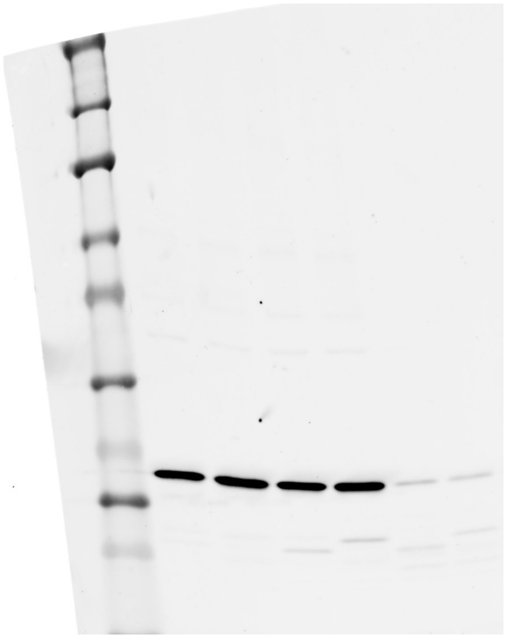

**Fig. 3b, blot-2, α-EspP-C**

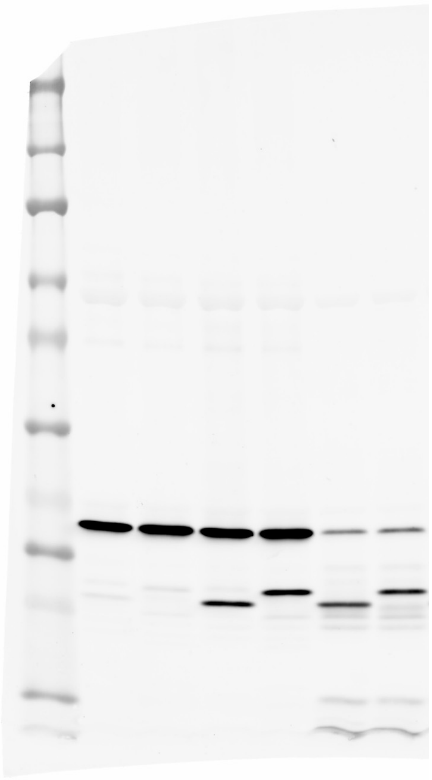

**Fig. 3b, blot-3, α-EspP-C**

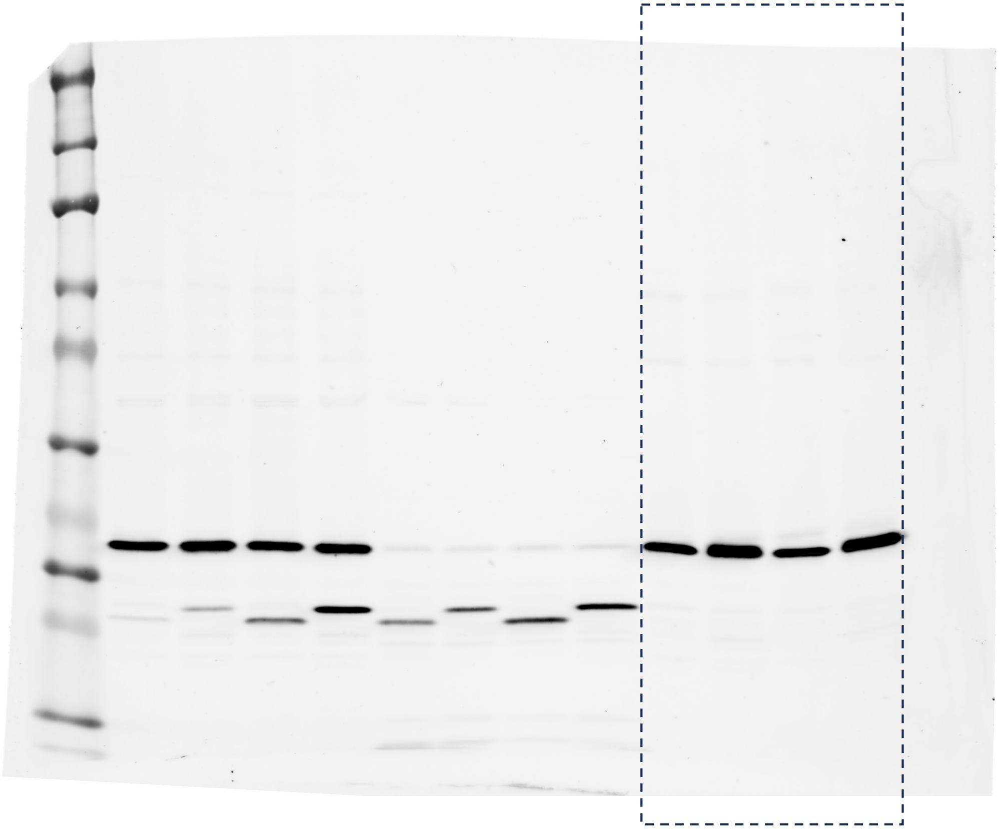

**Fig. 3b, blot-4 left, α-EspP-C**

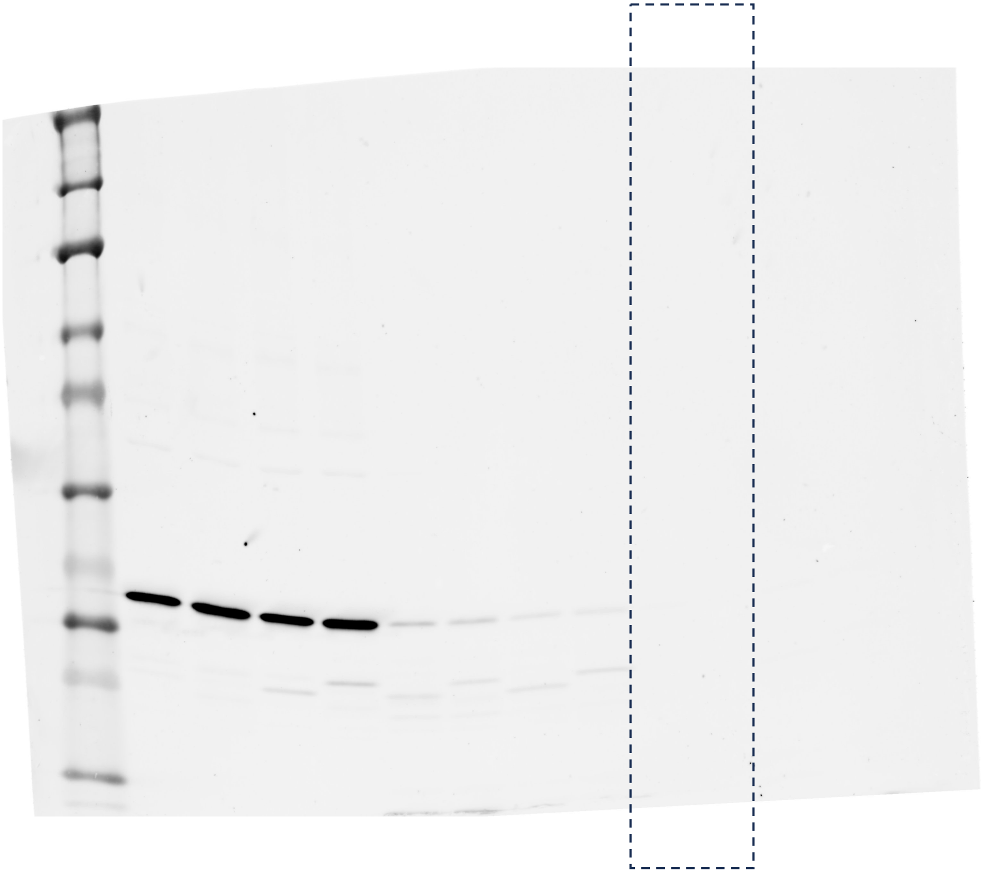

**Fig. 3b, blot-4 right, α-EspP-C**

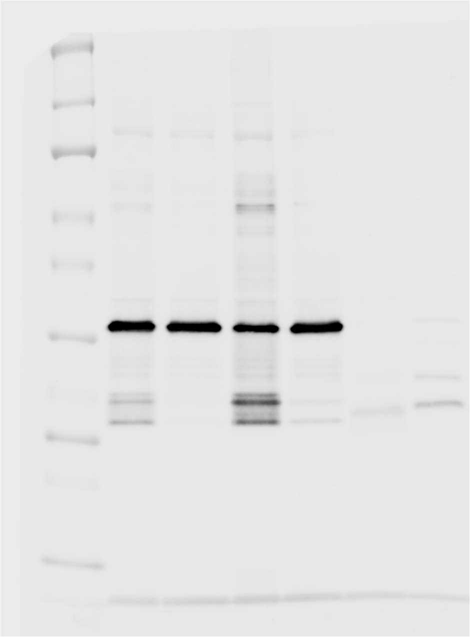

**Fig. 3c, blot-1, α-Ag43-β**

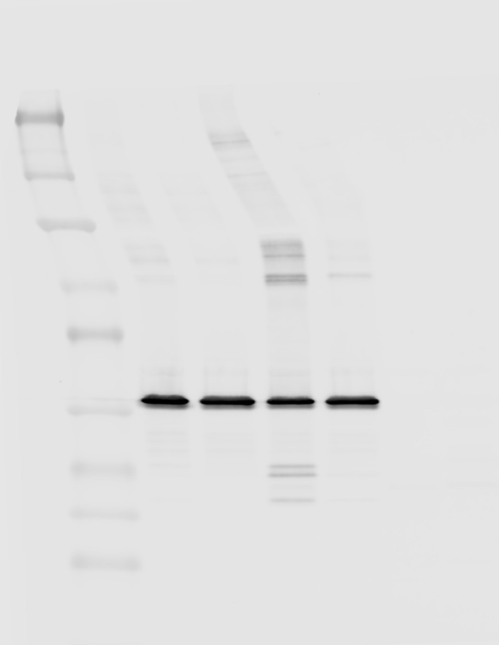

**Fig. 3c, blot-2, α-Ag43-β**

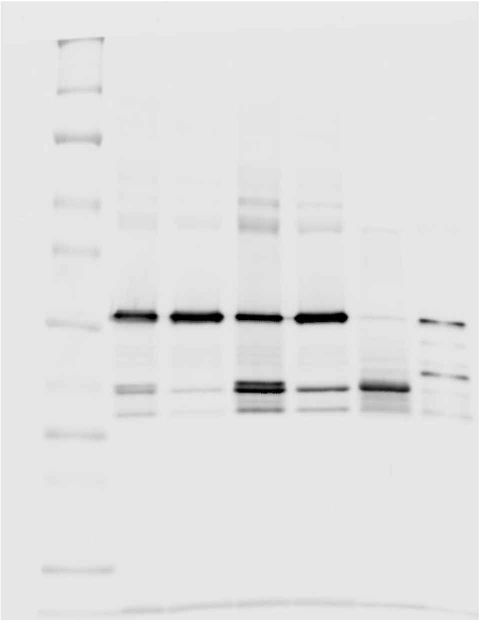

**Fig. 3c, blot-3, α-Ag43-β**

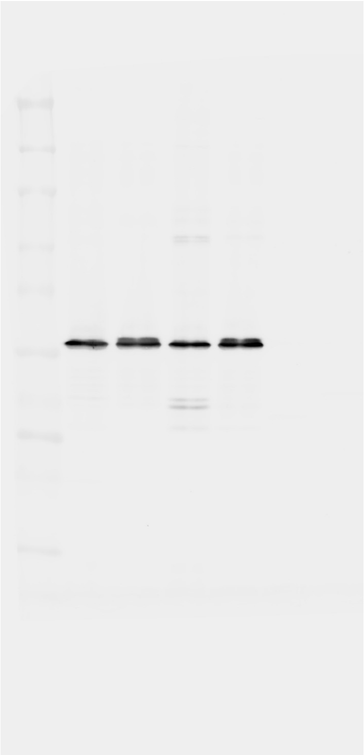

**Fig. 3c, blot-4, α-Ag43-β**

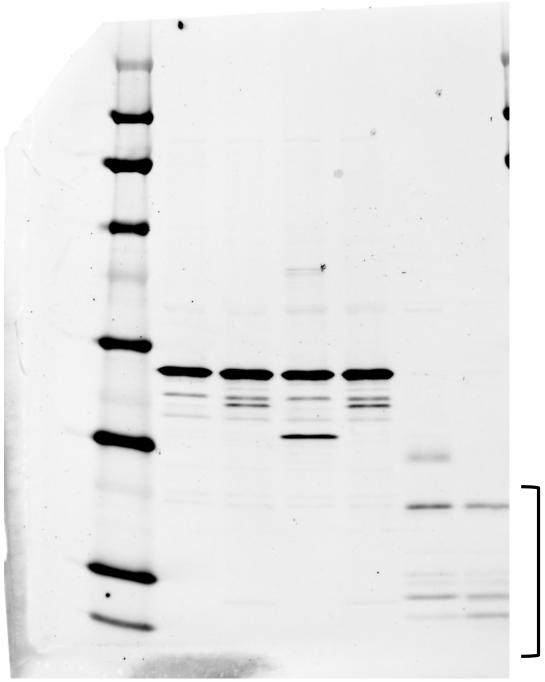


**Fig. 3d, blot-1, α-FadL-C**

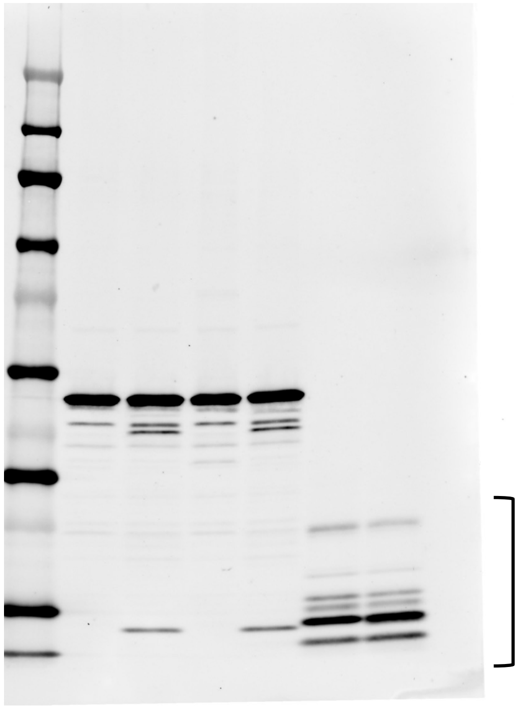


**Fig. 3d, blot-2, α-FadL-C**

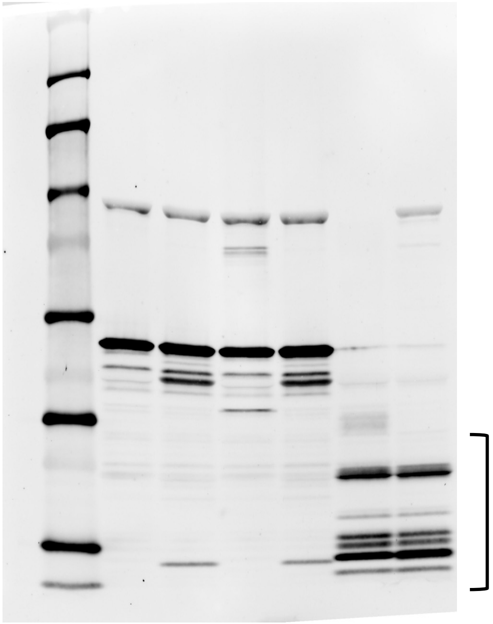


**Fig. 3d, blot-3, α-FadL-C**

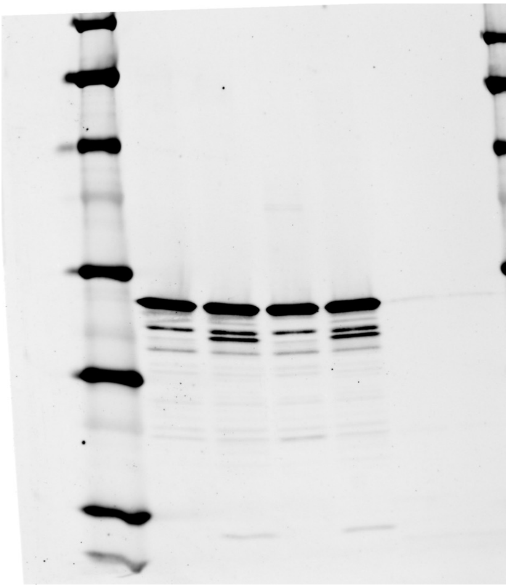

**Fig. 3d, blot-4, α-FadL-C**

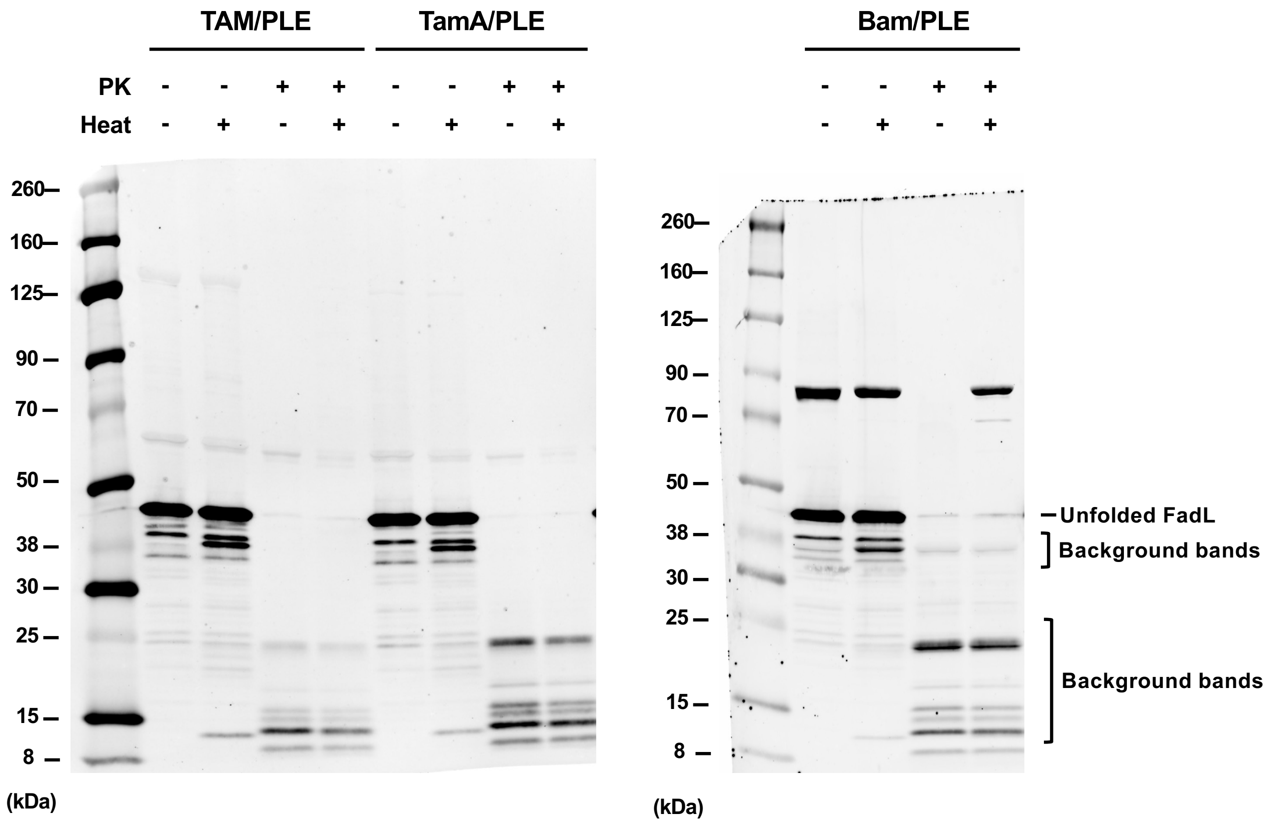


**FadL PK digestion control to show the background bands.** In the *in vitro* FadL folding assays shown in Fig. 3d, samples were collected at *t=0.5 min* and were subjected to PK digestion or left untreated. Samples were placed on ice or heated to 95°C and resolved by SDS-PAGE. The folding of FadL was visualized by Western blot using an antiserum against an FadL C-terminal peptide.

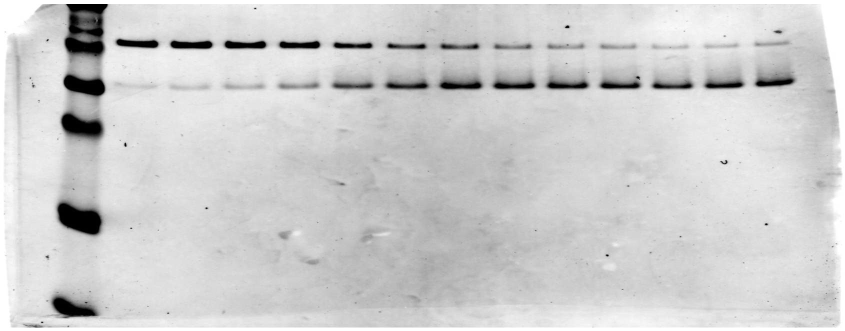

**Fig. 4, top blot, α-OmpA-C**

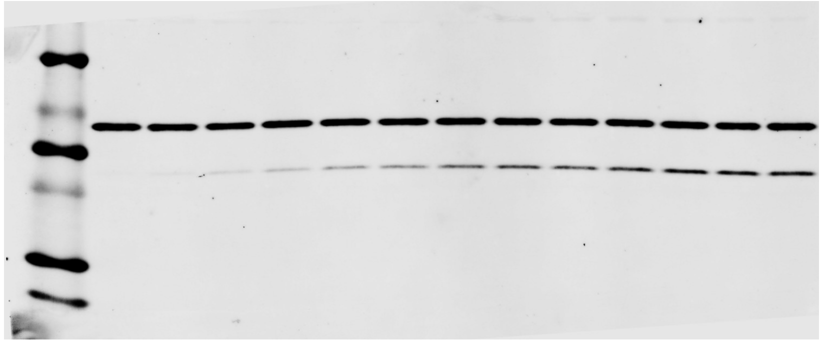

**Fig. 4, bottom blot, α-EspP-C**

| | OmpA | | | | | EspPΔ5’ | | | |
|---|---|---|---|---|---|---|---|---|---|
| Time (min) | #1 | #2 | #3 | #4 | #5 | #1 | #2 | #3 | #4 |
| 0 | 0.0472 | 0.0461 | 0.0268 | 0.0565 | 0.0600 | 0.0035 | 0 | 0 | 0 |
| 0.25 | 0.0976 | 0.1033 | 0.0536 | 0.1022 | 0.0771 | 0.0239 | 0.0069 | 0.0031 | 0.0038 |
| 0.5 | 0.1610 | 0.1008 | 0.0949 | 0.1400 | 0.1136 | 0.0509 | 0.0194 | 0.0056 | 0.0061 |
| 1 | 0.2921 | 0.2120 | 0.2183 | 0.2938 | 0.2186 | 0.0564 | 0.0397 | 0.0119 | 0.0260 |
| 2 | 0.5105 | 0.4287 | 0.4790 | 0.5297 | 0.4301 | 0.0891 | 0.0797 | 0.0362 | 0.0548 |
| 3 | 0.6391 | 0.5369 | 0.6321 | 0.6536 | 0.6400 | 0.1143 | 0.1097 | 0.0577 | 0.0764 |
| 5 | 0.7695 | 0.6923 | 0.7636 | 0.8030 | 0.7169 | 0.1378 | 0.1426 | 0.0770 | 0.0932 |
| 10 | 0.8516 | 0.8000 | 0.8804 | 0.8873 | 0.7764 | 0.1734 | 0.1567 | 0.0998 | 0.1348 |
| 15 | 0.8698 | 0.8133 | 0.9048 | 0.9268 | 0.8158 | 0.1777 | 0.1945 | 0.1177 | 0.1538 |
| 20 | 0.8754 | 0.8346 | 0.8987 | 0.9177 | 0.8362 | 0.2038 | 0.2224 | 0.1302 | 0.1758 |
| 30 | 0.9007 | 0.8577 | 0.9172 | 0.9108 | 0.8670 | 0.2480 | 0.2245 | 0.1484 | 0.1835 |
| 45 | 0.8924 | 0.8348 | 0.9251 | 0.9499 | 0.8855 | 0.2492 | 0.2374 | 0.1762 | 0.1683 |
| 60 | 0.8820 | 0.8359 | 0.8994 | 0.9369 | 0.8749 | 0.2238 | 0.2442 | 0.2419 | 0.1394 |

**Fig. 4, kinetics data**

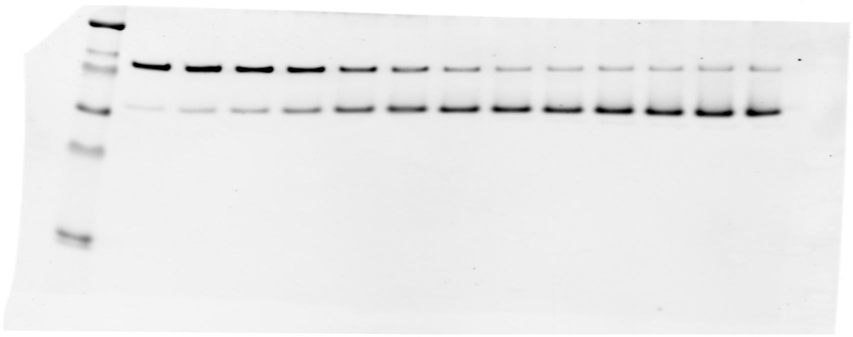

**Fig. 4, repeats for kinetics, TAM/PLE + OmpA, α-OmpA-C**

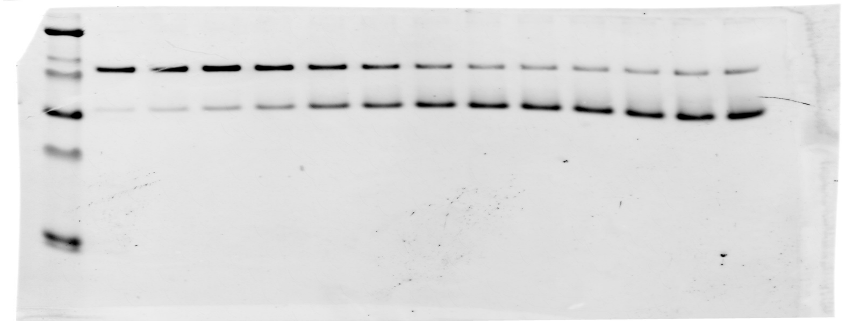

**Fig. 4, repeats for kinetics, TAM/PLE + OmpA, α-OmpA-C**

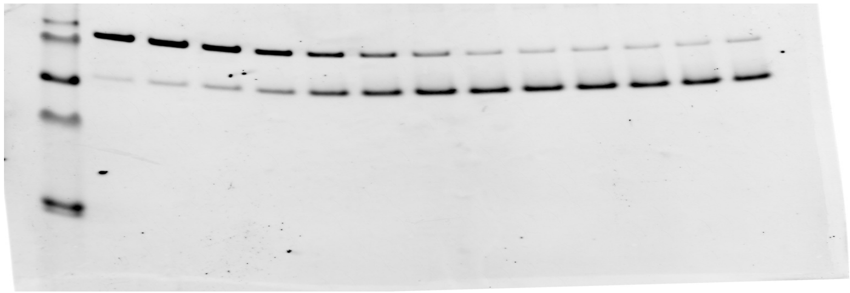

**Fig. 4, repeats for kinetics, TAM/PLE + OmpA, α-OmpA-C**

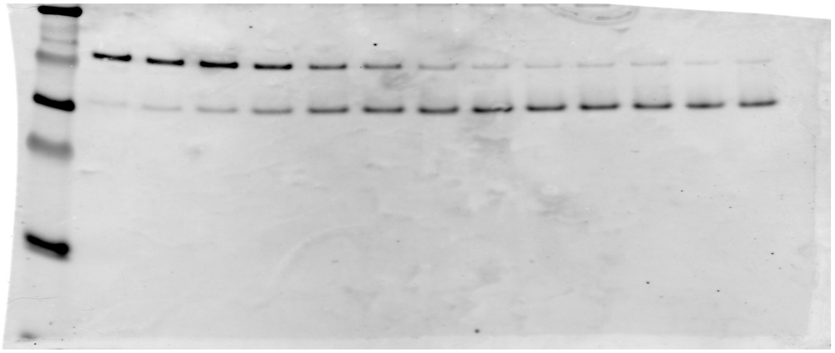

**Fig. 4, repeats for kinetics, TAM/PLE + OmpA, α-OmpA-C**

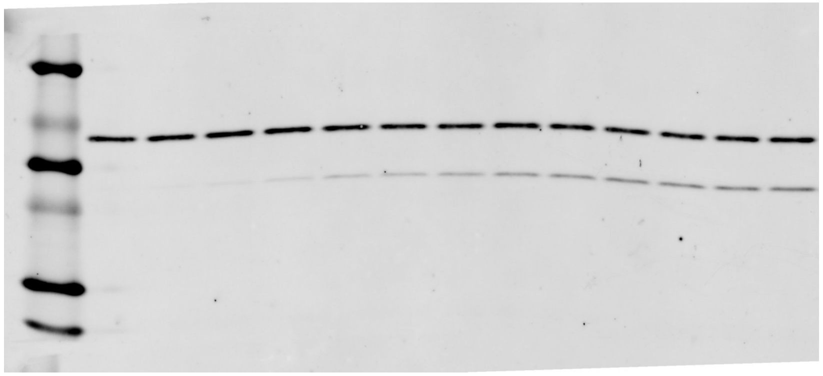

**Fig. 4, repeats for kinetics, TAM/PLE + EspPΔ5’, α-EspP-C**

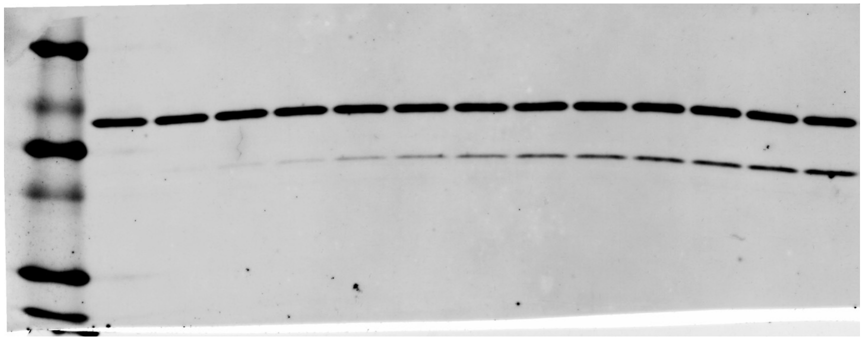

**Fig. 4, repeats for kinetics, TAM/PLE + EspPΔ5’, α-EspP-C**

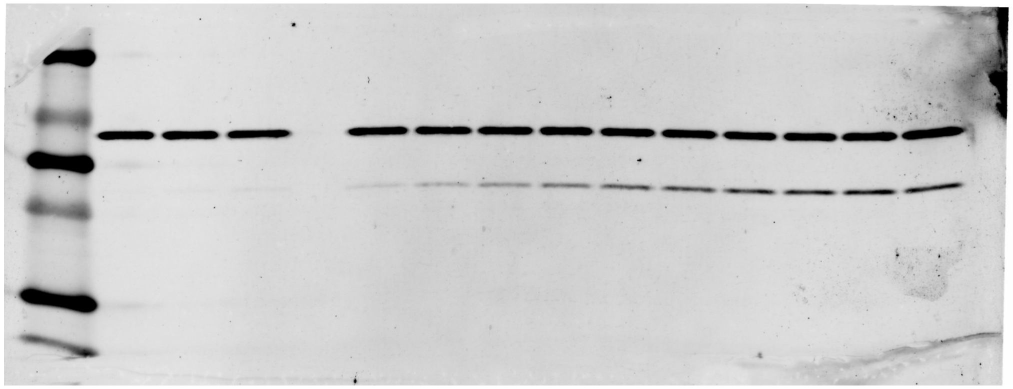

**Fig. 4, repeats for kinetics, TAM/PLE + EspPΔ5’, α-EspP-C**

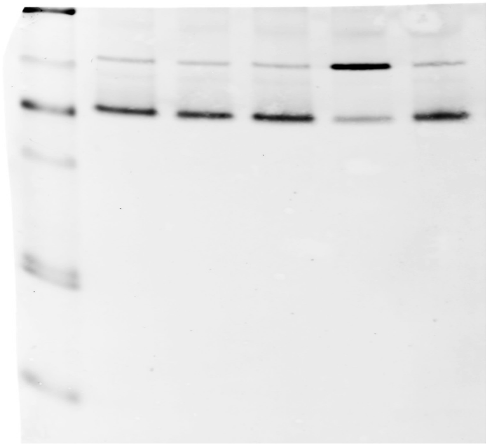

**Fig. 5, top left blot, α-OmpA-C**

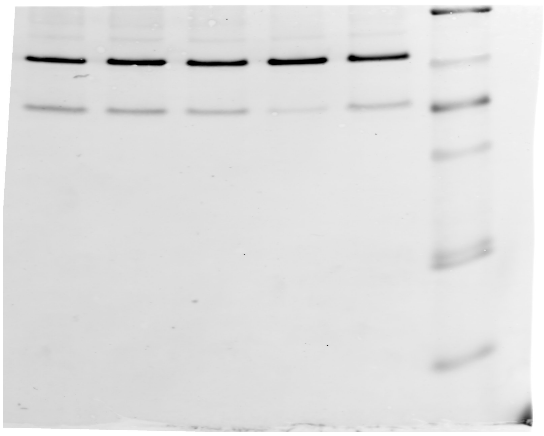

**Fig. 5, middle left blot, α-OmpA-C**

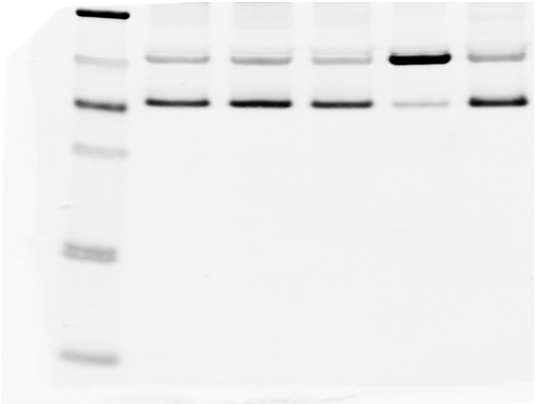

Fig. 5, bottom left blot, α-OmpA-C

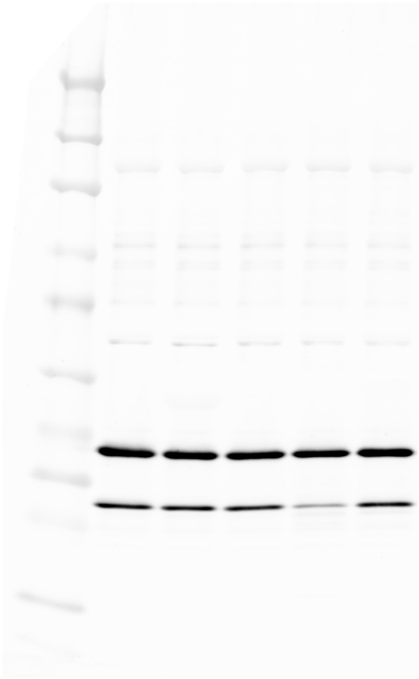

**Fig. 5, top right blot, α-EspP-C**

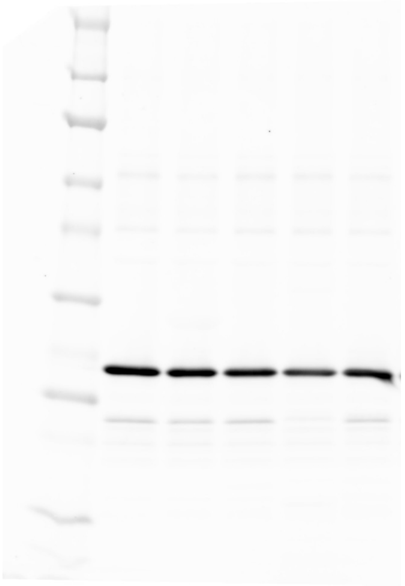

Fig. 5, middle right blot, α-EspP-C

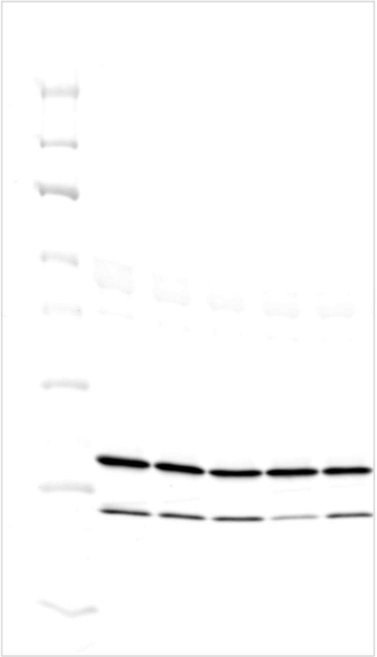

**Fig. 5, bottom right blot, α-EspP-C**

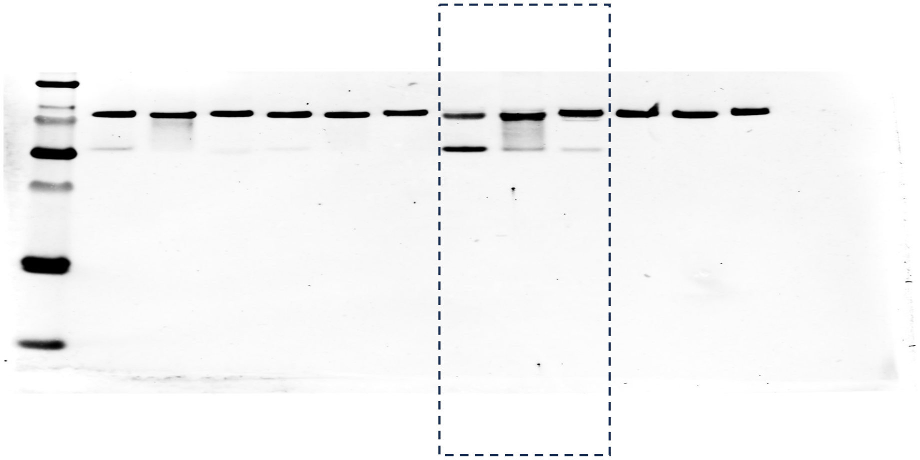

**Fig. 6c, top left blot, α-OmpA-C**

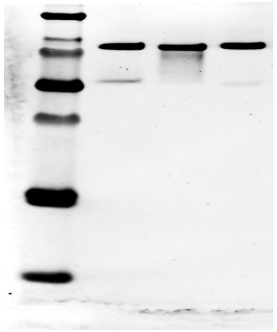

**Fig. 6c, top right blot, α-OmpA-C**

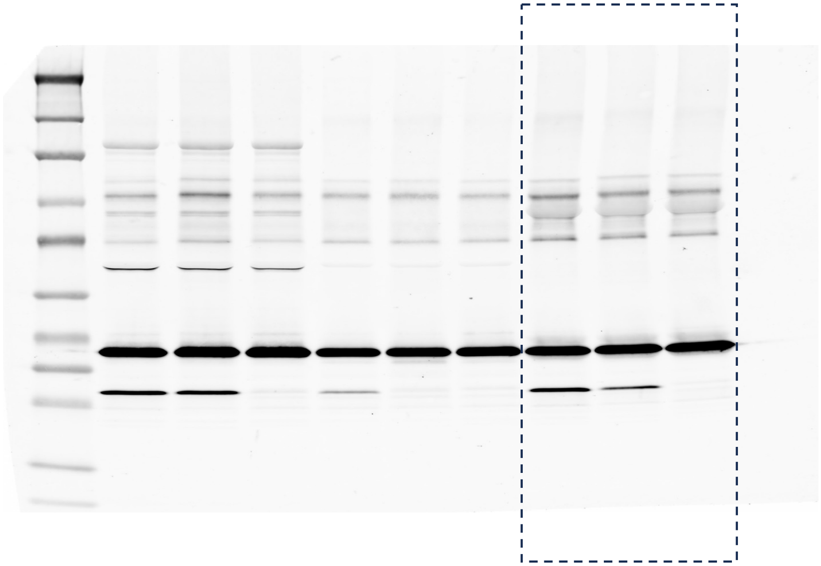

**Fig. 6c, bottom left blot, α-EspP-C**

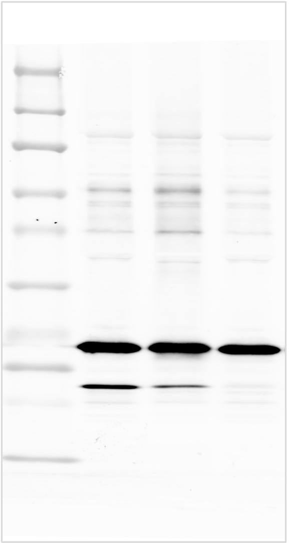

**Fig. 6c, bottom right blot, α-EspP-C**

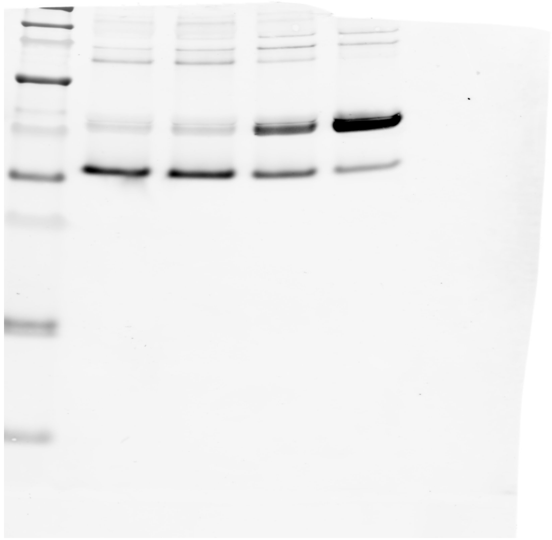

**Fig. 6e, top left blot, α-OmpA-C**

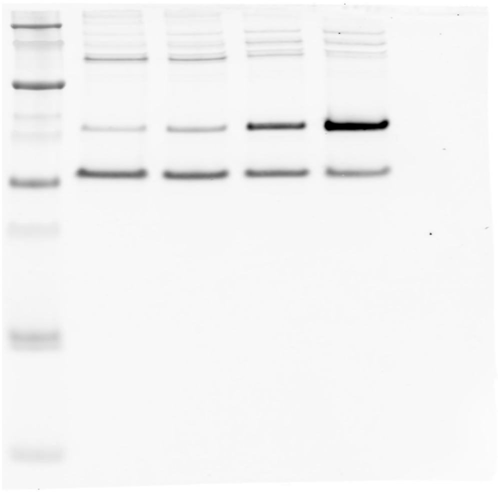

**Fig. 6e, top right blot, α-OmpA-C**

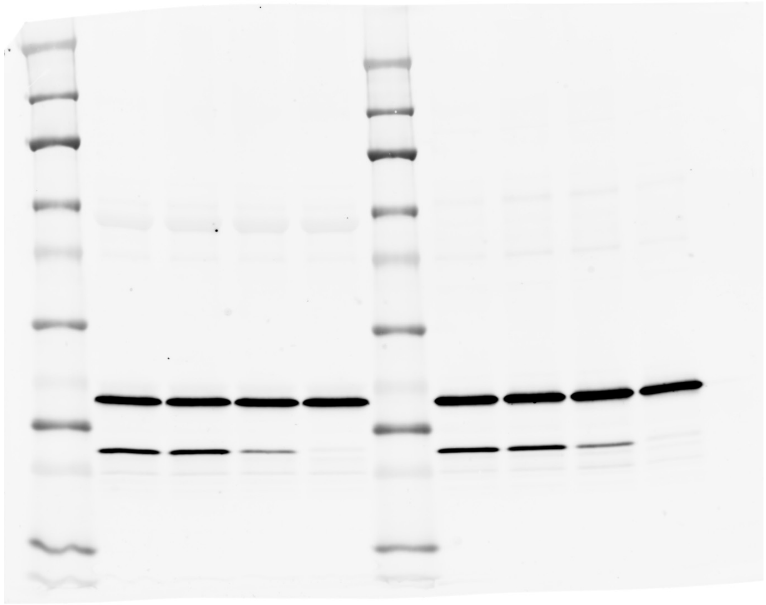

**Fig. 6e, bottom blots, α-EspP-C**

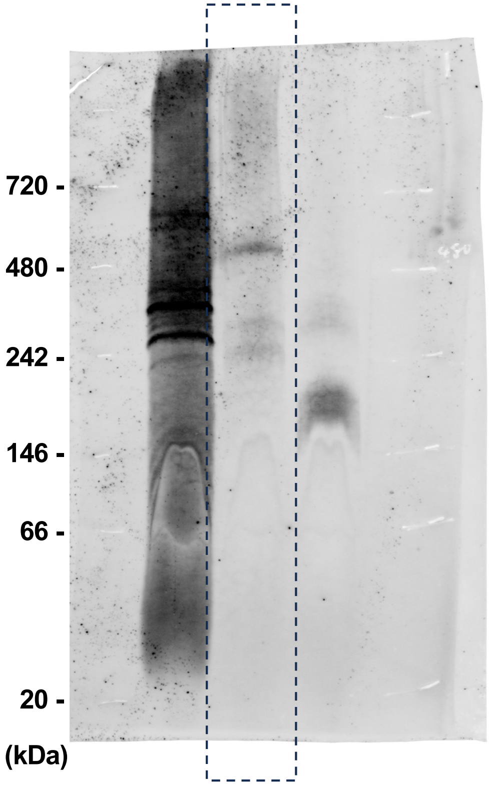


**Fig. S1, left, α-6xHis**

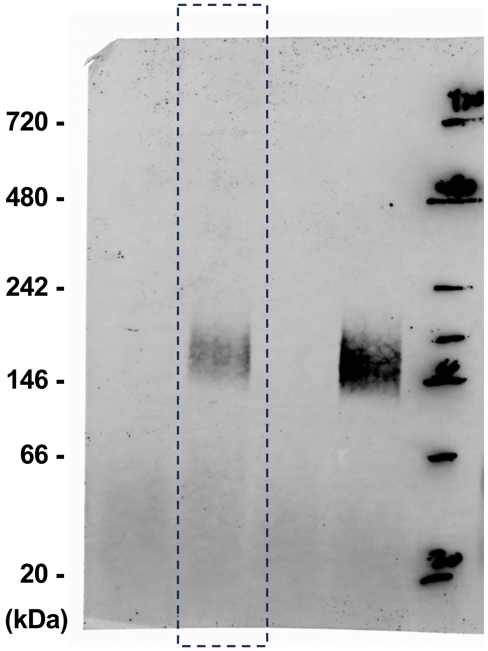


**Fig. S1, right, α-6xHis**

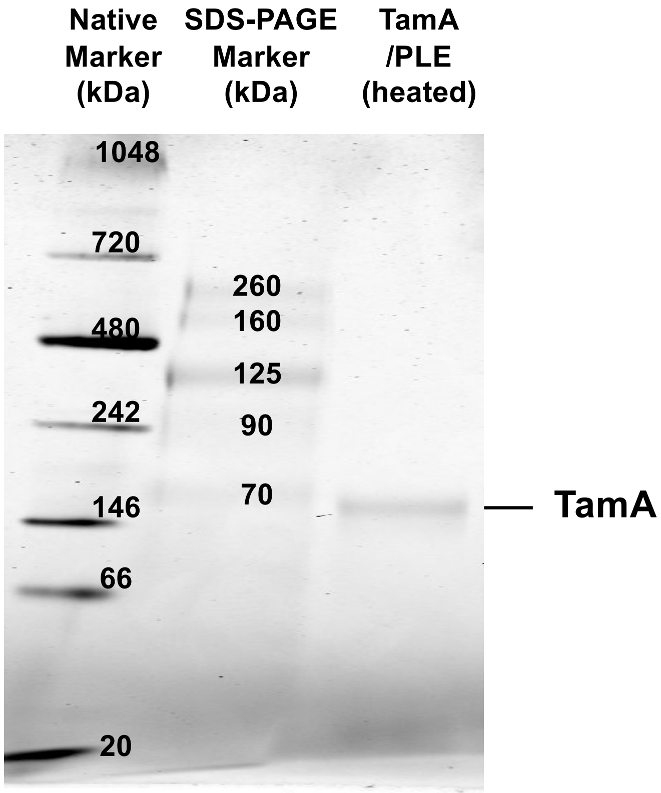


**Fig. S1,** Blue native PAGE shows the native PAGE protein marker (Thermo Fisher NativeMark Unstained Protein Standard) and SDS PAGE protein marker (LICOR Chameleon Duo pre-stained protein marker).

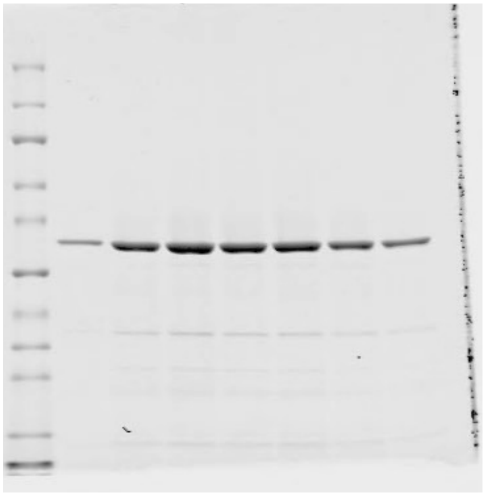

**Fig. S2a, Coomassie blue staining**

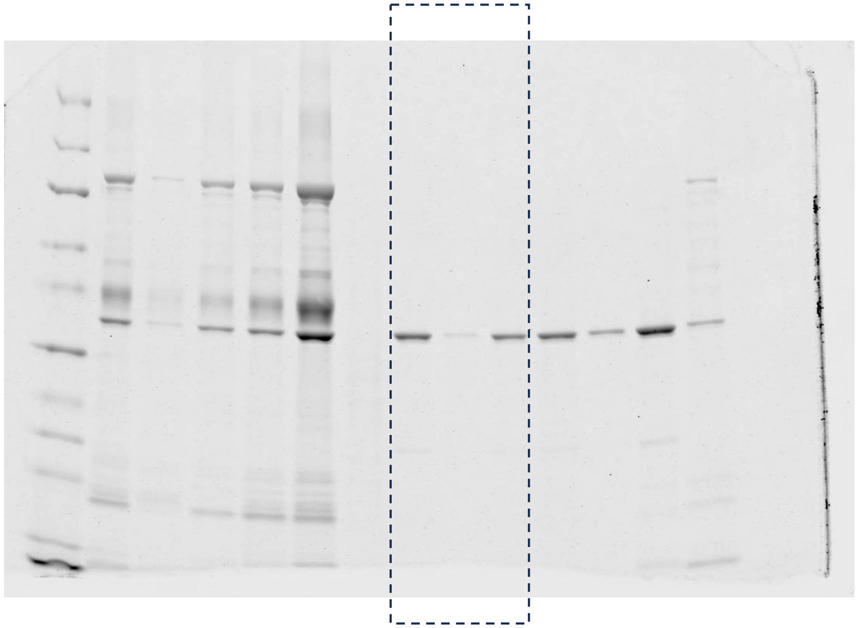

**Fig. S2b, Coomassie blue staining**

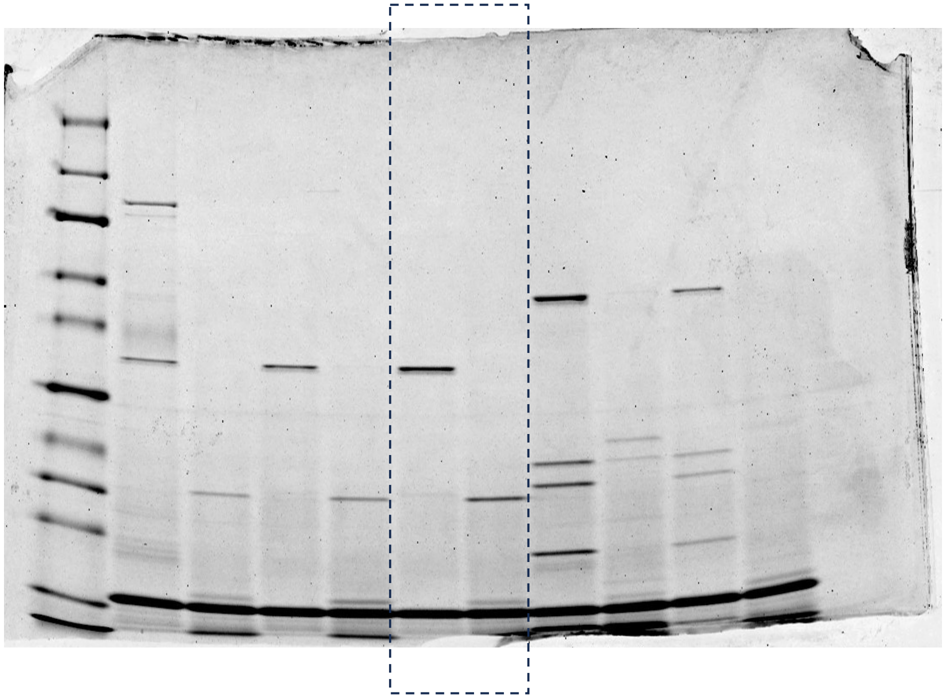

Fig. S2c, Coomassie blue staining

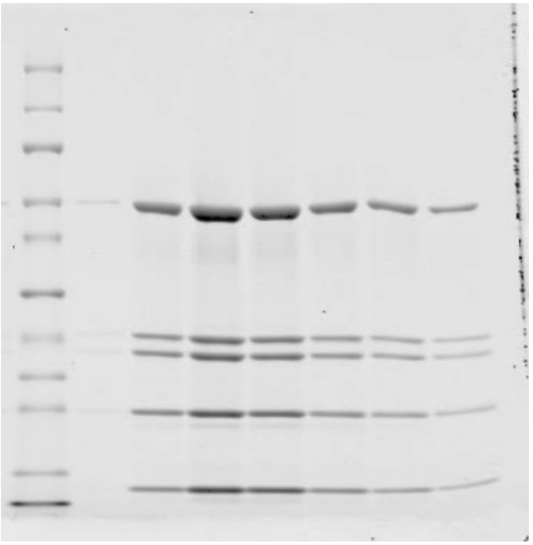

**Fig. S2d, Coomassie blue staining**

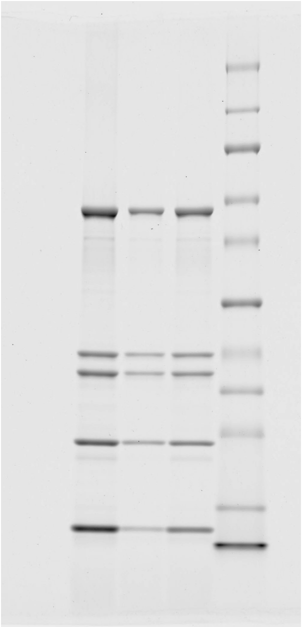

**Fig. S2e, Coomassie blue staining**

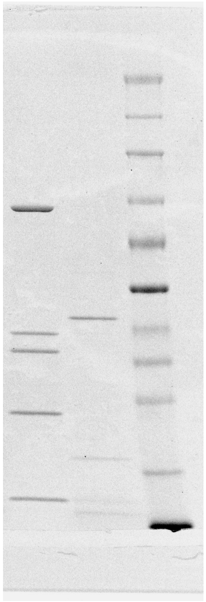

**Fig. S2f, Coomassie blue staining**

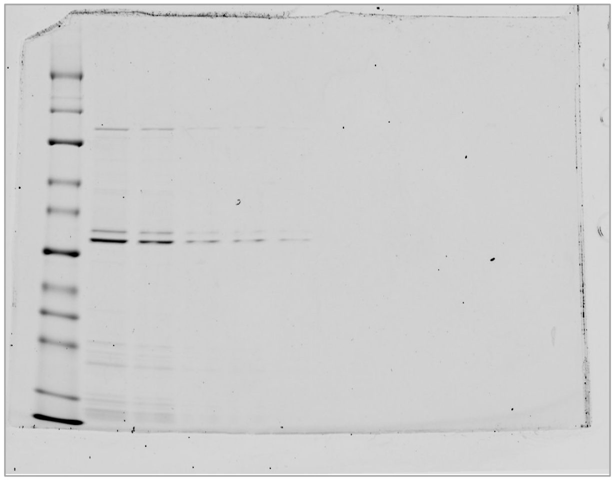

**Fig. S3b, Coomassie blue staining**

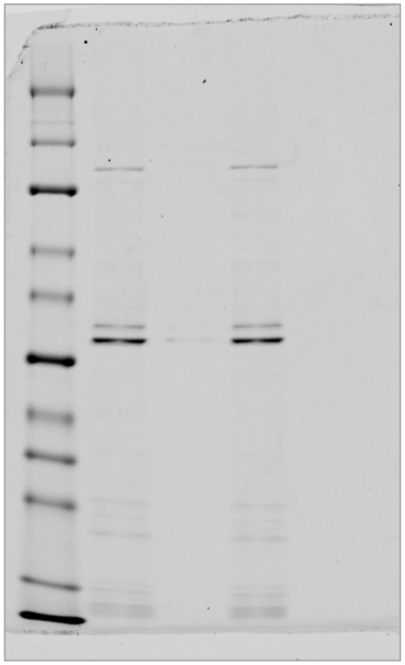

**Fig. S3c, Coomassie blue staining**

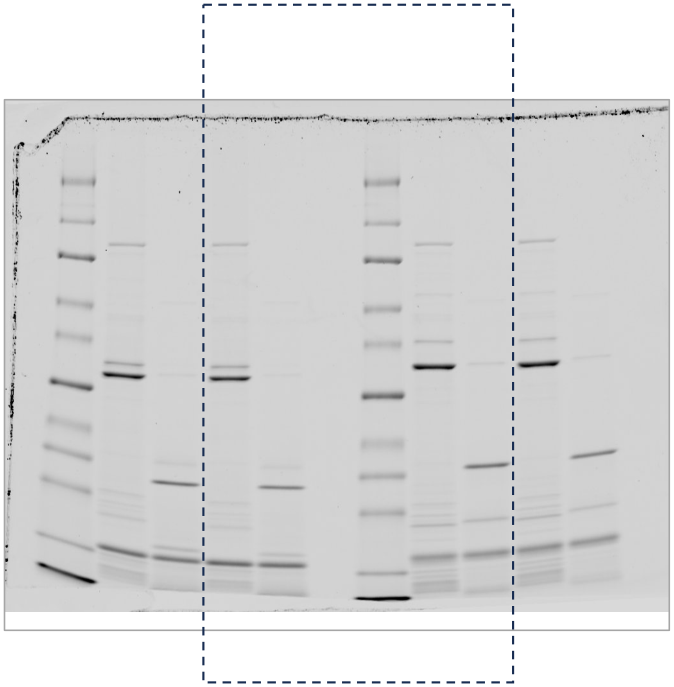

**Fig. S3d, Coomassie blue staining**

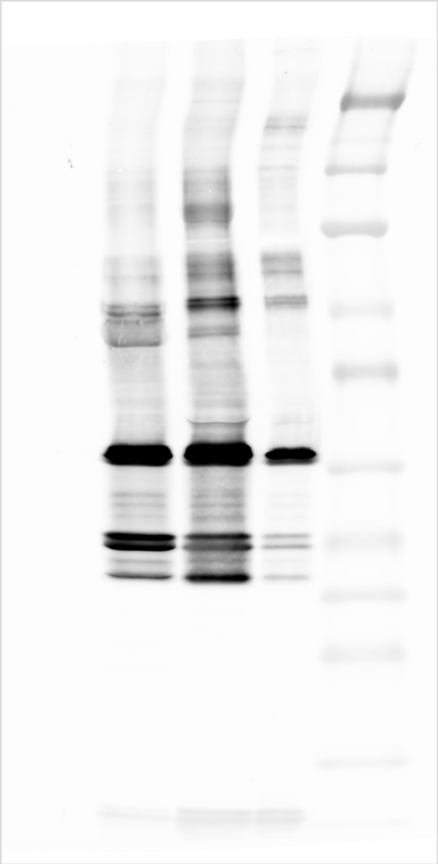

**Fig. S4b, α-Ag43-β**

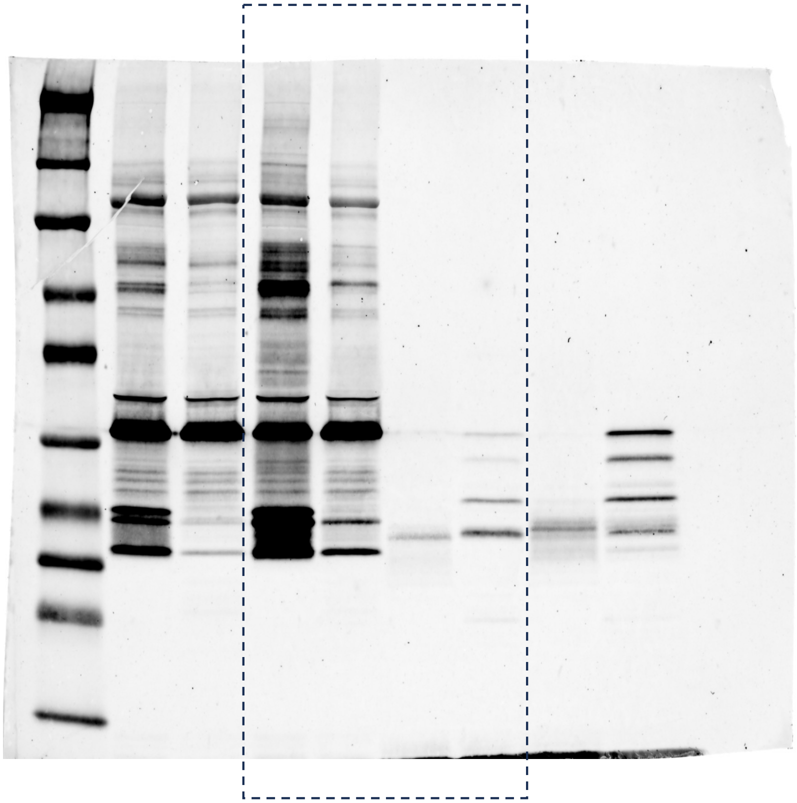

**Fig. S4c, blot-1, α-Ag43-β**

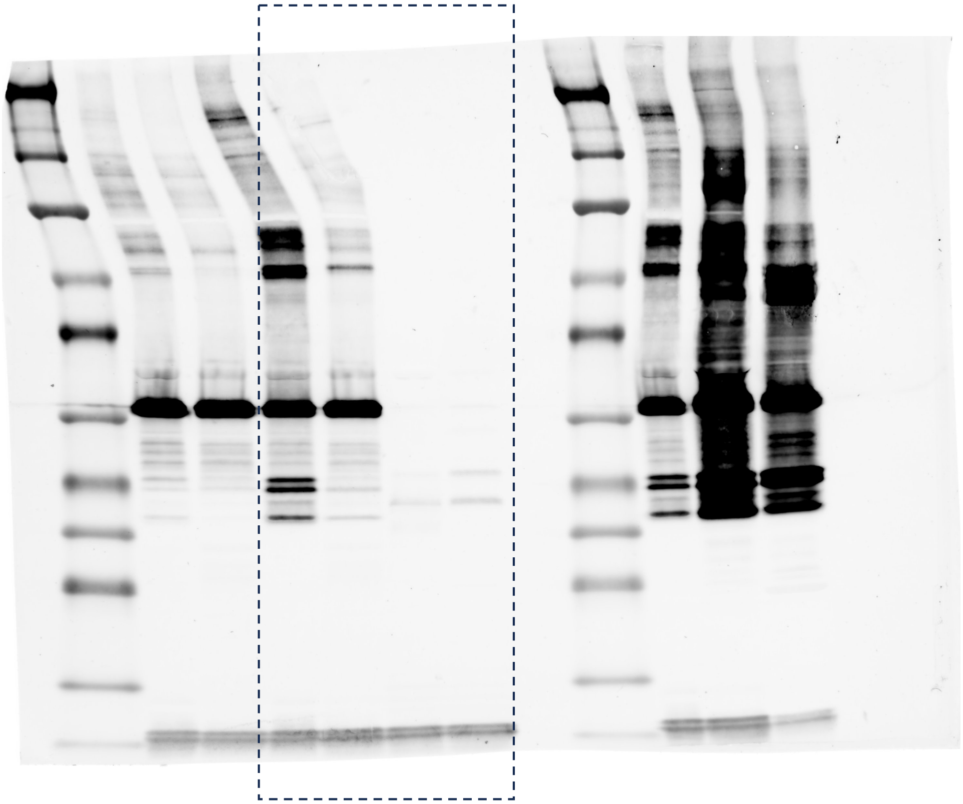

**Fig. S4c, blot-2, α-Ag43-β**

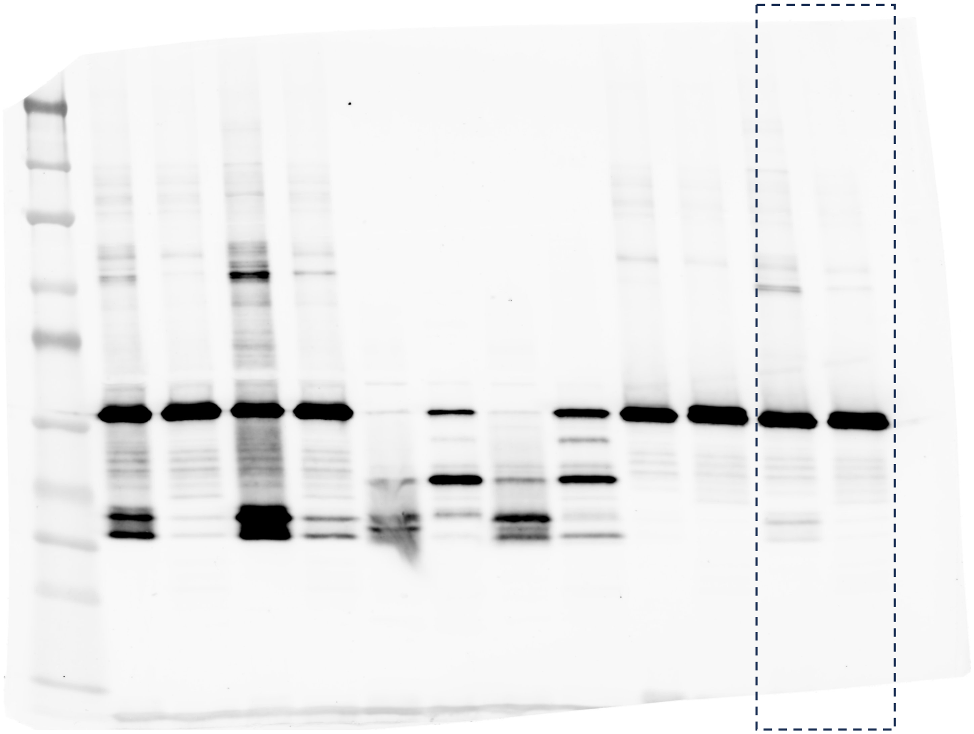

**Fig. S4c, blot-3 left, α-Ag43-β**

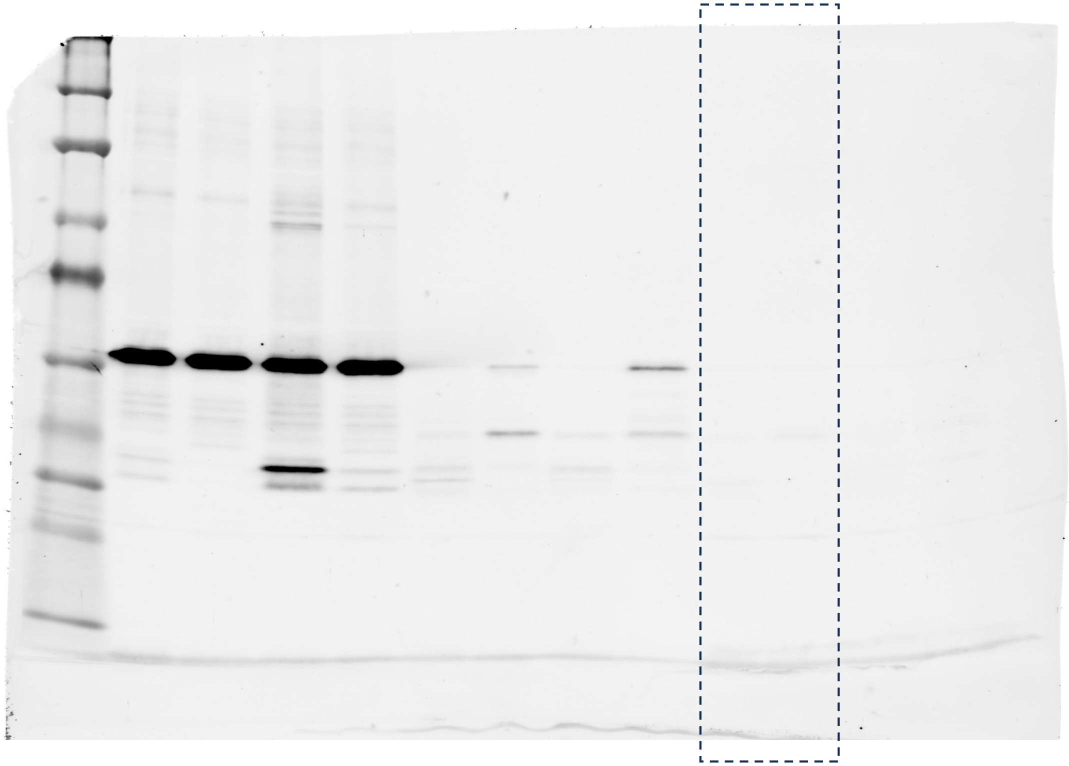

**Fig. S4c, blot-3 right, α-Ag43-β**

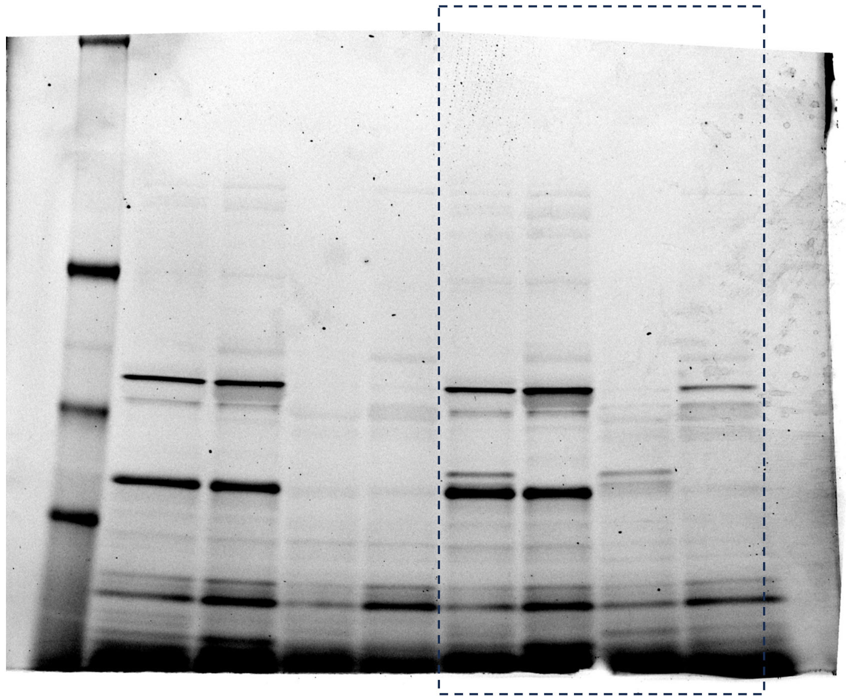

**Fig. S4d**

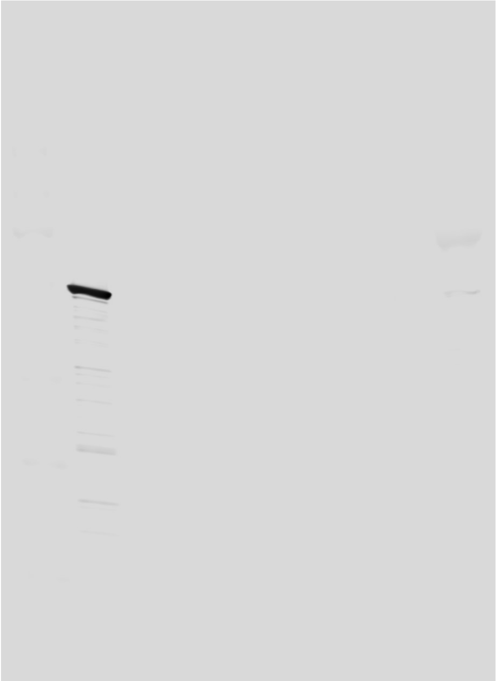

**Fig. S5a, top left blot, α-BamA-C**

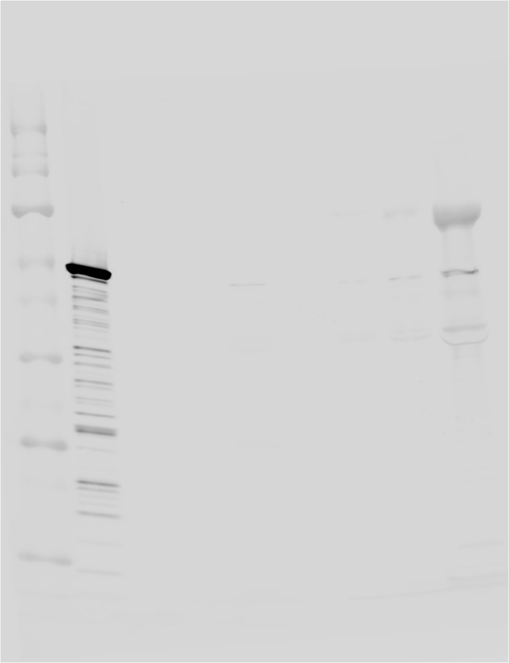

**Fig. S5a, top right blot, α-BamA-C**

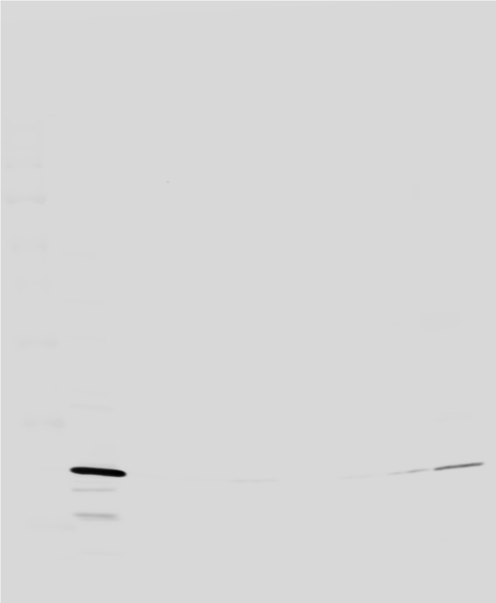

**Fig. S5a, bottom left blot, α-BamD**

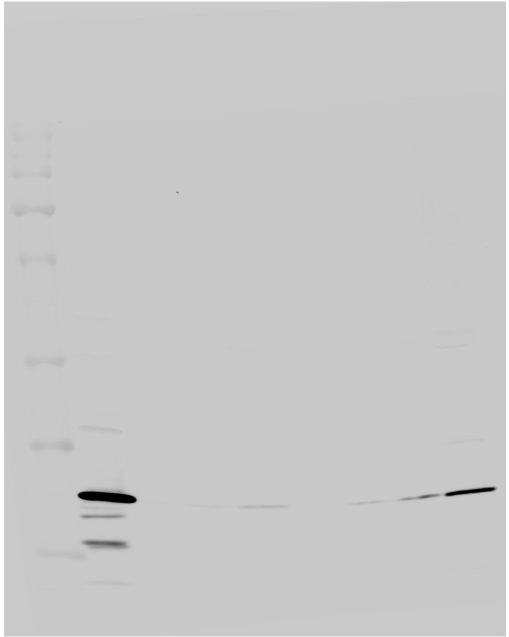

**Fig. S5a, bottom right blot, α-BamD**

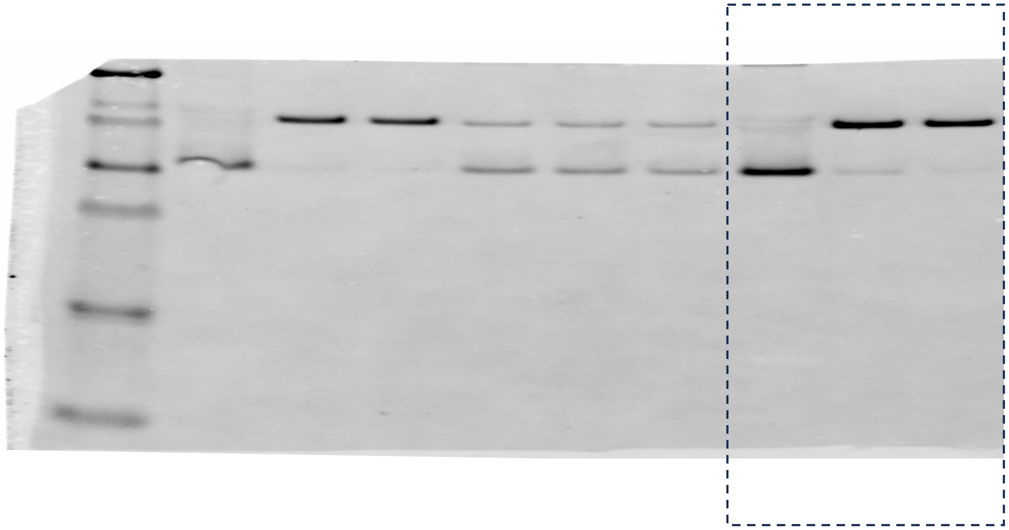

**Fig. S5b, top left blot, α-OmpA-C**

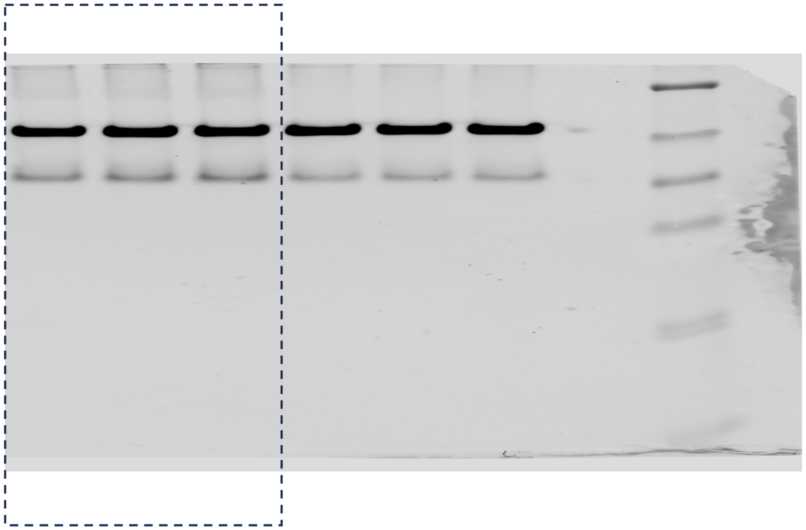

**Fig. S5b, top right blot, α-OmpA-C**

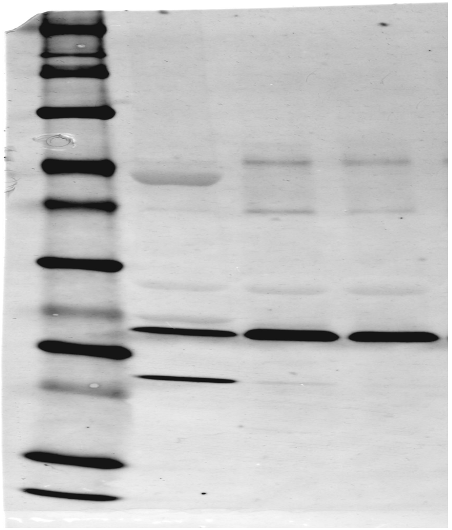

**Fig. S5b, bottom left blot, α-EspP-C**

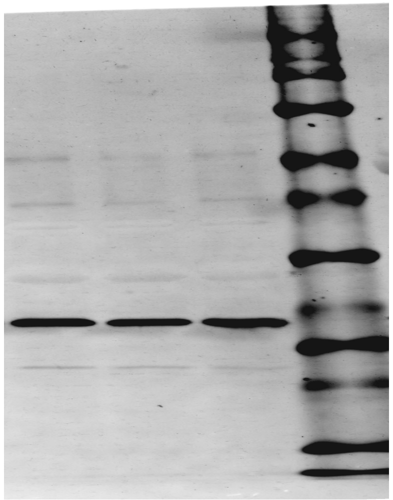

**Fig. S5b, bottom right blot, α-EspP-C**

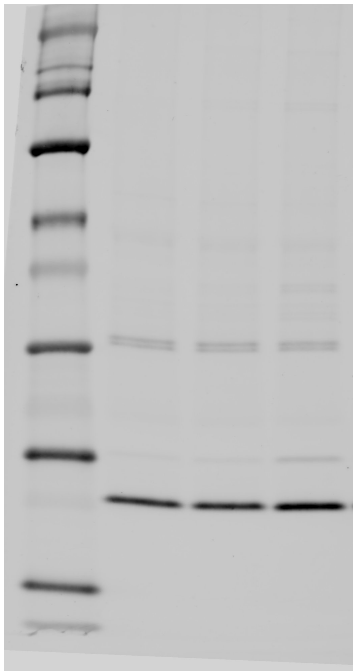

**Fig. S5c, left blot, α-OmpA-C**

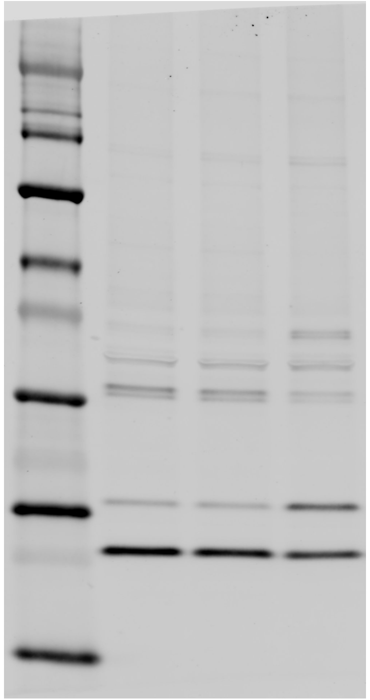

**Fig. S5c, right blot, α-OmpA-C**

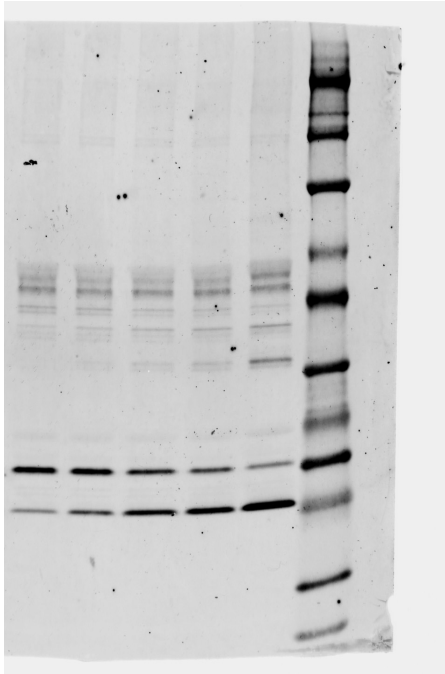

**Fig. S6, top left blot, α-OmpA-C**

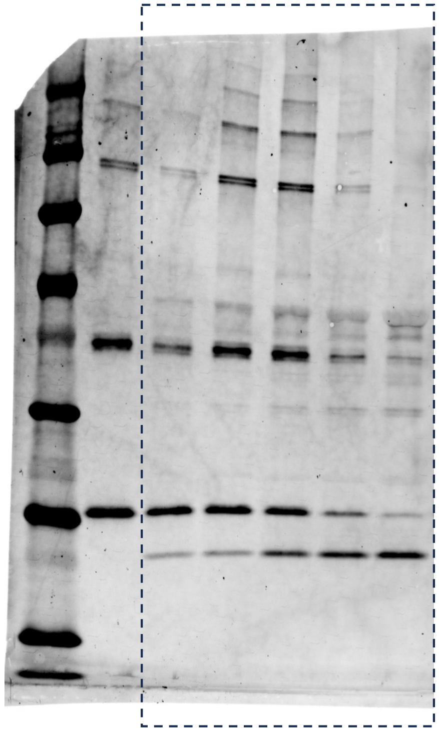

**Fig. S6, top right blot, α-OmpA-C**

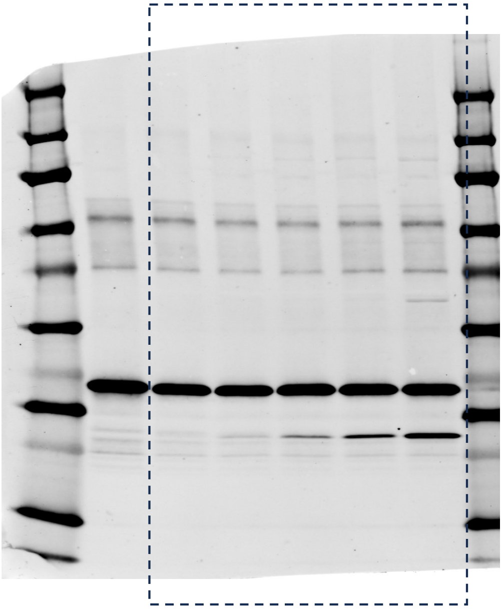

**Fig. S6, middle left blot, α-EspP-C**

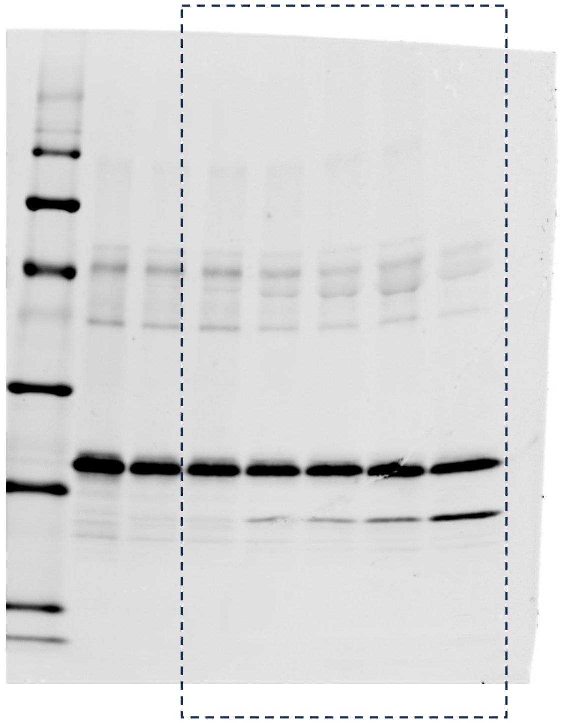

**Fig. S6, middle right blot, α-EspP-C**

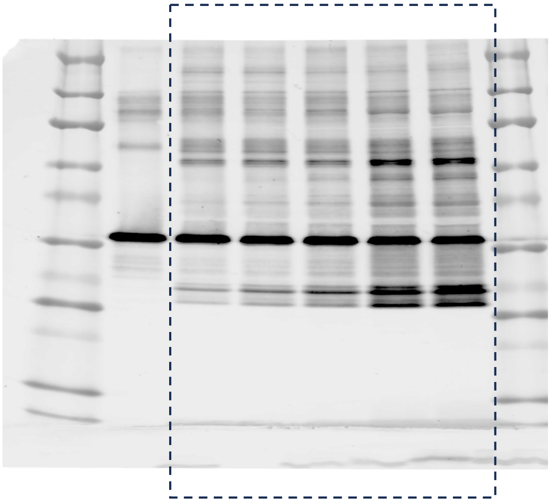

**Fig. S6, bottom left blot, α-Ag43-β**

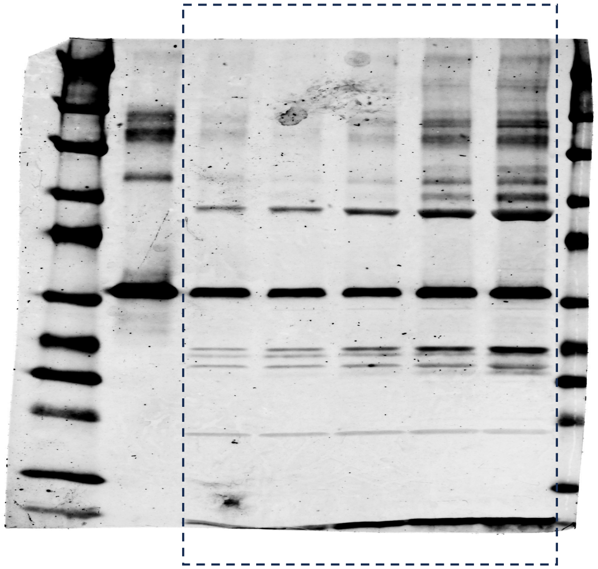

**Fig. S6, bottom right blot, α-Ag43-β**

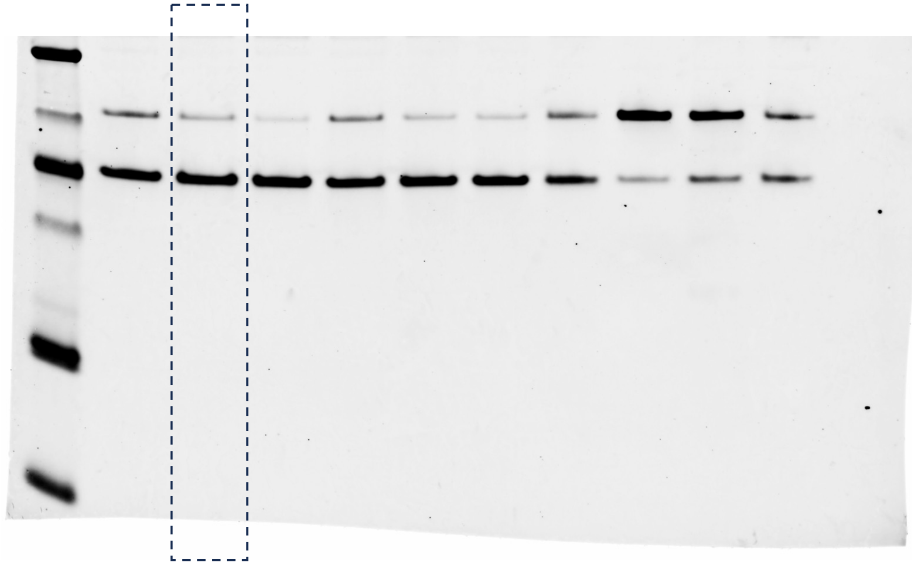

**Fig. S7b, top blot 1, α-OmpA-C**

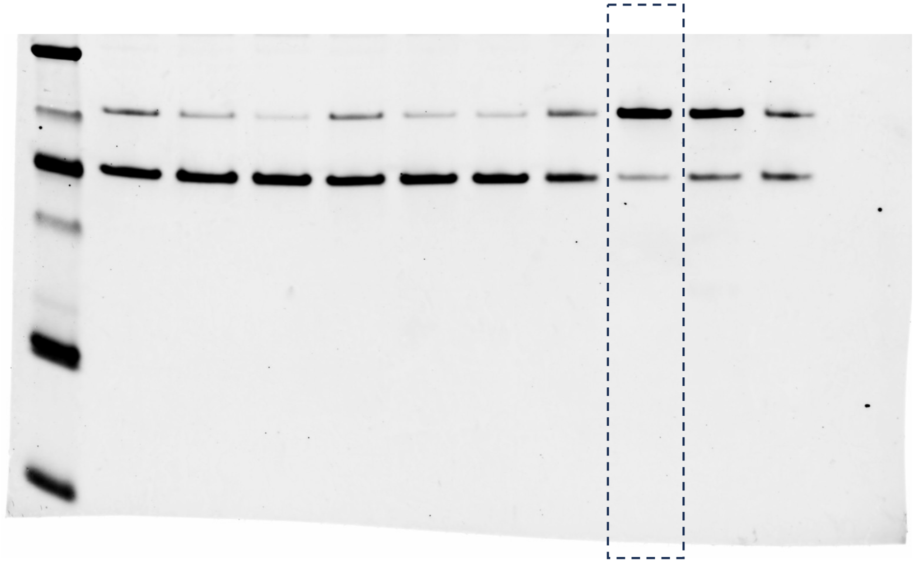

**Fig. S7b, top blot 2, α-OmpA-C**

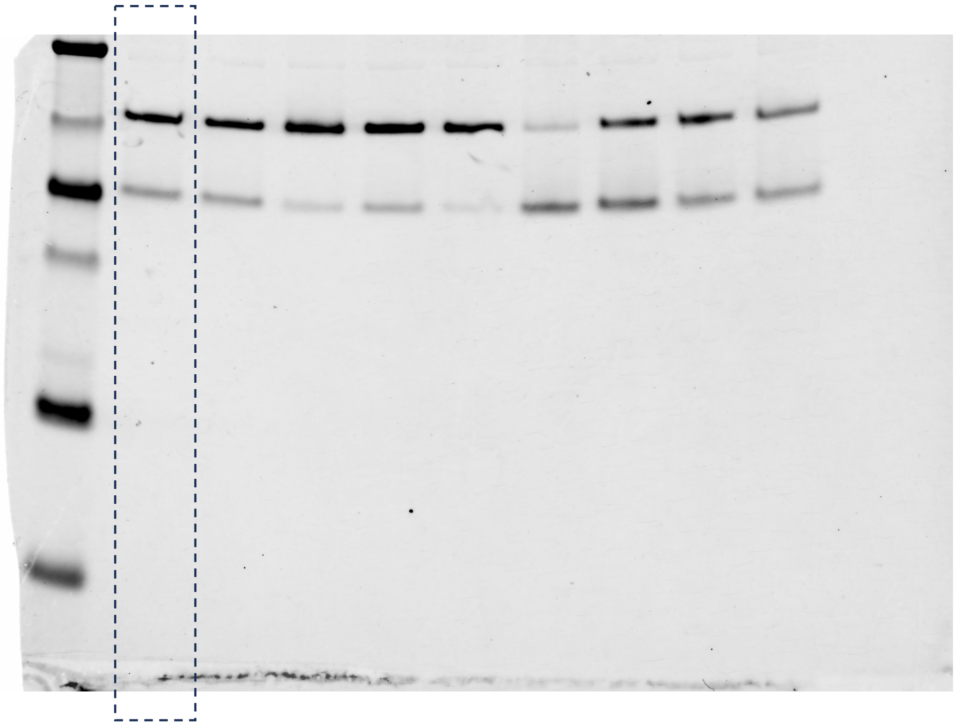

**Fig. S7b, top blot 3, α-OmpA-C**

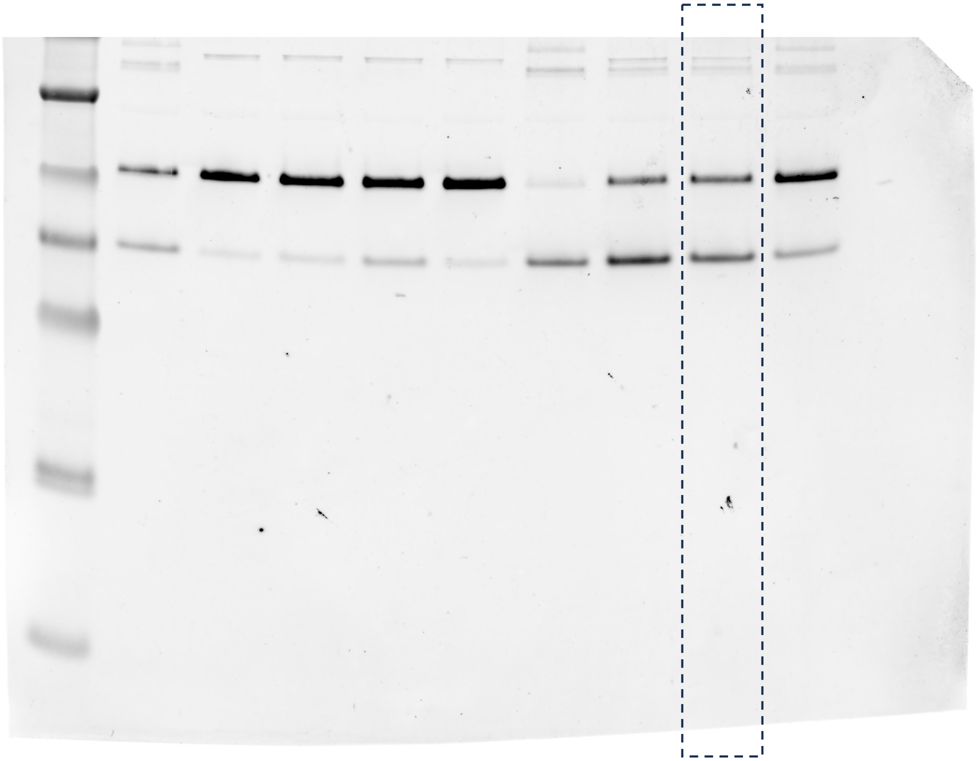

**Fig. S7b, top blot 4, α-OmpA-C**

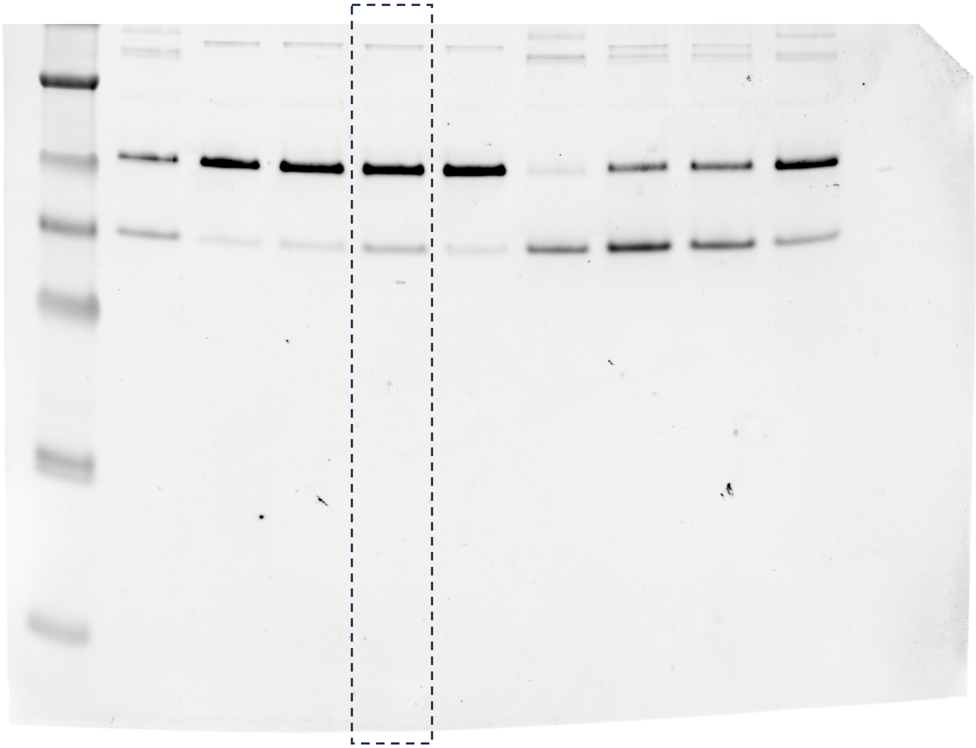

**Fig. S7b, top blot 5, α-OmpA-C**

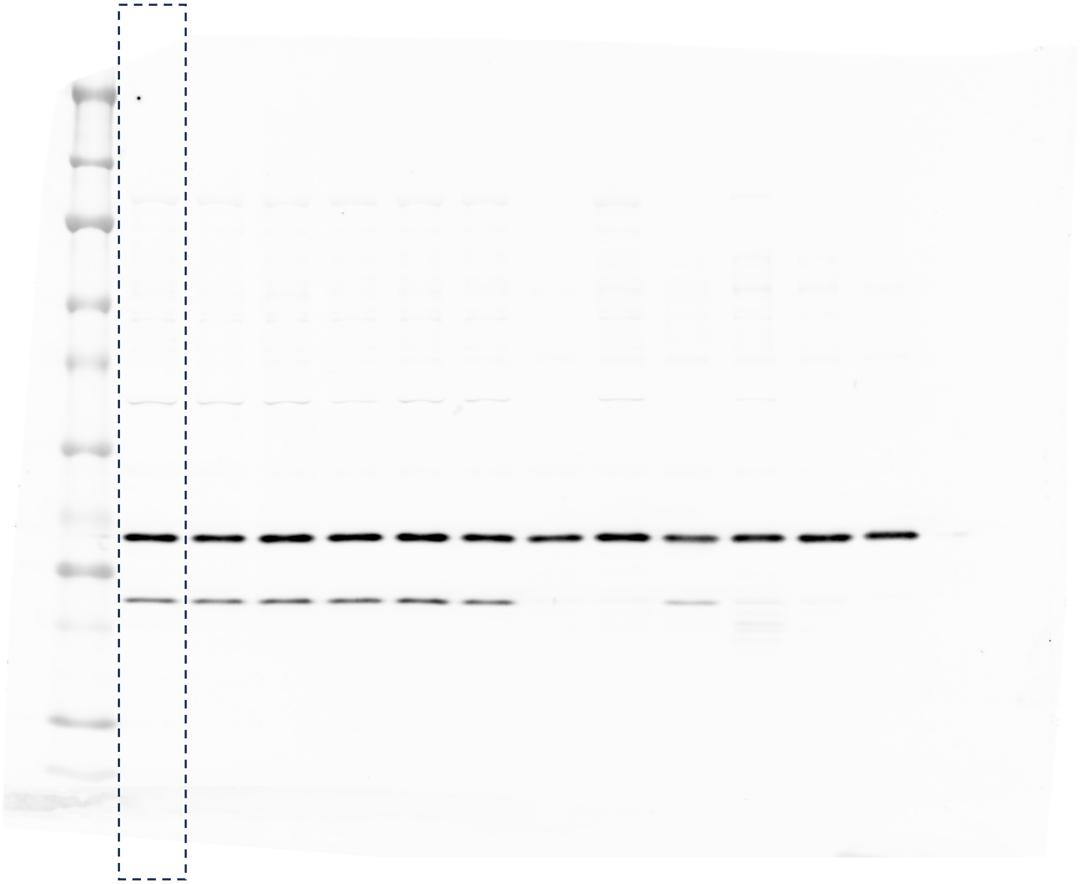

**Fig. S7b, bottom blot 1, α-EspP-C**

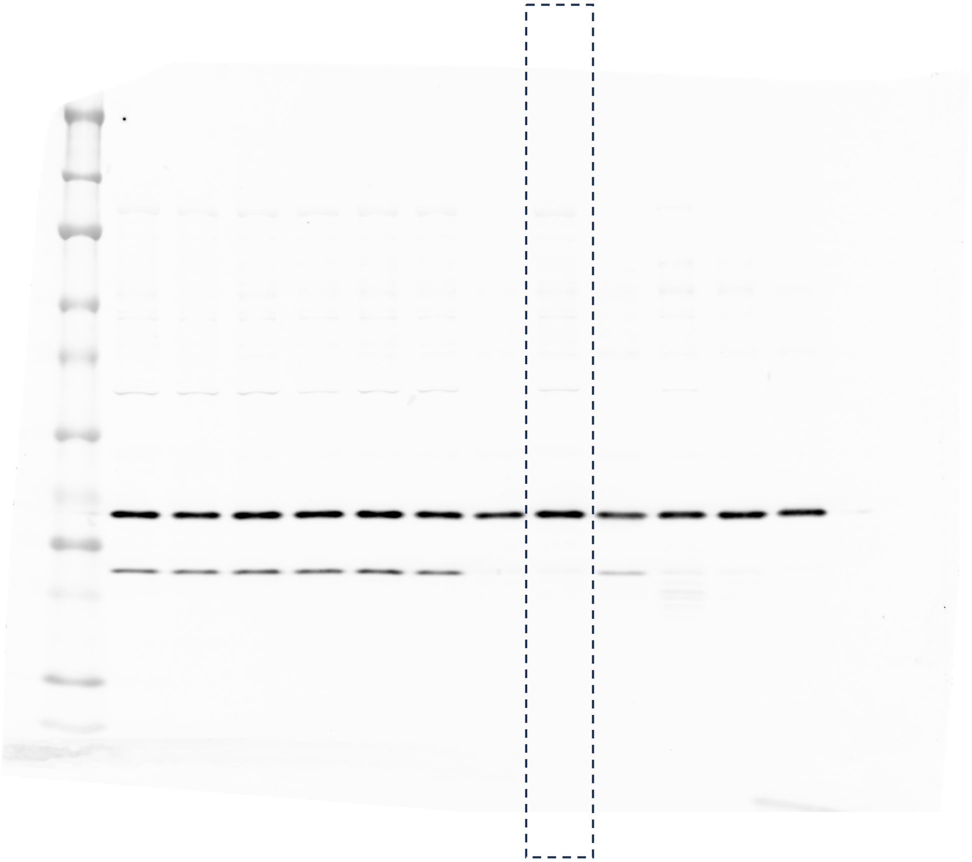

**Fig. S7b, bottom blot 2, α-EspP-C**

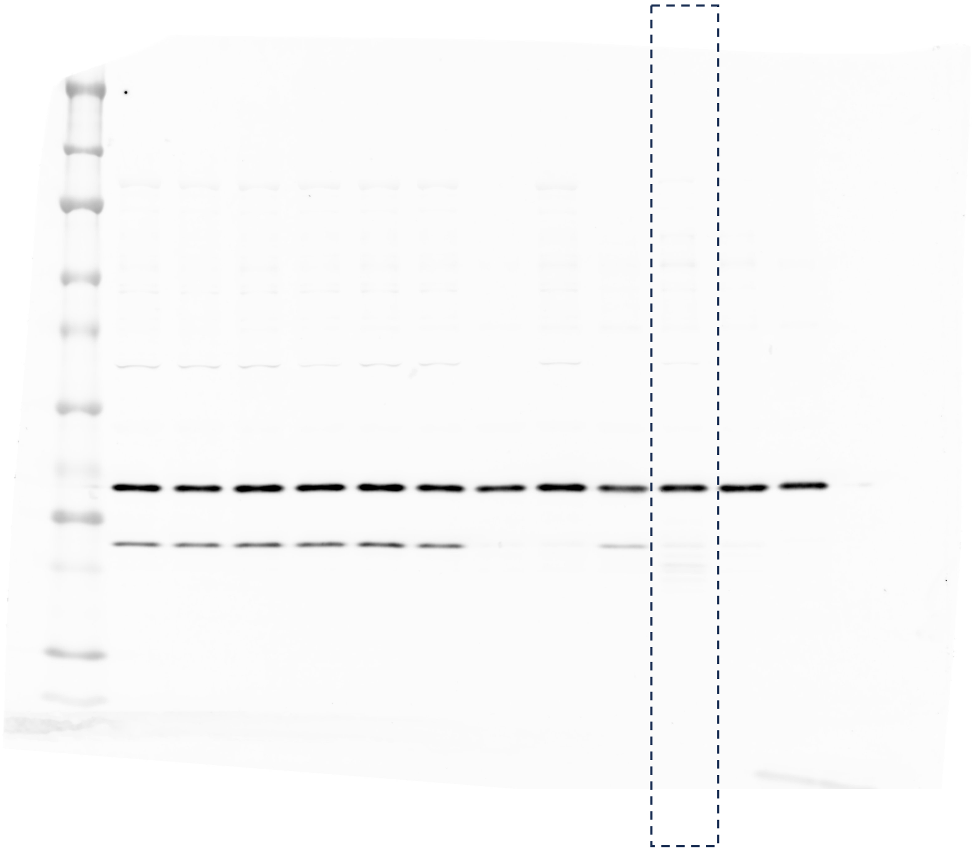

**Fig. S7b, bottom blot 3, α-EspP-C**

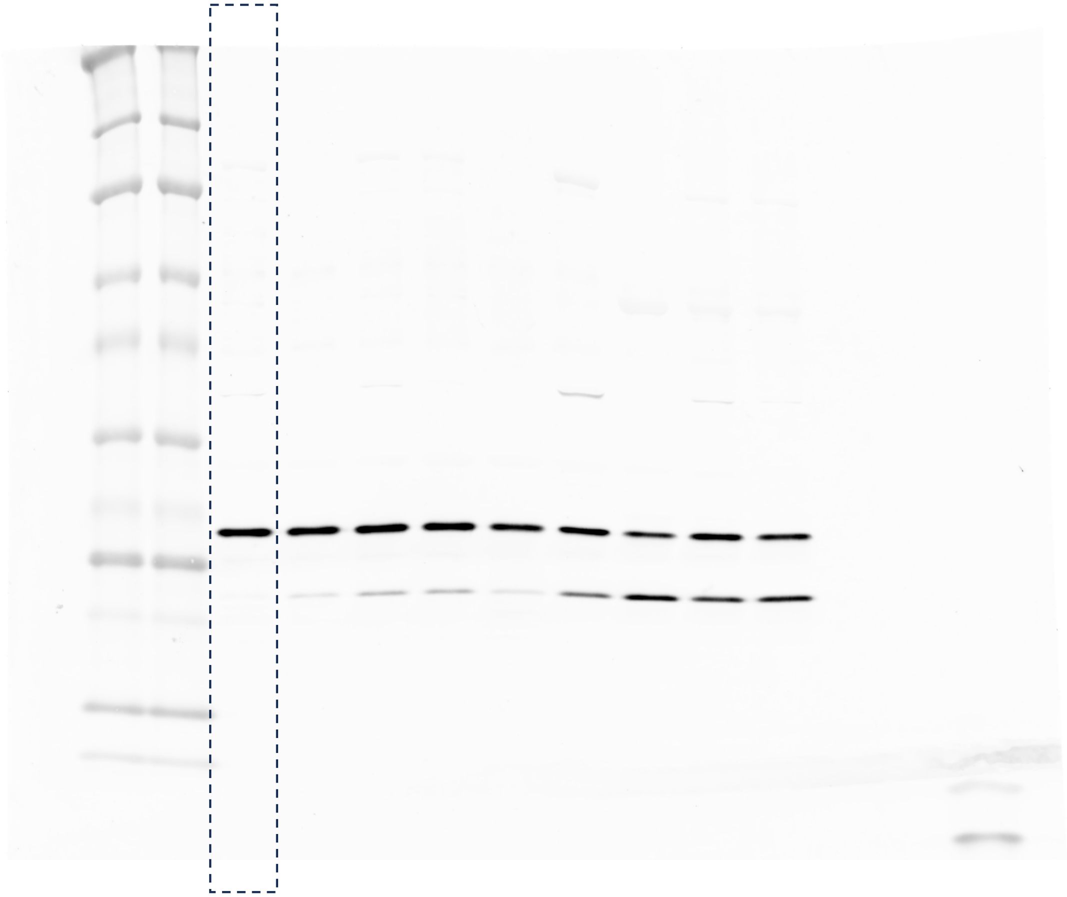

**Fig. S7b, bottom blot 4, α-EspP-C**

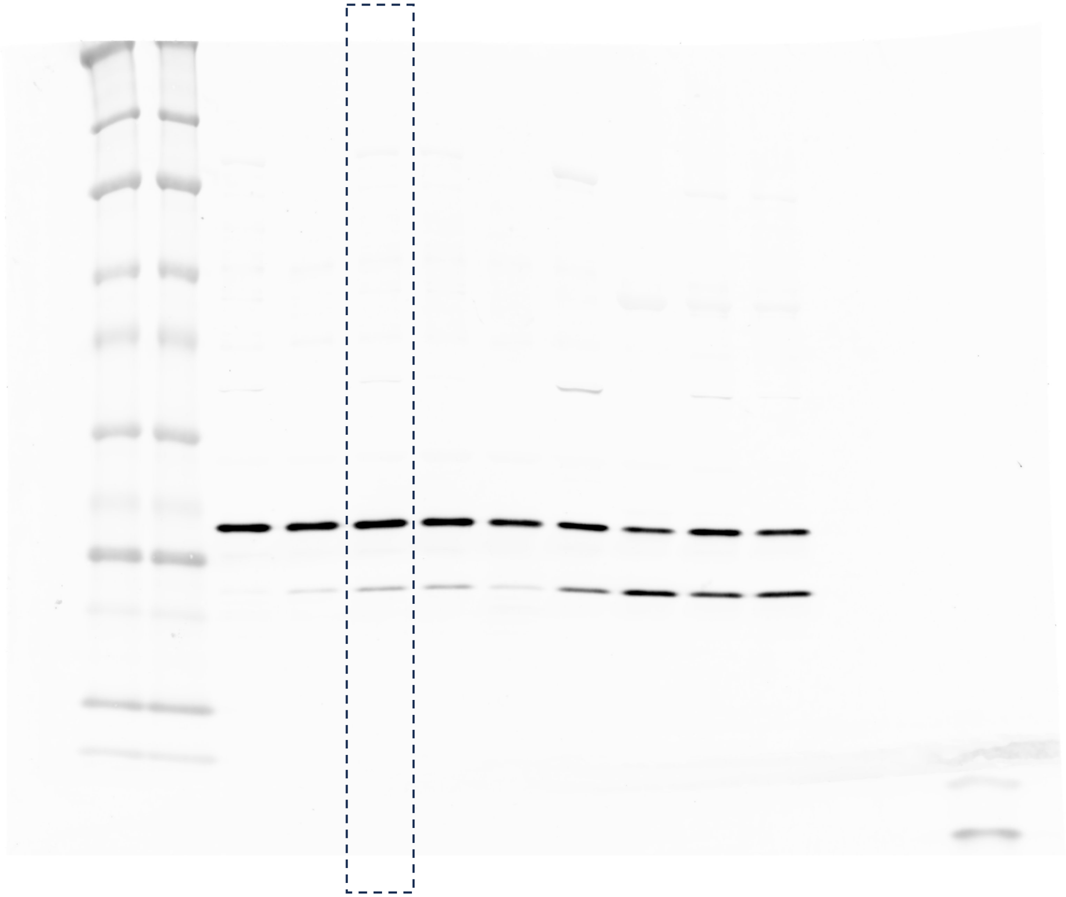

**Fig. S7b, bottom blot 5, α-EspP-C**

**TAM/PLE**

| Chaperone | Test-1 | Test-2 | Test-3 | Average | Standard Error |
|---|---|---|---|---|---|
| **SurA** | 102% | 97% | 100% | 100% | 0.0145 |
| **OsmY** | 102% | 98% | 101% | 100% | 0.0098 |
| **Skp** | 27% | 31% | 24% | 27% | 0.0212 |
| **DegP S210A** | 99% | 96% | 104% | 100% | 0.0237 |

**TamA/PLE**

| Chaperone | Test-1 | Test-2 | Test-3 | Average | Standard Error |
|---|---|---|---|---|---|
| **SurA** | 90% | 107% | 97% | 98% | 0.0504 |
| **OsmY** | 97% | 97% | 84% | 92% | 0.0436 |
| **Skp** | 17% | 17% | 21% | 18% | 0.0121 |
| **DegP S210A** | 107% | 105% | 90% | 101% | 0.0531 |

**BAM/PLE**

| Chaperone | Test-1 | Test-2 | Test-3 | Average | Standard Error |
|---|---|---|---|---|---|
| **SurA** | 101% | 108% | 102% | 104% | 0.0226 |
| **OsmY** | 102% | 102% | 97% | 100% | 0.0161 |
| **Skp** | 21% | 26% | 19% | 22% | 0.0219 |
| **DegP S210A** | 98% | 102% | 94% | 98% | 0.0239 |

# Fig. S8a, data

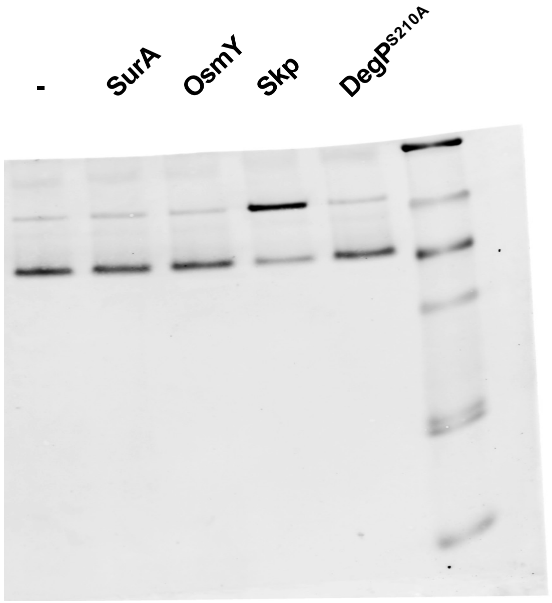


Fig. S8a, repeats for TAM/PLE + OmpA, α-OmpA-C

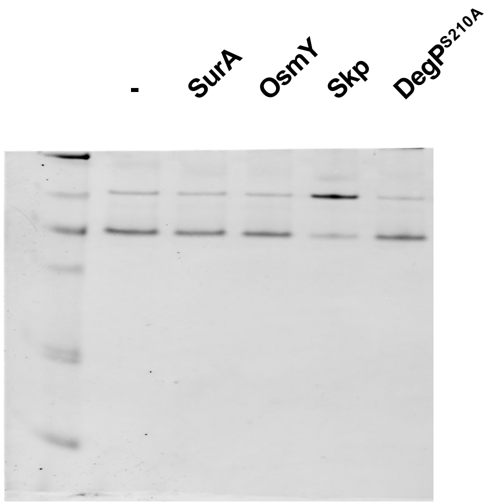


**Fig. S8a, repeats for TAM/PLE + OmpA, α-OmpA-C**

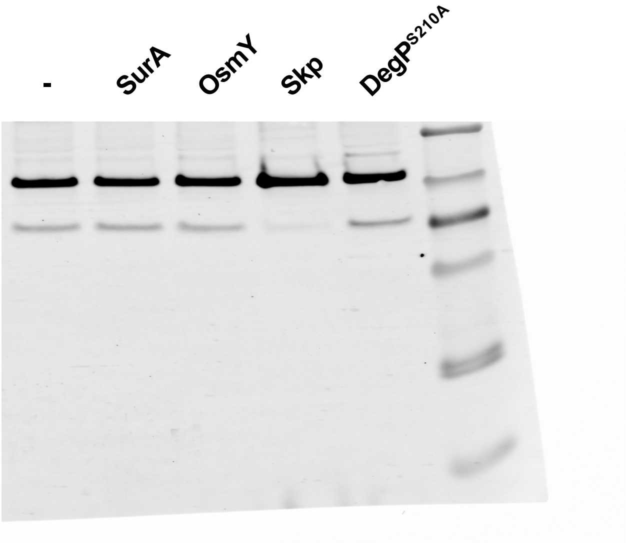


**Fig. S8a, repeats for TamA/PLE + OmpA, α-OmpA-C**

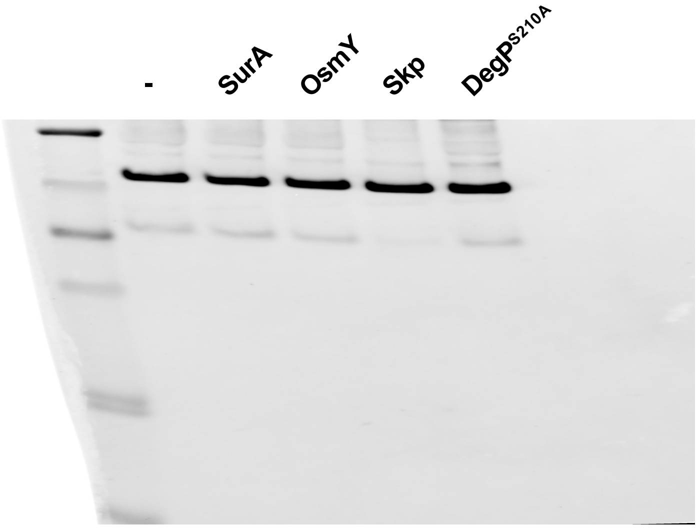


Fig. S8a, repeats for TamA/PLE + OmpA, α-OmpA-C

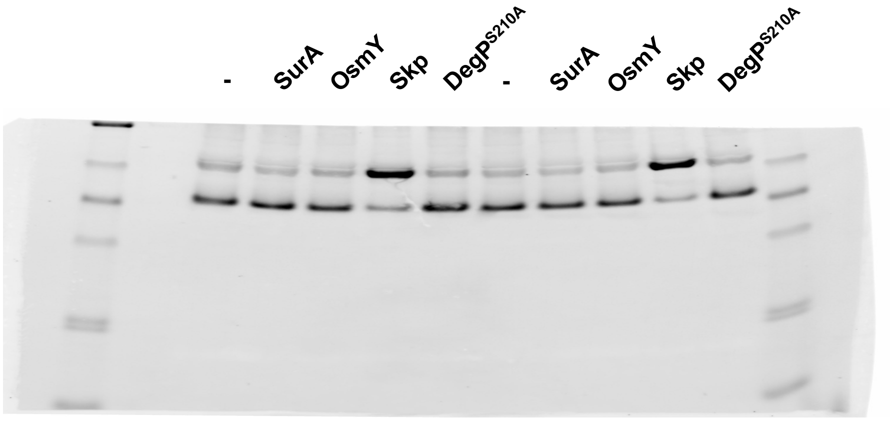


**Fig. S8a, repeats for BAM/PLE + OmpA, α-OmpA-C**

**TAM/PLE**

| Chaperone | Test-1 | Test-2 | Test-3 | Average | Standard Error |
|---|---|---|---|---|---|
| **SurA** | 97% | 108% | 110% | 105% | 0.0404 |
| **OsmY** | 96% | 105% | 110% | 104% | 0.0396 |
| **Skp** | 34% | 33% | 53% | 40% | 0.0662 |
| **DegP S210A** | 91% | 92% | 96% | 93% | 0.0138 |

**TamA/PLE**

| Chaperone | Test-1 | Test-2 | Test-3 | Average | Standard Error |
|---|---|---|---|---|---|
| **SurA** | 80% | 105% | 73% | 86% | 0.0977 |
| **OsmY** | 111% | 102% | 59% | 91% | 0.1617 |
| **Skp** | 19% | 13% | 0% | 11% | 0.0553 |
| **DegP S210A** | 99% | 67% | 83% | 83% | 0.0940 |

**BAM/PLE**

| Chaperone | Test-1 | Test-2 | Test-3 | Average | Standard Error |
|---|---|---|---|---|---|
| **SurA** | 90% | 95% | 93% | 93% | 0.0144 |
| **OsmY** | 102% | 109% | 96% | 102% | 0.0366 |
| **Skp** | 47% | 7% | 20% | 25% | 0.1163 |
| **DegP S210A** | 98% | 104% | 69% | 90% | 0.1065 |

**Fig. S8b, data**

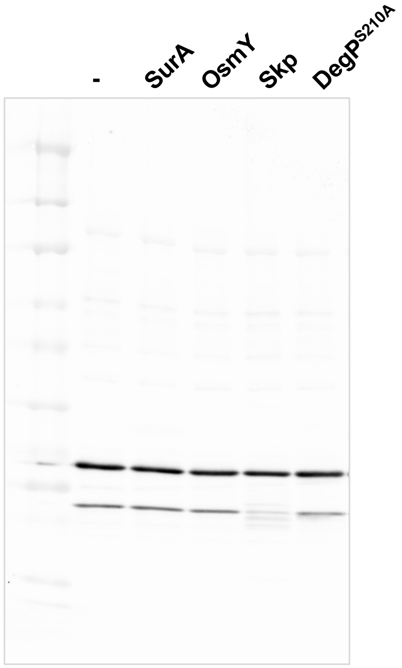


**Fig. S8b, repeats for TAM/PLE + EspPΔ5', α-EspP-C**

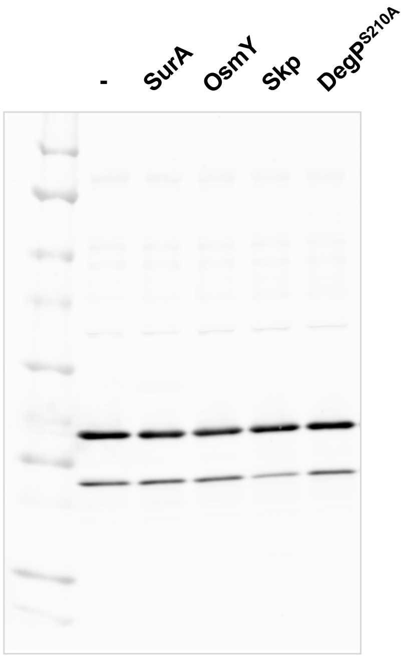


**Fig. S8b, repeats for TAM/PLE + EspPΔ5’, α-EspP-C**

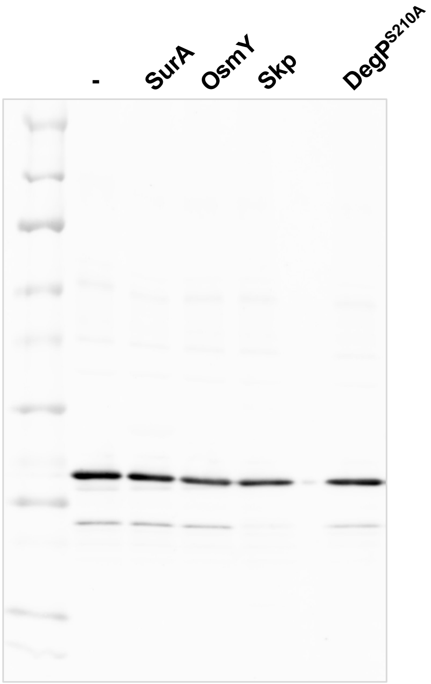


**Fig. S8b, repeats for TamA/PLE + EspPΔ5’, α-EspP-C**

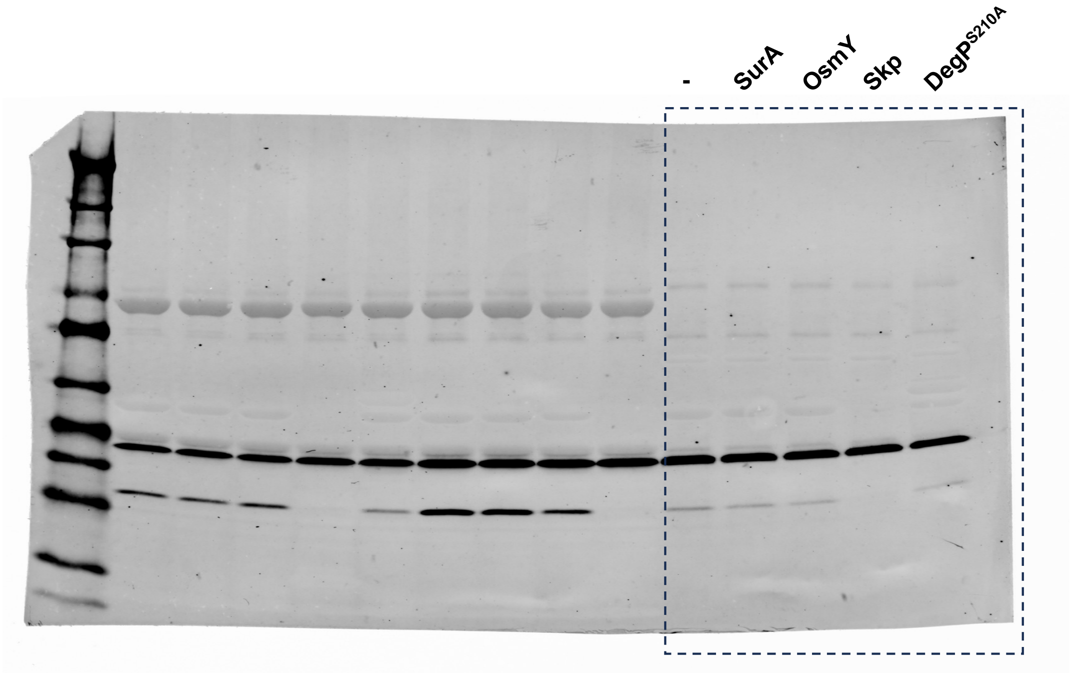


**Fig. S8b, repeats for TamA/PLE + EspPΔ5’, α-EspP-C**

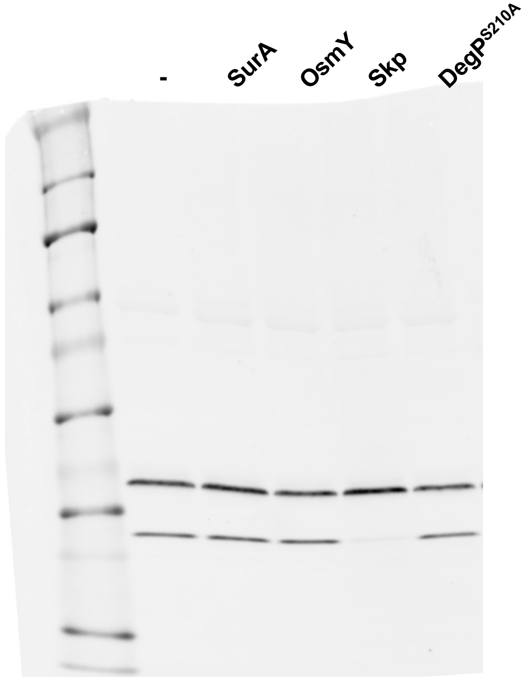


**Fig. S8b, repeats for BAM/PLE + EspPΔ5', α-EspP-C**

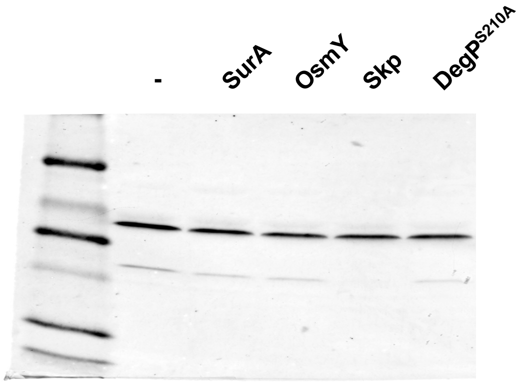


**Fig. S8b, repeats for BAM/PLE + EspPΔ5’, α-EspP-C**

| | BamA | TamA | TpsB |
|---|---|---|---|
| 1 | GSFNFGIGYGT | NTIETGVGYST | VTGSVGID |
| 2 | GELSFSAGYSS | RTIELGAGYST | WRVAAGVN |
| 3 | GELSVGAGYSS | RTIGAAAGYST | HIRFGLIL |
| 4 | GELSFGLGFSS | RTIGGGLRYST | |
| 5 | GEFSIGGGYTT | HRFGVGGSYST | WQVNLSHD |
| 6 | GEFSIGGGYTT | RRFGFGAEYST | WHFSVANN |
| 7 | GSFNVSGGYST | HAVGVTGQISN | YEGYVLGD |
| 8 | GSFGIGAGYSQ | RYFGVGAQYST | FSGHIWTD |
| 9 | GSFGIGAGYAA | RYLGVGAQYST | WHLSFGNS |
| 10 | GELAFAAGYSS | NTISGEIGVST | VRLGMIVE |
| 11 | GSFQIGGGVSS | RTLGGGVSFAT | FSLSALVS |
| 12 | GSFGFGASYSV | RRLSAGIELSS | VTVRASVD |
| 13 | GEISIGAGYST | RFIGASAFYSS | VDALVSVN |
| 14 | GELTLSAGFSS | RTVAGLAGYES | VELVVGGQ |
| 15 | GAITLGAGFSS | NNIRGGVGFAT | VRFIATAD |
| 16 | GSLLLGATFSS | QQATFGVGASD | WGGEIRVD |
| 17 | GSLDLSAGWVQ | HKLETGIRLDS | IRFSIGID |
| 18 | GSIMFGAGLSS | IKLGVGAGYST | IRVSFTLD |
| 19 | GNIMLGAGFSS | RKLGFGIGVST | FRLSVAVD |
| 20 | GSINAGIGYGT | HEIETGIGYAT | LQGALEFD |
| 21 | GSLSASVGFSQ | HSVGVGGGFST | RETLLRLD |
| 22 | GSITFGLSYGM | RTLSLGVGYSS | YQFSFLVD |
| 23 | GTIGGTVGYAQ | RTIRLGVGYTS | WRLVTRMD |
| 24 | GSFSVGVGYSQ | TLYSFGLGIGT | LRLSLAID |
| 25 | AEASASIGYGT | YSYTLGAGYGT | VSGHAEYD |
| 26 | GNLMAGFGFSQ | RHYAIGIGYDT | YQFTALMD |
| 27 | GTTTLAVGYSQ | NSLETGVGYGT | VNFNLSVD |
| 28 | AQLGGGVGYSE | YSGEVY--YST | VGYDFAVD |
| 29 | GSVSASIGFSQ | HQFEVGVGYAT | LRPGWSLD |
| 30 | GSINFGIGYGT | NAMELGVGFST | FSSYVSVD |
| 31 | GSITASVGFAQ | RTMGLGLGFST | WRAGLSRS |
| 32 | GTLQFGVGFSD | QSYQISGGYGT | |
| 33 | GSLSAGIGFSQ | QLYTASIGYGT | FRLTLAFD |
| 34 | GSINFGIGYGT | NQIETGLGYST | IEPDLSYS |
| 35 | GSLMFGVGYSQ | TIYTGGPFFGT | FNATAGLD |
| 36 | GQLTAGLGFSQ | NRYRIGAGFST | DVQRVSLD |
| 37 | GSISLGAGYST | RTVKGGVDYKT | WRILLGWD |
| 38 | GTFSVGAGYSS | RTVRTGVGYGT | LHGSLELN |
| 39 | GNIMVGGGYGS | SHSRIGVGYAT | YNGYVIAD |

## Fig. S9a, logo plot input

| | BamA | TamA | TpsB |
|---|---|---|---|
| 1 | FQFNIGK | LQFYIGL | TVYWRVAV |
| 2 | FRFSTST | FQIYLSI | TVLFSASV |
| 3 | FQFNIGT | VQIYISL | QSLYFSLG |
| 4 | FRFSAGT | FEFYISI | |
| 5 | FNFGVST | LGFYIGI | MAFVSMSV |
| 6 | FNFGVST | YGLYVGI | LFFVSTSL |
| 7 | FRFTGGT | YALFIGI | RTKALVQI |
| 8 | FRFGMSN | FGIYAGI | QFWLSGRV |
| 9 | FKFGINN | YGIYAGI | LIFVSSSV |
| 10 | FRFSTRT | VQVYISI | QSAYLVIG |
| 11 | FRFSAGS | FEIYIGL | LIFSVAME |
| 12 | FDVTIST | LQFYIGI | NRFNLRWT |
| 13 | FRFSFGT | WQLYLSL | VFFNITAQ |
| 14 | VTFNVGT | ITVSVAL | LLTFSTRL |
| 15 | FQFQIGT | VRPYLTL | RSTCR--- |
| 16 | FQFQIGT | WRLHFNV | SDPGAGAM |
| 17 | FQFQLGT | IRWHISL | YGFNLNYS |
| 18 | FQFQMGQ | FHFHFNL | AGFNLSWS |
| 19 | FQFQMGT | LRLHFSV | GGFNLIWA |
| 20 | FSFNIGR | LRLHFAL | VLYYRASV |
| 21 | FQFSLGQ | WRIHFSM | ALYGYLST |
| 22 | FQFSIGI | VKLHLNI | IGFSAGYT |
| 23 | FQFSLGN | YQIYVSL | TVYADFSY |
| 24 | FQFAIGT | FRIHFTL | QAGFQAGI |
| 25 | FQFALSS | PRLVISM | YRYWVQGG |
| 26 | FQFSMGS | FQIHFAA | IHFQVRLD |
| 27 | IQFEIGR | IRLHFFI | WITGFSIN |
| 28 | FQFSVGQ | WRVHIQL | QAYFRFGV |
| 29 | FQFSLGQ | WRLHFAI | DVGLSVNL |
| 30 | FQFSIGG | IQFYIGL | QFWSMIKT |
| 31 | FQFSLGQ | VRLHFSM | IYFRLDFF |
| 32 | FQFSFGV | FRIHFSL | |
| 33 | FQFSLGS | IALHIVI | VFAGKSLS |
| 34 | FTFTIGR | FRIHFTL | LYNISASW |
| 35 | IQFTFGN | VQLHVVI | TRVNLTFS |
| 36 | FQFTFGT | FRLHLVV | TIYASLTQ |
| 37 | IGFSMGQ | AQFYCSI | EIYSAVTV |
| 38 | LEFSIGS | FRIHFSV | YLHFKVKW |
| 39 | IDFSIGT | NAIHFQI | FMFQLGWI |

# Fig. S9b, logo plot input

| | BamA | TamA | TpsB |
|---|---|---|---|
| **1** | MPFYENFYAGGSSTVRGFQSNTIGPKAV | VPPDLRFFAGGDRSIRGYKYKSIAPKYA | LHGVEQLSLGGESSVRGFKEQYISGNNG |
| **2** | VRINDRFYRGG-TSFRGFEIAGIGPRDI | VPASRRFFSGGGGSVRGYAYQAIGPRLS | LTTPDQYVIGNQTIGRGYQPGSAFADSA |
| **3** | VRINDRFFLGN-PRIRGFEQAGIGPRDD | LPATRRFYAGGGGSVRGYGFQRVGPVDA | GAIIAPFSLGGFQRLSGLSRDRLSGGFI |
| **4** | PRTTDRFFIGG-SDFLGFDRSGLGPRDT | IPAPRRLYSGGGGSVRGYQSRFIGPLDA | |
| **5** | VRIFDMFKSNS-DMIRGFKYNGIGPRQR | IPSDMLFFAGGGGSVRGYGYRNIGIRTD | LFGAEQMSLGGYSNIRGMRDSLLYGNNG |
| **6** | VRIFDLFKNNS-DIIRGFKFNGIGPYQE | LPPSQLFLAGGGGSVRGYGYRNIGVSAG | LYGAEQISLGGYSNVRGTRDSILFGNNG |
| **7** | LRVFDNFNLGP-SLVRGFAPGGIGPRDI | IPASHRFFAGGGGSVRGYQYRSLAPQYG | LDASEKIEVTGFYGVKSYDEGVAGDSGY |
| **8** | LLVFDQFKFGG-RQVRGFKNDGIGPRIG | IPTTRRFYAGGGGSVRGYSYQEISPYNA | LDSSEEIALGGPYAVRGWPIGEASADTG |
| **9** | LNVFDQFTLTN-GDIRGFENKGIGPRIA | IPATRRFFAGGGGSVRGYGYQEISPRNA | LFGAEQISVGGYSSVRGTRETMLYGNNG |
| **10** | VRINDRFFKGG-SSFRGFDVAGVGPRQL | VPLNRRYYAGGGGSVRGFGYQTISPLDA | PPIDTLYRIGGLFSLSGYRLDELAGENF |
| **11** | VRLNDRFFLGG-NRFRGFDVAGVGPRLF | IPATERFYAGGGGSLRGYGFQEASPIDP | KPVSQQCEYGGTDIGRGYDPLEASGDDC |
| **12** | STVNDRYFLSS-RQLRGFAYKGVGPRDF | TNPDLLFFSGGADTVRGQPYESLGIPVG | LASSEQMGLGGFDWLSAFEGSALQGDGG |
| **13** | IALSDRFFLGG-DTLRGFNTAGIGPRDL | VPPDHRFYAGGGGSVRGYGFQKAGPRDI | LLASEQIALGGPSYGRAFDEGEISGDSG |
| **14** | IRITDRYYLGE-PQFRGFDIRGVGPRVQ | IAPSRRFYAGGGGSVRGFGYQAIGPRDS | LLSFEQFTLGNYTVGRGYDPGTVQGDSG |
| **15** | YPIFKNYYAGGIGSVRGYEPSSLGPRDT | VPASLLFRAGGSNSVRGYGYQSIGNSVD | APSTEFMQIGGRYTVRGFDNHTLAGASG |
| **16** | YPVFKNFYGGGLGTVRAFDQNSLGAVDV | VPFTLLFRAGGDDSVRGYSYQTLGPTDA | LDPSKKWVLGGPNGVRAWPASEGSGDDG |
| **17** | IPFFENFYGGGLGSVRGYESGTLGPKVY | VPSGLMFRSGGASSVRGYELDSIGLAGP | LVAQDKLSIGSRYTVRGFDEQSLFGERG |
| **18** | FPFFKNFYLGGVNSVRGYRLSSIGPVCL | APASFLFRAGGDQSVRGYAYQSLGVKQA | LTPQDRFSIGSRFTVRGFDQQTLIADNG |
| **19** | LPFFRHFYAGGIGSVRGFKTGSLGPHDV | IPSDYLFRTGGDQTVRGYAYQGLGITAG | LVPQDRFAIGGRYTVRGFDESVLAAERG |
| **20** | LPFFENYYAGGFDTVRGFKSNTIGPKAI | VPPSLRFFTGGDQTVRGFGYESISPTGP | LYSVERLSLGGEASVRGFKEQSISGDVG |
| **21** | MPFYEHFFSGGYGSVRGYEANSLGLNAT | VPPTLRFFAGGDQSVRGYGYRSLGPTNS | LPASERQSLTGPSQVRGFDAGLYSADSH |
| **22** | LPFFENFYAGGLGSVRGYRGSSIGPKYE | LPPSLRFYTGGDNTVRGYKYDGIGEKGY | LISLDLLSIGGRNSVRGFSNNSISGDSG |
| **23** | LPFFENFYGGGFGSVRGFERNSLGPRST | IPADERFYAGGGGSVRGYAYQALGPRRL | LPSFEQFSLGGANGVRAFDVRDFSADQA |
| **24** | LPPFRNYYAGGIRSVRGFEDYSLSSGSD | APVSLRFFAGGDLSVRGYGYKSLGPVND | LIPQDRFAIGSRHSVRGFGELLLSGDRG |
| **25** | LPFFENYYAGGPGQVRGFDSFSLGPLDN | QPISLALLLGGMDNLKAFGFNSIGPGRI | LDSSQQFTMGGPFSMPGYPVGEVFGDLG |
| **26** | LPFFQNFYAGGVQTLRGFNDNTVGPKDS | LPTSYRFYAGGINSIRGYSYKELGPKDN | LLPLERIAVGGRYSVRGYRENQLVRDNG |
| **27** | LPFYKNFYAGGYGSVRGYDNSTLGPKYA | VPYNLRFFTGGDQTIRGFDYKSLSPEVD | LVPNDRFYIGGRYSVRGFDELMLSGDNG |
| **28** | LPPYRQFYGGGPETVRGYKESRLGPKDD | LPPDLRFFAGGDRSIRGFDYHEIGETNA | LVSLEQVSLTGPDAVRAYRTESALADTG |
| **29** | FPFYENFYAGGLGSVRGFTANTLGERTT | IPPSLRFFTGGDRTVRGYSYESLAPRNE | LSPLERFSIGSRYTVRGFHDTSLSAERG |
| **30** | LPFYQTYTAGGIGSLRGFAYGSIGPNAI | IPPTLRFFAGGDRSVRGYGYKKIAPKNR | LESSQKMLLGGLSGVRGHQAGAASVDEG |
| **31** | LPFYENYYAGGFNSVRGFKDSTLGPRST | VPPSLRFFAGGDQSVRGYEYQTLSPEND | LFSPQRTSLGGSASIRGYKDQSLSGDSG |
| **32** | VPPYDNFFAGGARSVRGYSNGGLGPRDS | LPPSQRFFTGGDRTVRGYGYQQISPENQ | |
| **33** | FPFFQNFYAGGIRSVRGFQANTLGVKEN | LPSSLRFFTGGDNSIRGFDYQSLGPEDS | LVPQDRFSIGGRYTVRGFDEQSLSAEHG |
| **34** | FPFYENYYAGGFSSLRGFGSNSAGPKAV | VPPSLRFFVGGINSVRGYGYEVISPEDE | LVSAERVSYGGRFYGRGYPGQAEGDVGY |
| **35** | FPFWENYYGGGVRDVRGFQDNTLGPRLC | LPPQLRFFAGGDRSVRGYSFESIGPRNQ | LPSTEQINFGGPRYAYAYDPGDAAGDSG |
| **36** | LPYWENYFAGGTKSVRGYKDYSLGPRDS | LPATRRFFAGGDNSIRGFDLDELGPKDS | LLSSDLLSVGGYYSVRGFQPAETTGEHG |
| **37** | IPVYERFYLGGINNVRGYELDKISPKDP | VTPDLRWYAGGSGSIRGYSYQSVGPMRG | MYSAERMSIGSRYTVRGFSADSLSGDSG |
| **38** | IPIDEKFYLGGISSLRGYSARTVSPSRT | IPPSIRFFAGGDQSVRGYSYQGLGPRNA | LISNEQYVAGGAESVRGYHESEASGDKA |
| **39** | ISIGERFYMGGIGSVRGFQINSVGTKDS | LPESKKFFAGGAFSNRAYGYDRIGVISS | LDGSEDLSLGGENGVKLYPNGEESAENG |

**Fig. S9c, logo plot input**

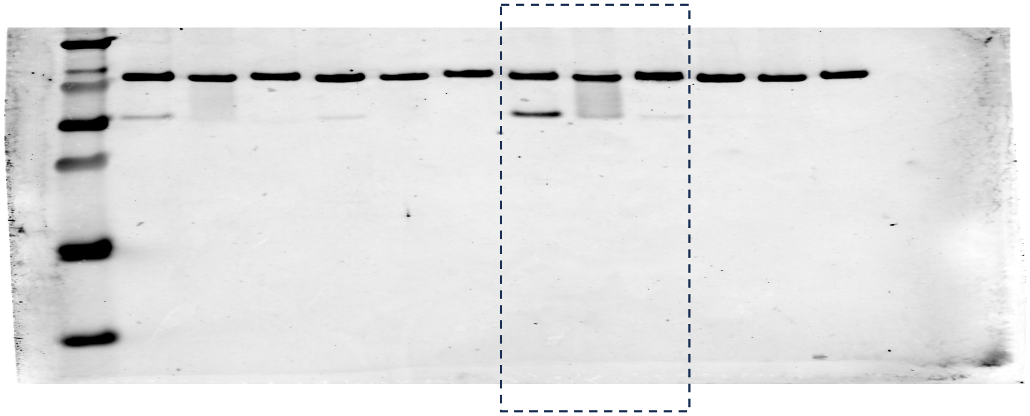

**Fig. S10a, top blot 1, α-OmpA-C**

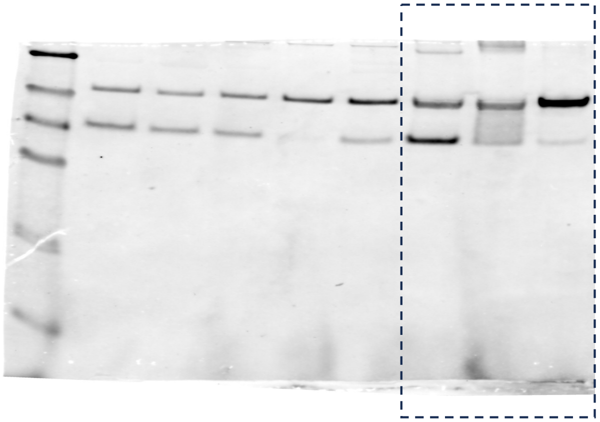

Fig. S10a, top blot 2, α-OmpA-C

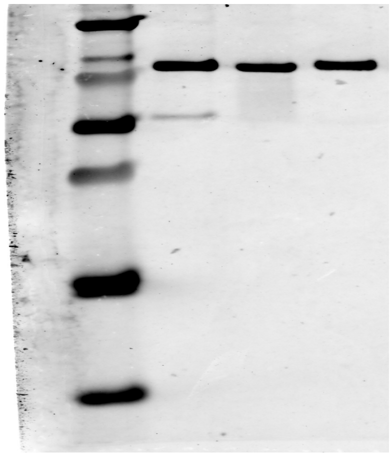

**Fig. S10a, top blot 3, α-OmpA-C**

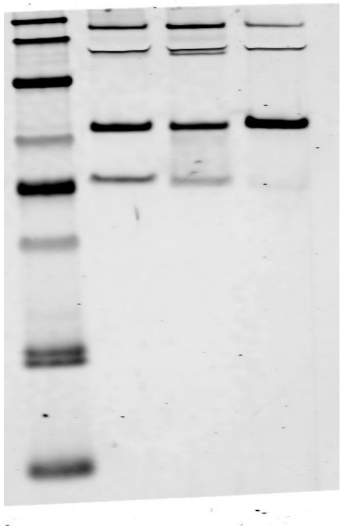

**Fig. S10a, top blot 4, α-OmpA-C**

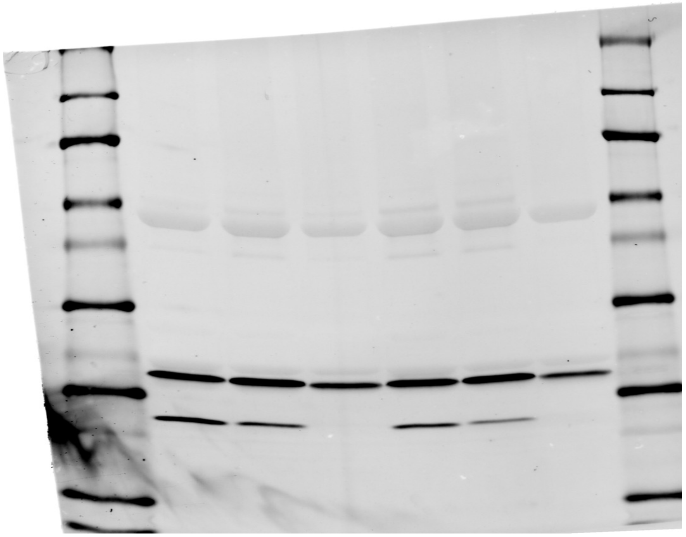

**Fig. S10a, bottom blots 1-2, α-EspP-C**

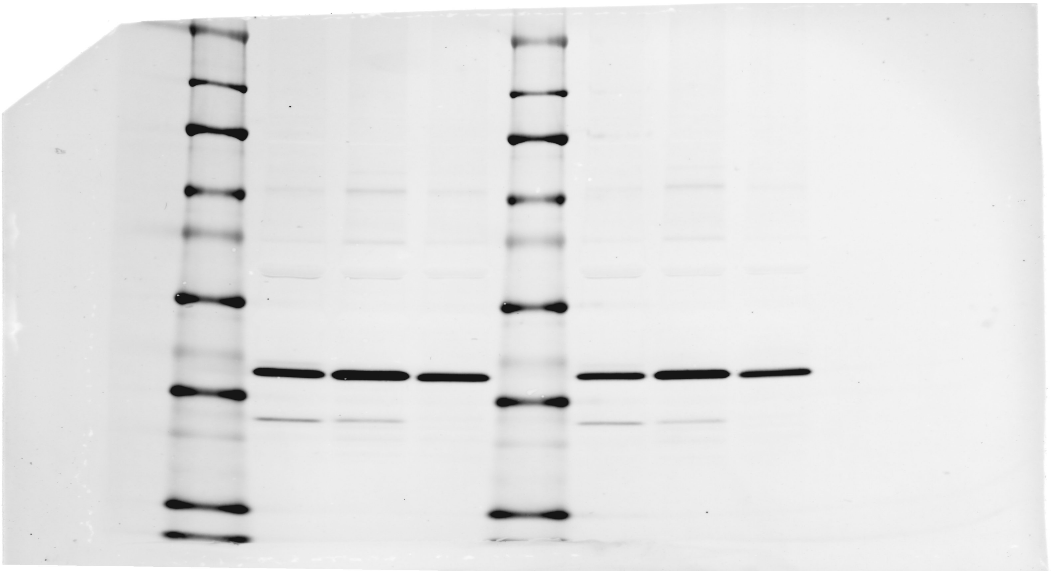

**Fig. S10a, bottom blots 3-4, α-EspP-C**

TAM/PLE + OmpA

| Darobactin : PL | Test-1 | Test-2 | Test-3 | Average | Standard Error |
|---|---|---|---|---|---|
| **1:1** | 90% | 71% | 69% | 77% | 0.0662 |
| **10:1** | 56% | 80% | 61% | 66% | 0.0723 |
| **100:1** | 26% | 33% | 35% | 32% | 0.0279 |

BAM/PLE + OmpA

| Darobactin : PL | Test-1 | Test-2 | Test-3 | Average | Standard Error |
|---|---|---|---|---|---|
| **1:1** | 94% | 102% | 97% | 98% | 0.0228 |
| **10:1** | 48% | 76% | 74% | 66% | 0.0921 |
| **100:1** | 5% | 26% | 24% | 18% | 0.0678 |

TAM/PLE + EspPΔ5'

| Darobactin : PL | Test-1 | Test-2 | Test-3 | Average | Standard Error |
|---|---|---|---|---|---|
| **1:1** | 58% | 75% | 84% | 72% | 0.0766 |
| **10:1** | 20% | 12% | 32% | 21% | 0.0578 |
| **100:1** | 0% | 0% | 0% | 0% | 0.0000 |

BAM/PLE + EspPΔ5'

| Darobactin : PL | Test-1 | Test-2 | Test-3 | Test-4 | Average | Standard Error |
|---|---|---|---|---|---|---|
| **1:1** | 97% | 80% | 84% | 92% | 88% | 0.0431 |
| **10:1** | 28% | 38% | -- | 45% | 37% | 0.0508 |
| **100:1** | 0% | 0% | 0% | 0% | 0% | 0.0000 |

**Fig. S10b, data**

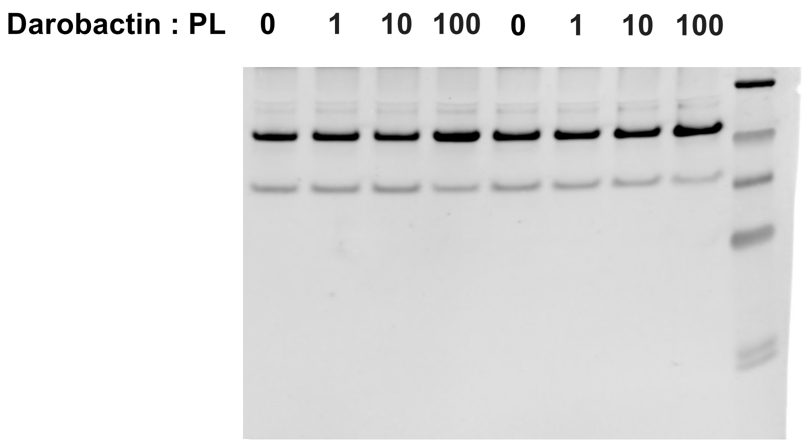


**Fig. S10b, repeats for TAM/PLE + OmpA, α-OmpA-C**

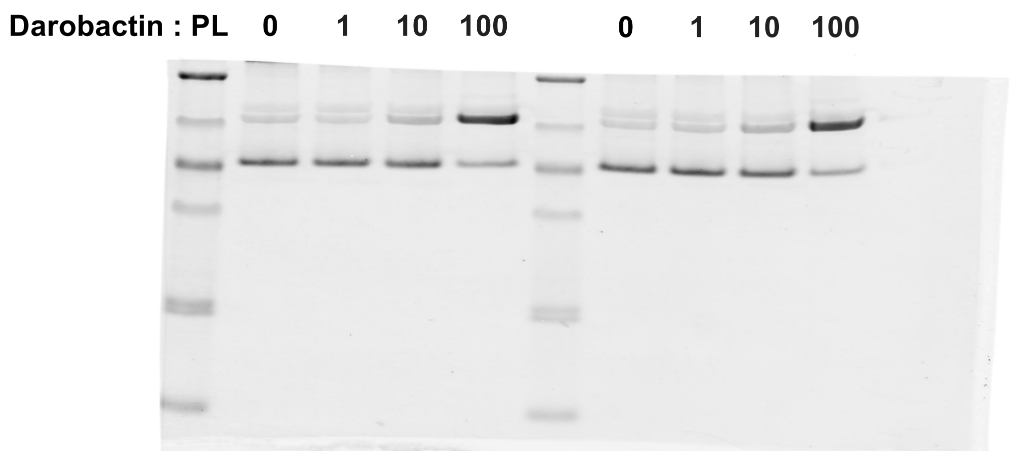


Fig. S10b, repeats for BAM/PLE + OmpA, α-OmpA-C

Darobactin : PL 0 1 10 100 0 1 10 100

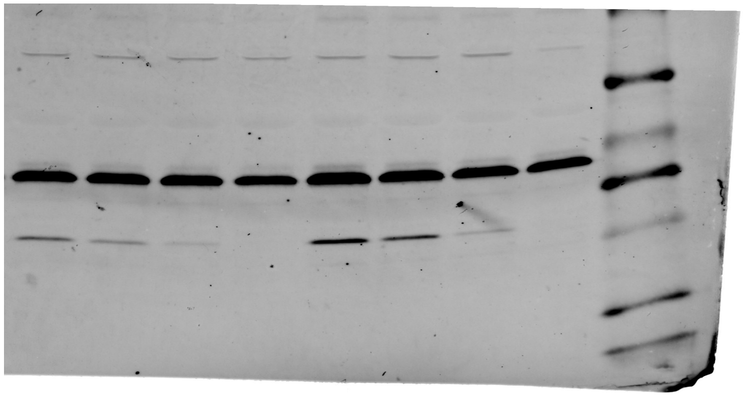

**Fig. S10b, repeats for TAM/PLE + EspPΔ5’, α-EspP-C**

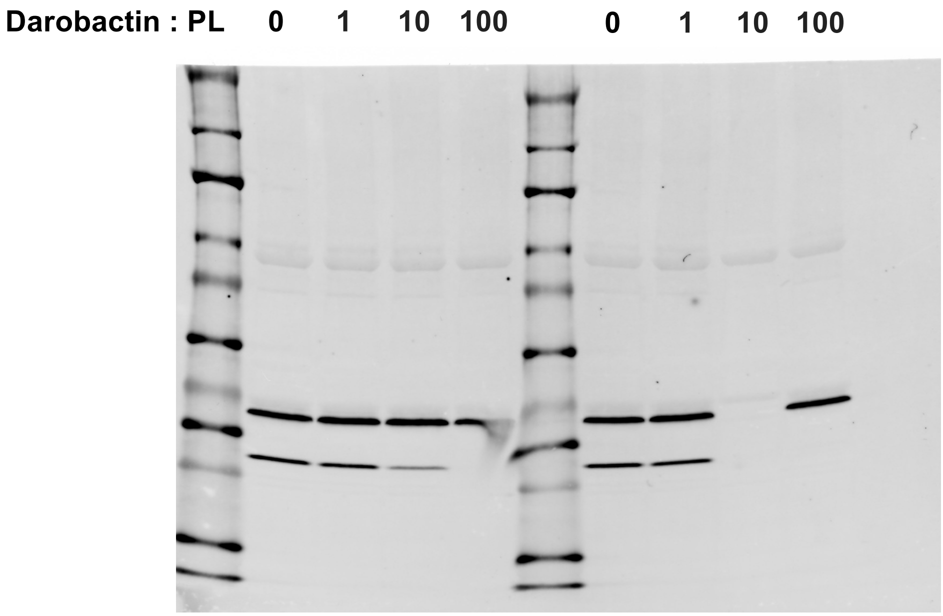


**Fig. S10b, repeats for BAM/PLE + EspPΔ5', α-EspP-C**

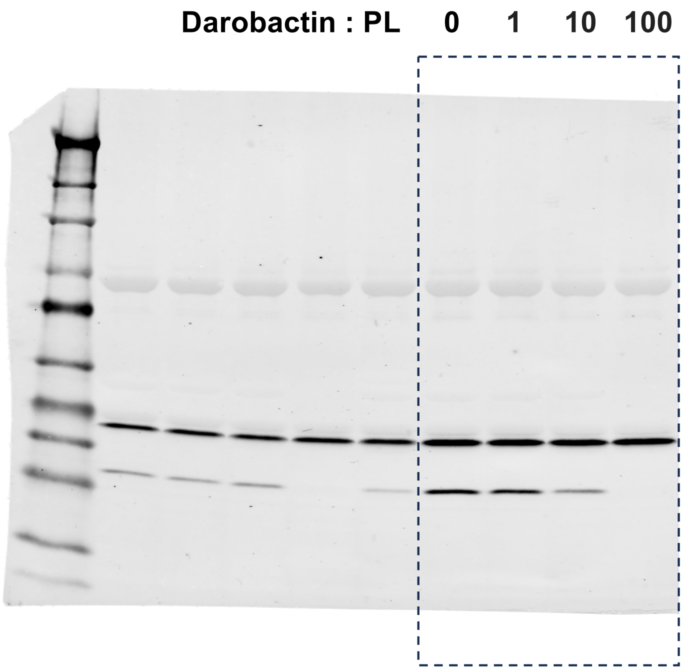


**Fig. S10b, repeats for BAM/PLE + EspPΔ5', α-EspP-C**

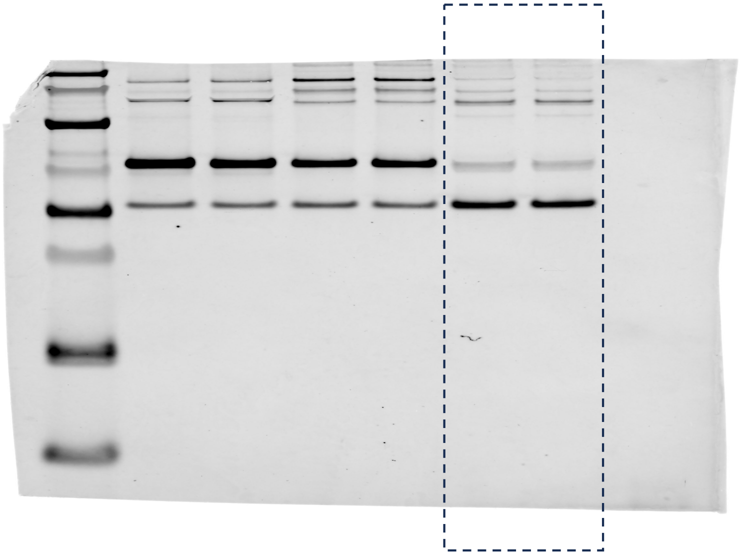

**Fig. S10c, top left blot, α-OmpA-C**

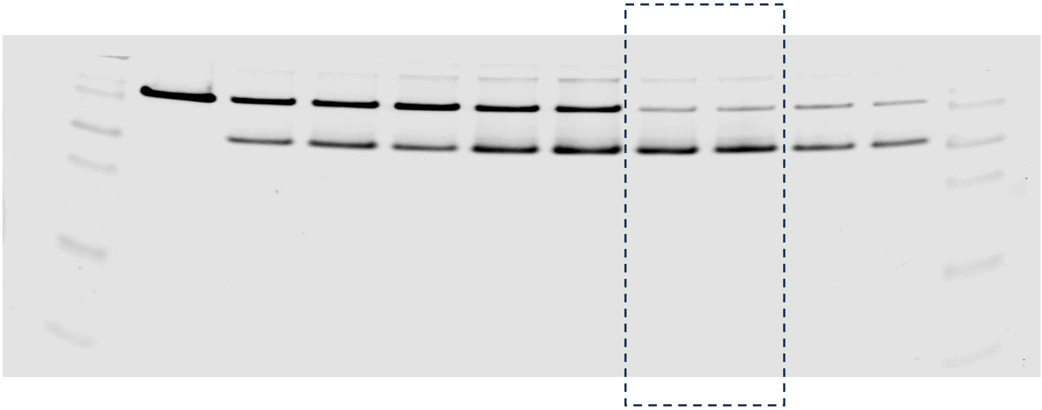

**Fig. S10c, top right blot, α-OmpA-C**

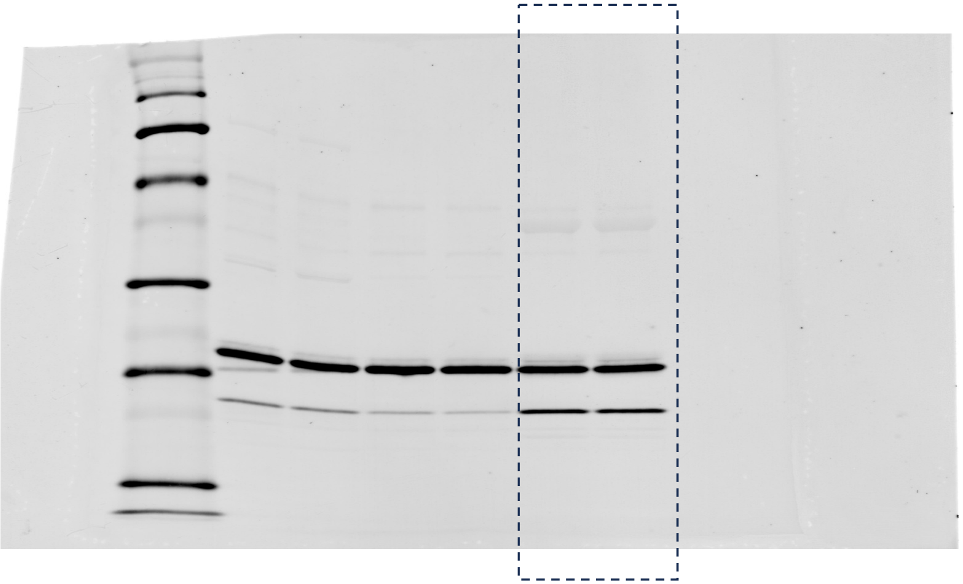

**Fig. S10c, bottom left blot, α-EspP-C**

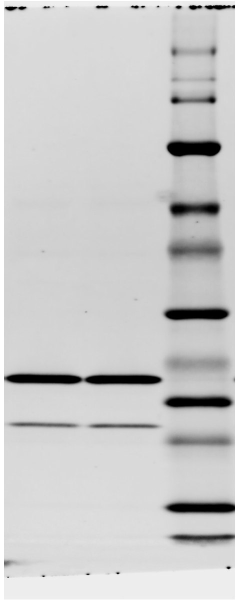

Fig. S10c, bottom right blot, α-EspP-C
